# Tunable and Photoactivatable Mimics of Calicheamicin γ_1_ for DNA Cleavage

**DOI:** 10.1021/jacs.4c07754

**Published:** 2024-09-09

**Authors:** Benjamin Ben-Zvi, Christina Lian, Maureen F. Brusco, Tianning Diao

**Affiliations:** † Department of Chemistry, New York University, 100 Washington Square East, New York, New York 10003, United States

## Abstract

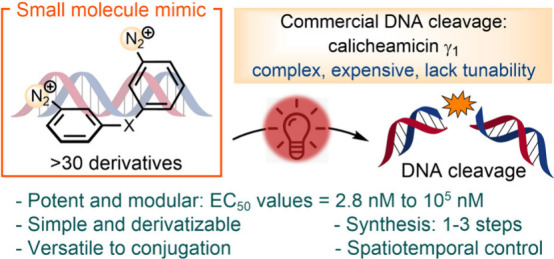

Calicheamicin γ_1_ and related natural products are renowned for their potency in DNA cleavage, serving as the warheads in commercial ADCs used for treating leukemia. Their mechanism of action involves the formation of aryl radicals, which abstract hydrogen atoms from nucleic acids. However, the complex strained enediyne structure of calicheamicin γ_1_ presents significant challenges in synthesis, resulting in high production costs and limited structural and activity modularity for tuning the therapeutic window. This report describes the development of simple molecular mimics based on diazonium salts, synthesized in fewer than 3 steps, capable of generating aryl radicals upon green or red light irradiation. SAR studies conducted on over 30 analogues reveal a wide range of potencies in DNA cleavage, with EC_50_ values ranging from low nanomolar to micromolar. Forming benzenoid diradicals does not appear to be necessary for potent DNA cleavage; instead, DNA cleavage can be achieved with radicals distributed among different arenes when connected with proper linkages. The potency is influenced by electronic effects, stereochemistry, orbital orientations, the distance between multiradicals, and the number of diazonium motifs within the molecule. In addition to providing a more cost-effective, efficient, and modular alternative to calicheamicin γ_1_, this technology offers the potential for enhanced specificity through spatiotemporal control.

Gemtuzumab ozogamicin (Mylotarg) and inotuzumab ozogamicin (Besponsa) are FDA-approved antibody-drug-conjugates (ADCs) used for treating leukemia (Scheme 1).^1,2^ While these ADCs utilize different antibodies, they share a common warhead: calicheamicin γ_1_, a natural product responsible for inducing cancer cell death.^3,4^ The mechanism of action (MoA) involves the formation of 1,4-benzenoid diradicals through Bergman cyclization of the strained enediyne motif.^5,6^ The highly reactive aryl radicals undergo hydrogen atom abstraction from the C5′ position of 2-deoxyribose in DNA, forming 2-deoxyribosyl radicals. Trapping of the radicals by oxygen is followed by irreparable DNA backbone cleavage, leading to cell-cycle arrest and apoptotic cell death.^3,7,8^

**Scheme 1:**
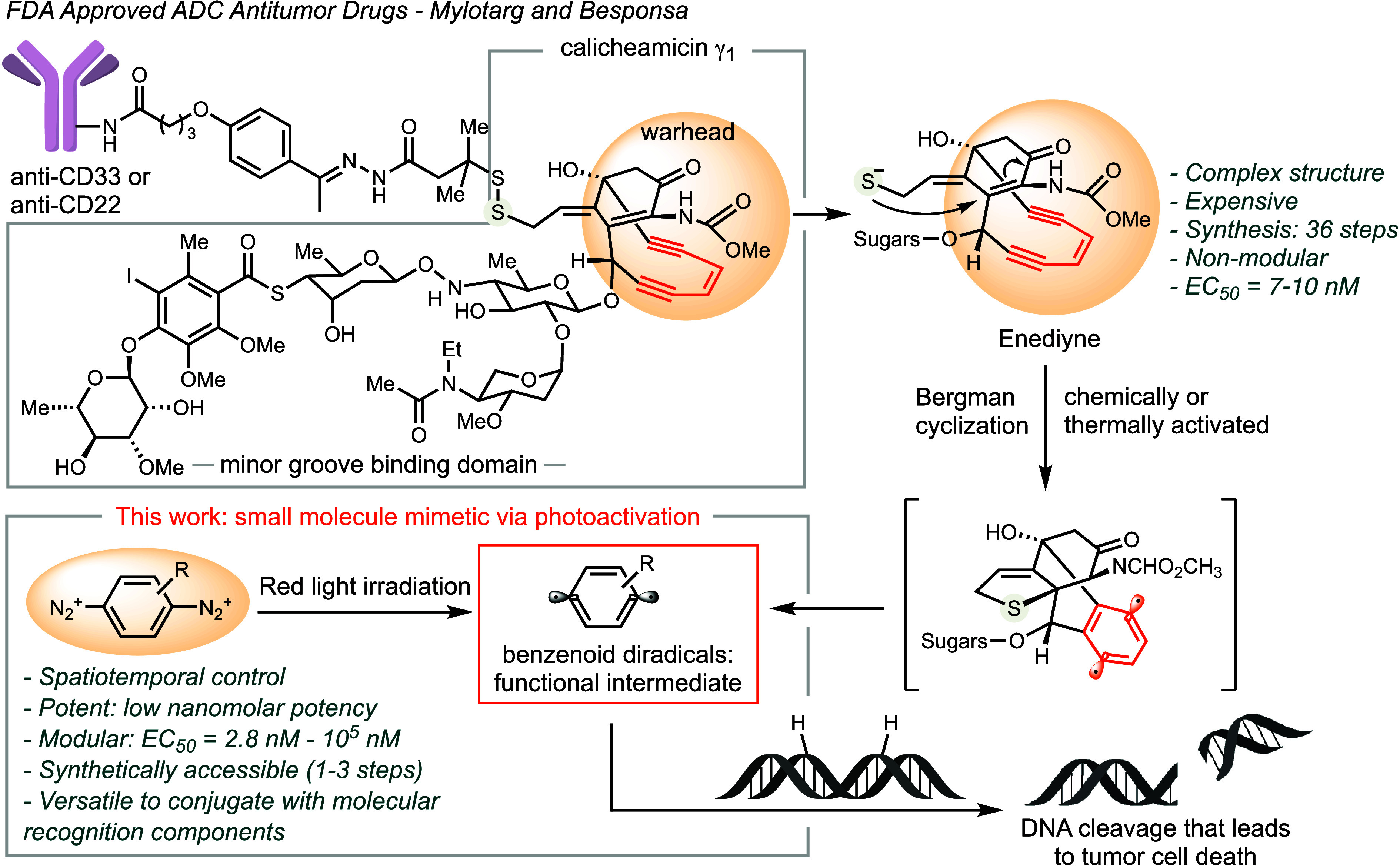
Diazonium Compounds as Alternative Warheads in ADCs for Cancer Therapeutics

The synthesis of calicheamicin γ_1_ and related natural products containing strained enediynes^9−13^ presents significant synthetic challenges due to the instability of the strained enediyne motif and the complexity of the molecular scaffolds.^14^ The total synthesis of calicheamicin γ_1_ requires 36 steps.^15^ Commercially, the production of calicheamicin γ_1_ relies on the extraction of the core structure from bacteria cultures, a process that necessitates rigorous and expensive purification and quality control measures.^16,17^

It is even more challenging to synthetically access analogues and derivatives of calicheamicin γ_1_ for adjusting its potency and efficacy to achieve a versatile therapeutic window. The modularity of payload toxicity is crucial for developing a successful ADC, as the toxicity should be within an optimal range rather than maximized.^18^ The lack of tunability in the toxicity of calicheamicin γ_1_ may have contributed to its severe side effects.^19^

A small molecule mimic of calicheamicin γ_1_, capable of generating aryl radicals, could address challenges related to production costs and tunability. While Nicolaou’s strained enediyne mimic exhibits high cytotoxicity,^20^ the simpler analogues are much less potent in DNA cleavage assays.^21−27^ Many synthetic mimics display EC_50_ values ranging from millimolar (mM) to micromolar (μM), which are significantly higher than the low nanomolar (nM) concentrations effective for calicheamicin γ_1_. Moreover, the synthesis of these mimics involves numerous steps, and some require transition metal reductants or catalysts for their activation, further limiting their potential as drug candidates.^28^

We hypothesize that the active species for DNA cleavage, benzenoid radicals, could be generated from diazonium salts through photoredox activation upon nitrogen extrusion (Scheme 1).^29−31^ The reduction potentials of aryl diazonium salts (*E* = −0.16 V/SCE) suggest the feasibility of activation using low-energy, long-wavelength light.^32^ Moreover, diazonium salts are readily available and inexpensive, derived from aryl amine precursors in just 1–3 synthetic steps, and offer a broad range of functionalities and structural motifs for activity adjustment. Besides providing a more economical, efficient, and modular mimic to calicheamicin γ_1_, this technology offers the potential to improve specificity via spatiotemporal control.

We synthesized a library of diazonium salts by converting the corresponding aryl amines under modified diazotization conditions.^33^ Diethyl ether or acetone as a cosolvent to water facilitated the precipitation of diazonium salts, which were obtained in yields ranging from 40 to 95% (Figure 1A). After washing with ether, the diazonium compounds were confirmed to be pure by ^1^H and ^13^C NMR spectroscopy and were used without further purification.

**Figure 1:**
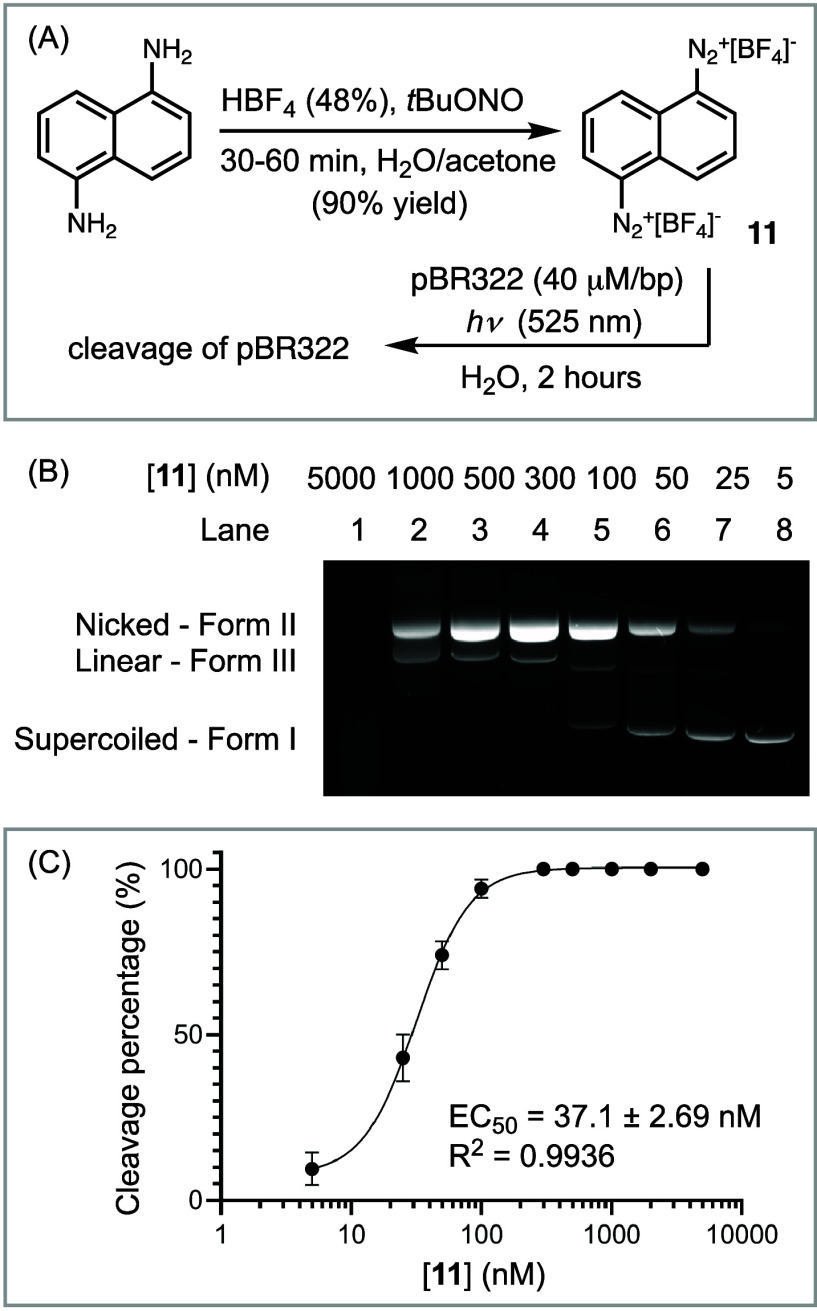
(A) Synthesis of diazonium compound **11** and conditions for DNA cleavage. (B) Agarose gel-electrophoresis of the pBR322 DNA cleavage (40 μM/bp) treated with **11** (2 h, 22 °C). Form I = supercoiled DNA; Form II = nicked DNA; Form III = linear DNA. (C) Dose–response curve from which EC_50_ was determined.

We used bis-diazonium **11** as a model compound to develop the protocol for evaluating its DNA cleavage potency. We employed a common assay, using supercoiled DNA pBR322 (Form I), which can undergo single and double-strand cleavage, resulting in nicked (Form II) and linear (Form III) forms, respectively. On agarose gel-electrophoresis, the various forms of pBR322 travel different distances, calibrated through cleavage with digestion enzymes such as the nicking enzyme, Nb.BtsI, and the double-strand cleaving enzyme, EcoRV-HF (Figure S1). We incubated pBR322 with **11** at room temperature in neutral pH water, in the presence of 525 nm green light irradiation for 2 h and observed the cleavage of pBR322 by gel-electrophoresis (Figure 1B). By varying the concentration of **11** in the presence of a standardized 0.5 μg of DNA (40 μM/bp), we generated a dose–response curve from which we derived an EC_50_ value of 37.1 nM (Figure 1C). At a low [**11**], the supercoiled pBR322 underwent single-strand cleavage to afford Form II. As [**11**] increased, Form II underwent double-strand cleavage to yield Form III. Further increase in [**11**] led to complete DNA degradation to low molecular weight fragments.

We investigated a library of diazonium compounds to determine the structure–activity-relationship (SAR) (Scheme 2). Most diazonium compounds remain stable in solid form for weeks and in aqueous solutions for longer than 6 h (Figure S12). Phenyl diazonium salt **1** exhibited limited activity with an EC_50_ of 18.8 μM. The more electron-rich analogue **2** showed even lower activity compared to **1**, while electron-deficient derivatives **3**-**5** decreased the EC_50_ by 2 orders of magnitude. This enhancement in potency by electron-withdrawing groups was also evident when comparing the naphthalene derivatives **6** and **7**, as well as the amide derivatives **8** and **9**.

**Scheme 2:**
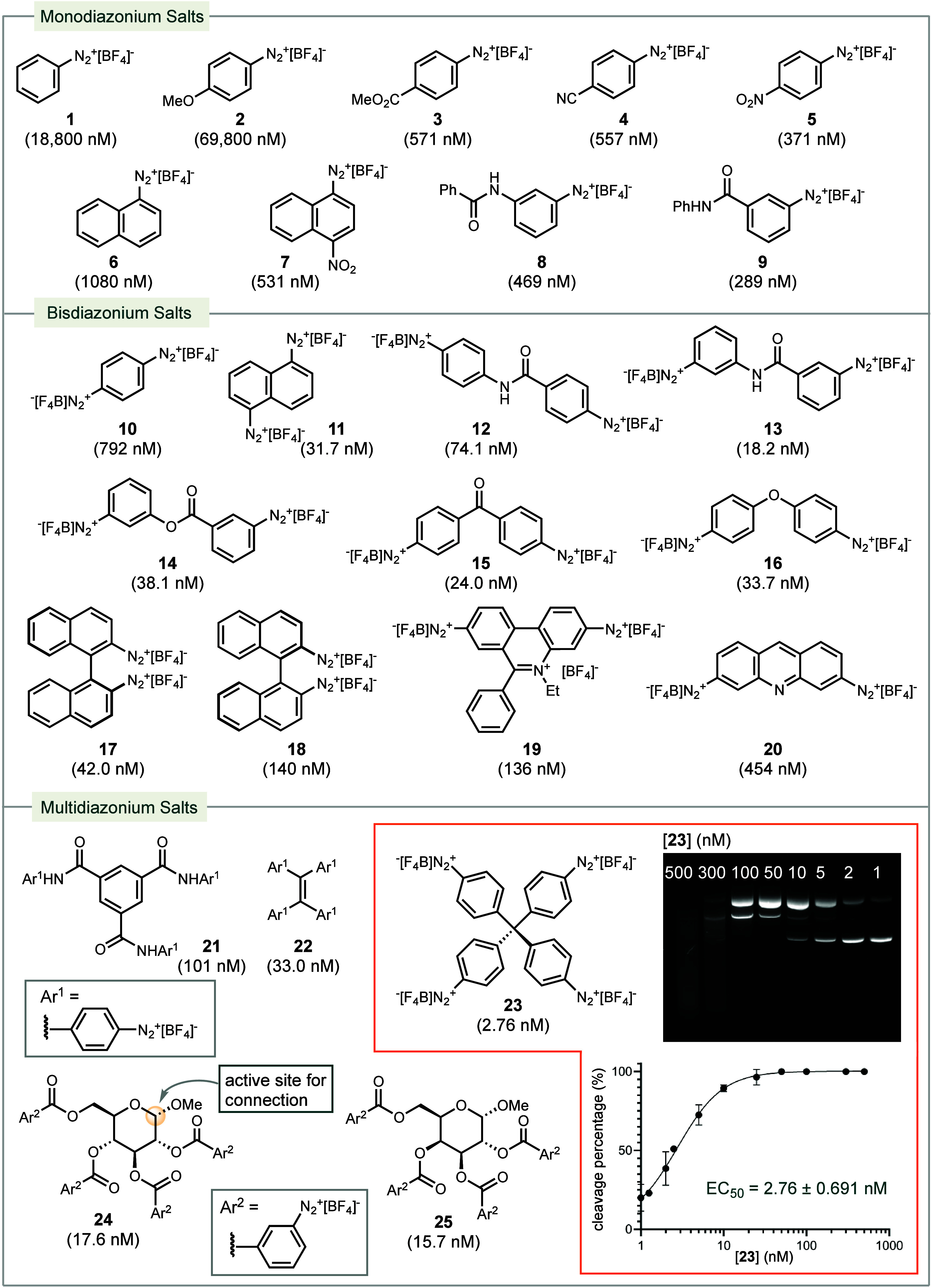
Potency of Diazonium Compounds for DNA Cleavage 1.5 h, irradiation (525 nm), water (pH = 7.0), 22 °C. Values were obtained from duplicate experiments.

To mimic the benzenoid diradical intermediate that arises from calicheamicin γ_1_, we explored bis-diazonium salts. Benzene-1,4-bisdiazonium **10** displayed improved potency compared to monodiazonium **1**. Similarly, naphthalene-1,5-bisdiazonium **11** outperformed **6**. In analogues **12**-**16**, where the two diazonium groups are distributed across two different arene rings, connected via ketone, amide, ester, and ether linkages, there was a notable improvement in potency, with EC_50_ values ranging from 18 nM to 74 nM. Notably, **13** was more effective than **12**, indicating that the orientation and distance between the two radicals play a crucial role in their activity. (*S*) and (*R*) 1,1′-binaphthyl derivatives **17** and **18** showed different potencies, suggesting that enantiomers are distinguished through molecular recognition via their interaction with DNA. The diazonium derivatives of acridine, known DNA intercalators,^34,35^**19** and **20** exhibited only modest potency, which are attributed to the misaligned orientation of the SOMOs (singly occupied molecular orbitals) relative to the C–H bond at C5′ of the nucleotides.^36^

Subsequently, we increased the number of diazo units in the molecule. Tridiazonium compound **21** showed no notable improvement, possibly due to the extended distance between the diazo units. Tetra-diazonium salts **22** and **23** displayed excellent potencies. Specifically, **23** exhibited an EC_50_ of 2.76 nM (2.14 ng/mL). In a preliminary cell viability study, we tested the antiproliferation activity of **23** on HeLa cells. Compound **23** proved to be cytotoxic with an IC_50_ of 6.71 μM (Figure S45). Additionally, we employed monosaccharides as scaffolds to host multiple diazonium moieties. Both **24** and **25** demonstrated very good potencies. The anomeric position in the carbohydrate framework serves as a handle for potentially linking to a minor-groove binder to enhance molecular recognition and achieve specificity.^37^ Moreover, this handle opens avenues for connecting the diazonium compounds as a payload to an antibody in further development of ADCs.

Control experiments shed light on the MoA (Figure 2A). We first evaluated the effect of light irradiation. In darkness, the EC_50_ value for DNA cleavage by **23** increased to 1 μM, substantiating the necessity of light for its activation. While a shorter wavelength of 467 nm provided activity comparable to that of 525 nm, a longer wavelength (660 nm) led to a slight decrease in reactivity. Performing the reaction with a degassed solution led to a significant 2-log scale decrease in activity (Figures 2B and S10), consistent with the participation of O_2_ in the cleavage process.

**Figure 2:**
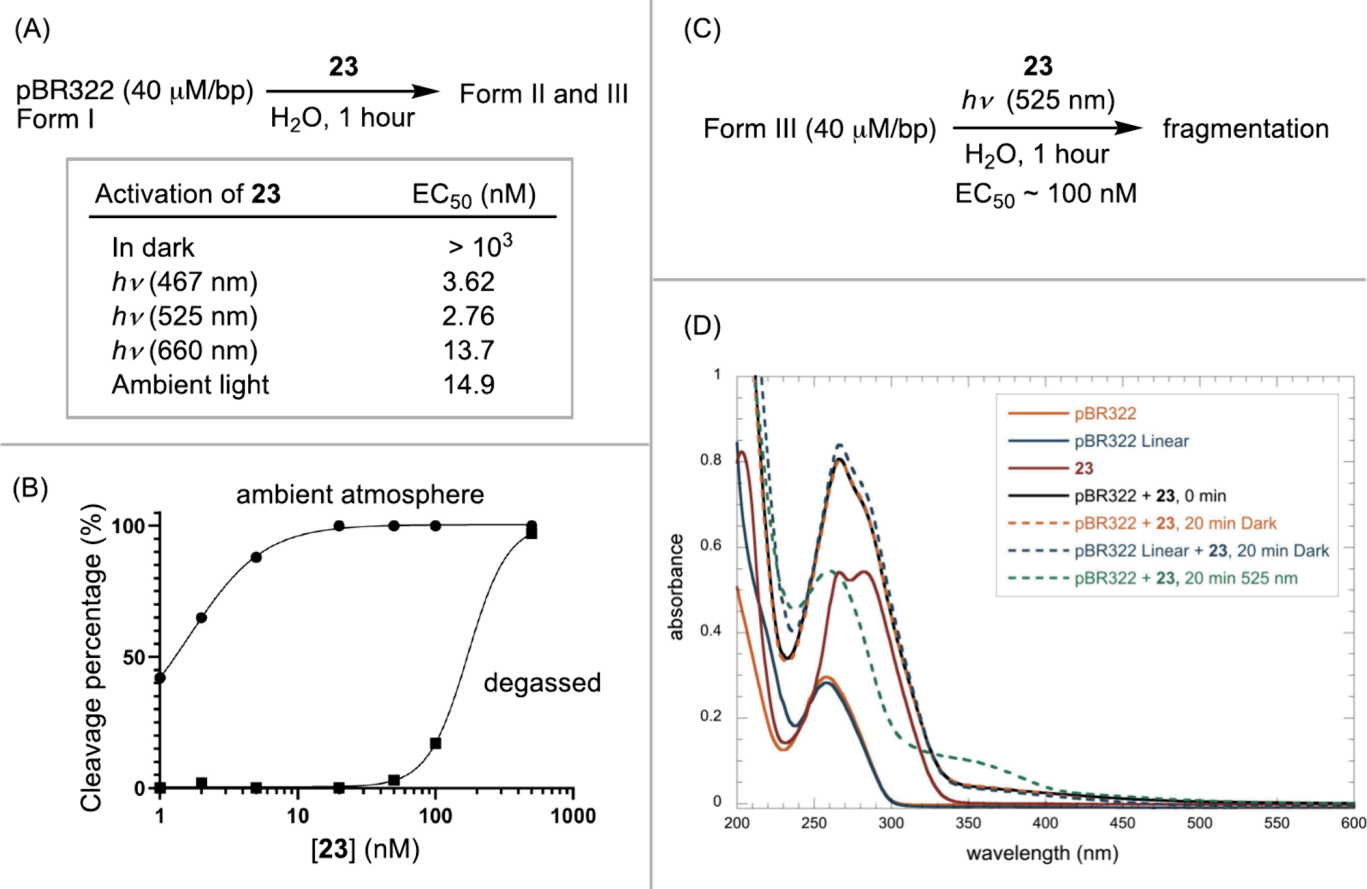
(A) Effect of light irradiation for **23**-mediated DNA cleavage; (B) DNA cleavage in a degassed solution; (C) Cleavage of linear pBR322 (form III) by **23**; (D) UV–vis spectroscopic study of the interaction between DNA and **23**.

Subjecting prelinearized DNA pBR322 (form III) to **23** resulted in noticeable cleavage into low molecular weight fragments at concentrations of 100 nM (Figures 2C and S11). The need for higher concentrations of **23** to cleave linear DNA, compared to supercoiled DNA, may be attributed to certain molecular recognition with supercoiled DNA. Alternatively, the inherent strain in supercoiled DNA makes it more susceptible to cleavage than its linear form.

We conducted UV–vis experiments to probe the interaction between diazonium compounds and DNA (Figure 2D). Neither form of pBR322 nor **23** alone absorbed above 350 nm. Mixing supercoiled or linear DNA pBR322 with **23** led to an increase in absorption between 200 and 300 nm and a redshift in absorption bands, extending up to 500 nm. Incubating this mixture in darkness for 20 min did not alter the absorption spectra. In contrast, exposure to 525 nm light for 20 min significantly changed the spectrum. The increase in absorption intensity upon mixing DNA with **23** suggests that intercalation is unlikely, as intercalation typically results in decreased absorption due to quenching effects caused by DNA.^38^ The redshift in the absorption bands supports an association between DNA and diazonium compounds, potentially explaining green light-promoted activation. Nonetheless, the activity of **23** triggered by red light irradiation remains unexplained by the UV–vis data, indicating the need for further investigation.

While the MoA for diazonium-mediated photoredox DNA cleavage remains elusive, we hypothesize that the ground-state association between supercoiled DNA and diazonium compounds facilitates inner-sphere charge transfer upon irradiation. This could lead to the reduction of diazonium salts by reductive components in DNA, such as guanine or adenine (*E*_ox_ ∼ 1 V vs NHE),^39^ although such charge transfer is typically unfavorable in an outer-sphere context determined by their redox potentials. Upon the extrusion of an N_2_ molecule, the diazonium compounds generate aryl radicals that initiate HAT on nucleic acids. The resulting radical intermediates generated on polynucleic acids are subsequently trapped by O_2_, leading to the oxidative cleavage of the DNA chain.

In summary, we explored diazonium compounds as simple molecule mimics for complex enediyne natural products, capable of generating aryl radicals under green or red-light irradiation for DNA cleavage. The straightforward synthesis and modular structures enabled SAR studies of over 30 analogues. Formation of benzenoid diradicals appears to be unnecessary to achieve potent DNA cleavage. Instead, radicals can be distributed among different arenes, provided they are connected through suitable linkages. The potency of these diazonium compounds depends on several factors: electronic effect, the distance and the arrangement of radicals, stereochemistry, and steric hindrances. This diverse array of diazonium compounds demonstrates a wide range of EC_50_ values, from 2.76 nM to μM levels, highlighting their potential as economical, potent, photoresponsive, tunable, and modular warheads for cancer and antibiotic therapies. Moreover, the high potency of multidiazonium compounds demonstrates that the biological activities of natural products may be replicated by synthetic mimics that target only the reactive intermediate. These mimics can achieve this through a different mechanism of activation from a simplified precursor.

## Supporting Information Available

All experimental procedures, gel images, and characterization spectra (PDF)
