# Supplementary material for: Tunable and Photoactivatable Mimics of Calicheamicin γ1 for DNA Cleavage
Source: J Am Chem Soc. 2024 Sep 9;146(37):25416–21. doi: 10.1021/jacs.4c07754 (PMC11421022; doi:10.1021/jacs.4c07754)
Supplement: Supplementary file 1 — ja4c07754_si_001.pdf [file ja4c07754_si_001.pdf]

Supporting Information

Tunable and Photoactivatable Mimics of Calicheamicin γ_1 for DNA Cleavage

Benjamin Ben-Zvi, Christina Lian,[‡] Maureen F. Brusco,[‡] and Tianning Diao*

[‡]These authors contributed equally.

Department of Chemistry, New York University, 100 Washington Square East

New York, NY 10003, United States

*E-mail: diao@nyu.edu

Contents

I. Materials and Methods 2

II. Additional Results on Diazonium-Mediated DNA Cleavage 3

III. Synthesis of Substrates 4

1. General Procedure for the Coupling and Reduction of Diazonium Precursors (S1-S7) 4
2. General Procedure A for Synthesis of Monodiazonium Salts 5
3. General Procedure B for Synthesis of Multidiazonium Salts 8
4. General Procedure C for Synthesis of Glycosyl Diazonium Precursors 14
5. Additional Diazonium Substrates 17

IV. Examination of DNA-Cleaving Ability of Diazonium Salts 20

V. Agarose Gel Electrophoresis 20

VI. DNA Cleavage Assay Controls and Restriction Enzyme Calibrations 20

VII. DNA Cleavage by Irradiation with Different Wavelengths and in the Dark 22

VIII. DNA Cleavage under Deoxygenated Conditions 27

IX. Cleavage Profile of Isolated Linearized DNA 28

X. UV-VIS Spectra for DNA and Diazonium Complexation 30

XI. Stability of Diazonium Salts in Water 31

XII. Gel Images and EC_{50} Plots 33

XIII. Cell Culture and Cell Viability Procedures 65

1. General Cell Culture Protocol 65

2. Cell Viability Assay and IC_{50} Procedure 65

XIV. NMR Spectra 67

XV. References 91

I. Materials and Methods

All chemical reagents were purchased from commercial suppliers (Ambeed, Oakwood Chemical, Sigma-Aldrich, TCI, Alfa, or Thermo Fisher Scientific). Agarose was sourced from Sigma-Aldrich and GelRed® DNA gel stain (10,000X in water) from Biotium. 50X Tris-acetate-EDTA (TAE) buffer was purchased from Thermo Fisher Scientific. Supercoiled pBR322 plasmid was purchased from New England Biolabs. Double distilled water was produced by ELGA Purelab Flex Water Purification System. ^{1}H NMR spectra were recorded on a Bruker 400 MHz and 500 MHz Avance spectrometer. Chemical shifts are reported in ppm relative to tetramethylsilane, with the residual solvent resonance (CD_3CN, δ = 1.94 ppm) as the internal reference. Spectra are reported as follows: chemical shift (δ ppm), multiplicity (s = singlet, d = doublet, t = triplet, q = quartet, m = multiplet), coupling constant (Hz), and integration. ^{13}C NMR spectra are recorded on Bruker 500 (126 MHz). Chemical shifts were reported in ppm relative to tetramethylsilane with the solvent resonance used as the internal reference (CD_3CN, δ = 118.26 ppm). ^{19}F NMR spectra were recorded on Bruker 500 (471 MHz). High resolution mass spectra (HRMS) were recorded on an Agilent 6224 TOF LC/MS (APCI source and ESI source). Infrared (IR) spectra were acquired using Nicolet 6700 FT-IR spectrometer through attenuated total reflectance (ATR). UV-Vis spectra were obtained using a Cary 100 UV-Visible Spectrophotometer. Substrates were synthesized according to literature and modified procedures. DNA gel electrophoresis were performed with Bio-Rad Wide Mini-Sub Cell GT Cell System. Agarose gels were pre-stained with GelRed (Biotium) and imaged by Bio-Rad ChemiDoc Imaging System.

II. Additional Results on Diazonium-Mediated DNA Cleavage

Scheme S1. Additional Data on Diazonium-Mediated DNA Cleavage[a]

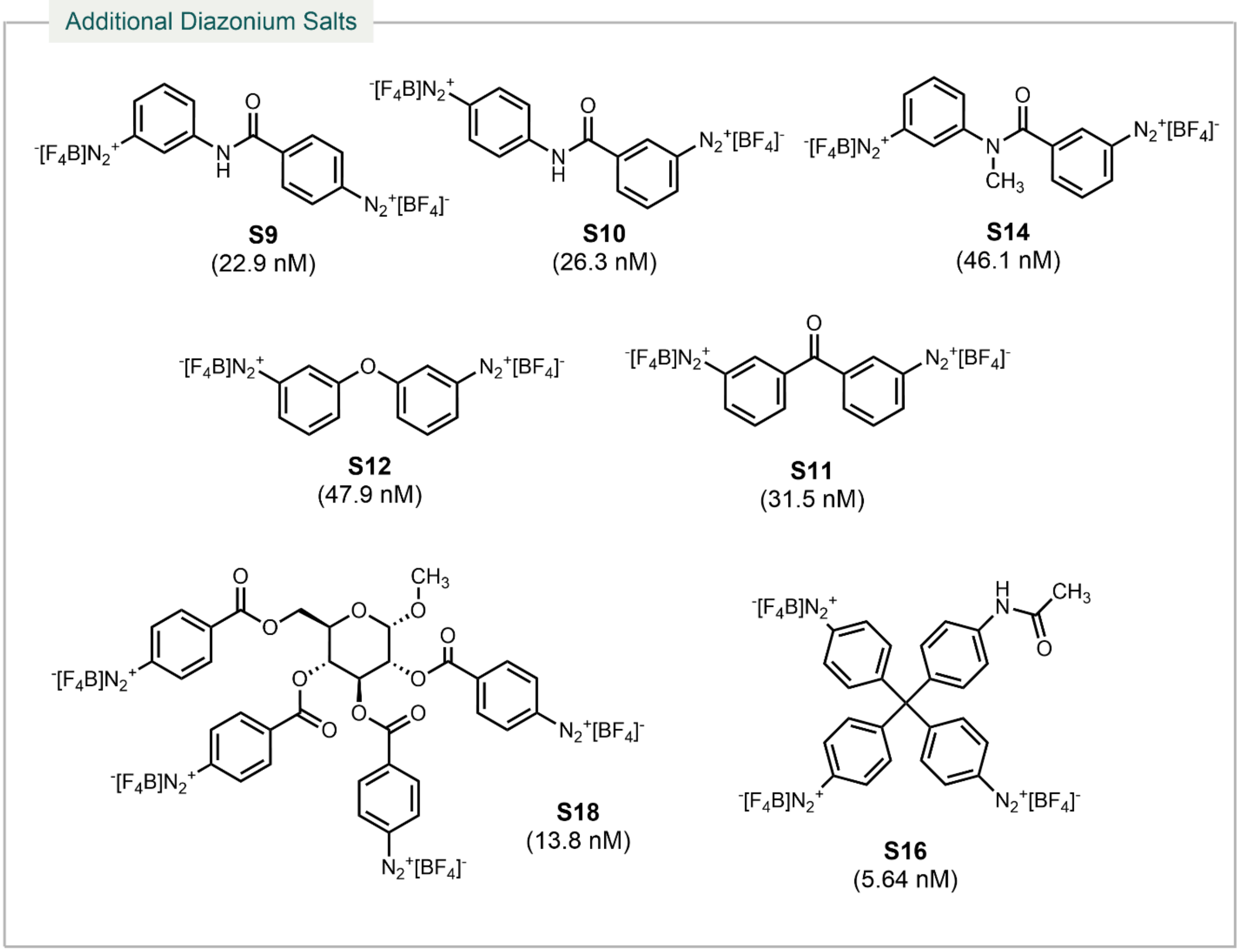

[a]2 hours, irradiation (525 nm), water (pH = 7.0), 22 ºC. Values were obtained from duplicate experiments.

Varying the positions of the diazonium functional group on amides **S9** and **S10** revealed similarly high activity compared to that of amide **13**, suggesting an optimal distance between the diazo units. To investigate whether the amides engage in hydrogen bonding with DNA, thereby facilitating its coordination to DNA and subsequent cleavage, we synthesized **S14** with methyl protection. Compound **S14** exhibited good potency. We attribute the slightly higher EC_{50} of **S14**, compared to those of **13**, **S9**, and **S10**, to the different dihedral angle of the two radicals, which is affected by the methylation of the amide. Ketone- and ether-linked diazonium salts **S11** and **S12** showed similar, but slightly lower reactivity than their *para*-substituted counterparts, **15** and **16**, demonstrating that while the electronic effect is significant for reactivity in bisdiazonium compounds, it is less pronounced compared to the trend found with monodiazonium species. Supporting this observation, monosaccharide **S18**, with the diazonium units *para* to the ester of the sugar, displayed potency comparable to that of **24** and **25**. Finally, we synthesized **S16**, where an aniline is protected by acetate. Compound **S16** showed excellent potency. The acetate protection suggests potential for future conjugation of this potent diazonium compound with a linker, opening avenues for exploration in ADC development.

III. Synthesis of Substrates

1. General Procedure for the Coupling and Reduction of Diazonium Precursors (S1-S7)

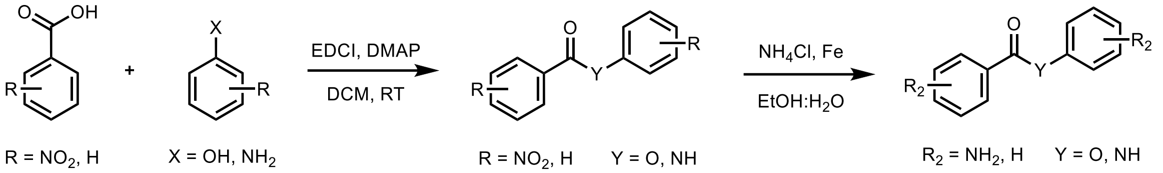

In a round bottom flask, EDCI (EDCI = 1-ethyl-3-(3-dimethylaminopropyl)-carbodiimide hydrochloride) (1.2 equiv.) and DMAP (DMAP = 4-dimethylaminopyridine) (0.1 equiv.) were added to a mixture of a corresponding carboxylic acid in DCM (0.2 M) at 0 °C. The mixture was removed from the ice-water bath and allowed to stir for 30 minutes. The subsequent aryl amine or alcohol (1.1 equiv.) was then added to the flask, which was then purged with nitrogen and allowed to stir for 16 hours. The precipitate was collected by filtration, washed with DCM, and dried over vacuum to yield the diaryl amide or ester product, which was used in the next step without further purification.

In a round bottom flask, the corresponding nitroarene was dissolved in ethanol (0.25 M). A prepared aqueous solution of ammonium chloride (10 equiv.) in the same volume as ethanol used, was added to the flask, followed by Fe(0) powder (10 equiv.). The flask was sealed with a condenser and refluxed in an oil bath at 100 °C for 3 hours. The mixture was allowed to cool to room temperature and was filtered through a layer of silica. The filtrate was concentrated by solvent evaporation. The resulting solid was washed with water and the solid was collected through vacuum filtration and washed with additional water to obtain the amine product without further purification. When further purification was required, the substrate was purified by flask column chromatography (hexanes:ethyl acetate 1:1).

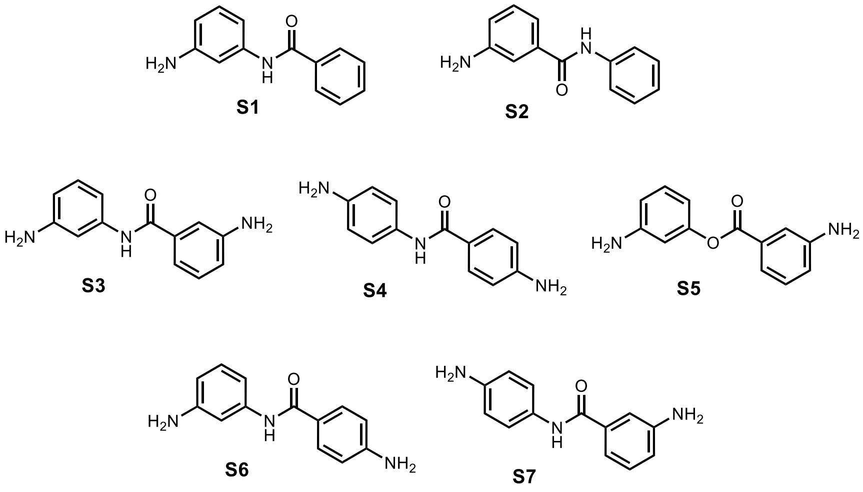

Scheme S2. Synthesized Diazonium Precursors.

2. General Procedure A for Synthesis of Monodiazonium Salts

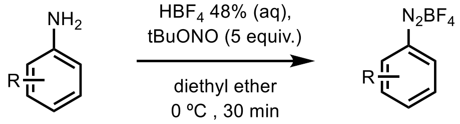

In a 20 mL glass vial, 1 mmol of corresponding amine was suspended in a mixture of 2 mL diethyl ether and 0.6 mL of a 48% aqueous solution of HBF_4. The vial was placed in an ice-brine bath and tert-butyl nitrite (5 equiv.) was added dropwise to the mixture after which precipitate would form in the mixture. The slurry was allowed to stir in the ice-brine bath for 30 minutes. The solid was filtered off and washed with diethyl ether (2 x 10 mL). The collected diazonium salt was dried over vacuum for 5 minutes and collected without further purification.

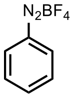

1

Prepared according to General Procedure A with aniline (93 mg, 91 µL, 1.00 mmol). Benzenediazonium **1** was obtained as a white solid (181 mg, 94%). The NMR matched previously reported spectra.[1]

^{1}H NMR (400 MHz, CD_3CN) δ 8.46 (ddd, *J* = 8.8, 2.8, 1.3 Hz, 2H), 8.27 (ddt, *J* = 8.0, 7.4, 1.3 Hz, 1H), 8.03 – 7.88 (m, 2H).

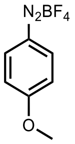

2

Prepared according to General Procedure A with 4-methoxyaniline (123 mg, 1.00 mmol). 4-methoxy-benzene-1-diazonium **2** was obtained as a white solid (186 mg, 84%). The NMR matched previously reported spectra.[1]

^{1}H NMR (400 MHz, CD_3CN) δ 8.42 – 8.36 (m, 2H), 7.42 – 7.28 (m, 2H), 4.06 (s, 3H).

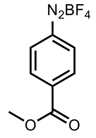

3

Prepared according to General Procedure A with methyl 4-aminobenzoate (151 mg, 1.00 mmol). Methyl 4-benzoate-1-diazonium **3** was obtained as a white solid (200 mg, 0.800 mmol, 80%). The NMR matched previously reported spectra.[2]

^{1}H NMR (400 MHz, CD_3CN) δ 8.60 (dq, *J* = 8.9, 1.9 Hz, 2H), 8.46 – 8.37 (m, 2H), 3.98 (s, 3H).

^{13}C NMR (101 MHz, CD_3CN) δ 164.63, 142.13, 133.73, 132.81, 119.35, 54.16

HRMS: m/z (ESI) Calcd for $C_8H_7N_2O_2$ $[M]^+$: 163.0502, found: 163.0492.

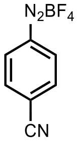

4

Prepared according to General Procedure A with 4-aminobenzonitrile (118 mg, 1.00 mmol). 4-nitrile-benzene-1-diazonium **4** was obtained as a white solid (178 mg, 82%). The NMR matched previously reported spectra.[3]

^{1}H NMR (400 MHz, CD_3CN) δ 8.61 (dt, *J* = 9.0, 1.2 Hz, 2H), 8.27 – 8.22 (m, 2H).

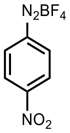

5

Prepared according to General Procedure A with 4-nitroaniline (138 mg, 1.00 mmol). 4-nitro-1-benzene-diazonium **5** was obtained as a white solid (193 mg, 0.815 mmol, 82%). The NMR matched previously reported spectra.[1]

^{1}H NMR (400 MHz, CD_3CN) δ 8.76 – 8.70 (m, 2H), 8.65 – 8.59 (m, 2H).

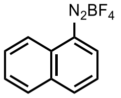

6

Prepared according to General Procedure A with 1-naphthylamine (143 mg, 1.00 mmol). Naphthalene-1-diazonium **6** was obtained as a purple solid (168 mg, 0.694 mmol, 69%). The NMR matched previously reported spectra.[4]

^{1}H NMR (400 MHz, CD_3CN) δ 8.93 (dt, *J* = 7.9, 1.2 Hz, 1H), 8.89 – 8.84 (m, 1H), 8.37 (d, *J* = 8.3 Hz, 1H), 8.26 (dt, *J* = 8.4, 0.9 Hz, 1H), 8.11 (ddd, *J* = 8.4, 7.0, 1.2 Hz, 1H), 8.01 – 7.93 (m, 2H).

7

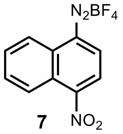

Prepared according to General Procedure A with 4-nitro-1-naphthylamine (188 mg, 1.00 mmol). 4-nitro-naphthalene-1-diazonium **7** was obtained as a yellow solid (228 mg, 0.794 mmol, 79%).

^{1}H NMR (400 MHz, CD_3CN) δ 9.10 (d, *J* = 8.5 Hz, 1H), 8.48 (dt, *J* = 8.7, 0.9 Hz, 1H), 8.45 – 8.37 (m, 2H), 8.24 (ddd, *J* = 8.4, 7.1, 1.1 Hz, 1H), 8.15 (ddd, *J* = 8.4, 7.1, 1.1 Hz, 1H).

^{13}C NMR (126 MHz, CD_3CN) δ 155.71, 138.05, 134.84, 133.82, 130.33, 125.91, 125.59, 123.27, 123.08, 115.52. ^{19}F NMR (471 MHz, CD_3CN) δ -151.54.

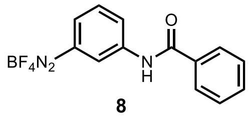

8

Prepared according to General Procedure A with *N*-(3-aminobenzene)benzamide **S1** (212 mg, 1.00 mmol). *N*-(3-diazobenzene)benzamide **8** was obtained as a pale orange solid (274 mg, 87%).

^{1}H NMR (400 MHz, CD_3CN) δ 9.44 (s, 1H), 9.36 (t, *J* = 2.2 Hz, 1H), 8.22 (dddt, *J* = 9.2, 8.3, 1.8, 0.9 Hz, 2H), 8.04 – 7.94 (m, 2H), 7.89 (t, *J* = 8.4 Hz, 1H), 7.70 – 7.62 (m, 1H), 7.57 (ddd, *J* = 8.2, 6.5, 1.3 Hz, 2H).

^{13}C NMR (126 MHz, CD_3CN) δ 167.58, 142.48, 134.38, 133.66, 133.50, 133.09, 129.70, 128.68, 128.38, 121.70, 115.84. ^{19}F NMR (471 MHz, CD_3CN) δ -151.61.

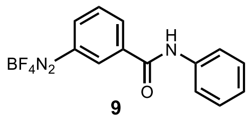

9

Prepared according to General Procedure A with N-phenyl 3-aminobenzamide **S2** (212 mg, 1.00 mmol). *N*-phenyl-3-diazobenzamide **9** was obtained as a pale yellow solid (210 mg, 68%).

^{1}H NMR (400 MHz, CD_3CN) δ 9.10 (s, 1H), 8.98 (t, *J* = 1.9 Hz, 1H), 8.70 (ddd, *J* = 7.9, 1.7, 1.0 Hz, 1H), 8.61 (ddd, *J* = 8.4, 2.2, 1.0 Hz, 1H), 8.08 (t, *J* = 8.2 Hz, 1H), 7.76 – 7.68 (m, 2H), 7.43 (dd, *J* = 8.5, 7.4 Hz, 2H), 7.30 – 7.18 (m, 1H).

^{13}C NMR (101 MHz, CD_3CN) δ 162.27, 140.82, 139.37, 138.84, 135.20, 133.06, 132.76, 129.91, 125.96, 121.61, 116.62.

3. General Procedure B for Synthesis of Multidiazonium Salts

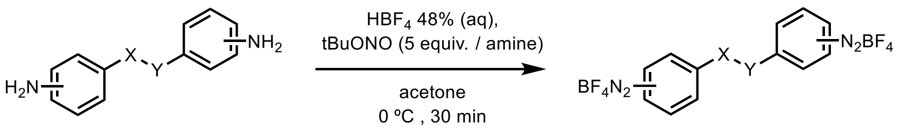

In a 20 mL glass vial, 0.200-1.00 mmol of corresponding amine was suspended in a mixture of 2 mL acetone and 1 mL of a 48% aqueous solution of HBF_4. The vial was placed in an ice-brine bath and tert-butyl nitrite (6 equiv. per amine) was added dropwise to the mixture after which precipitate would form in the mixture. The slurry was allowed to stir in the ice-brine bath for 30 minutes. The solid was filtered off and washed with diethyl ether (2 x 10 mL). The collected diazonium salt was dried over vacuum for 5 minutes and collected without further purification.

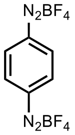

10

Prepared according to General Procedure B with benzene-1,4-diamine (108 mg, 1.00 mmol). Benzene-1,4-bisdiazonium **10** was obtained as a dark yellow solid (202 mg, 67%).

^{1}H NMR (400 MHz, CD_3CN) δ 8.97 (s, 4H).

^{19}F NMR (471 MHz, CD_3CN) δ -151.40.

IR (cm^{-1}): 3105, 2320, 1303, 1028, 860.

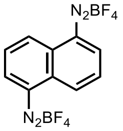

11

Prepared according to General Procedure B with naphthalene-1,5-diamine (158 mg, 1.00 mmol). Naphthalene-1,5-bisdiazonium **11** was obtained as a beige solid (290 mg, 82%).

^{1}H NMR (400 MHz, CD_3CN) δ 9.30 (dd, *J* = 7.9, 0.9 Hz, 2H), 9.09 (d, *J* = 8.6 Hz, 2H), 8.46 (t, *J* = 8.3 Hz, 2H).

^{13}C NMR (126 MHz, CD_3CN) δ 141.90, 137.31, 132.93, 128.75.

^{19}F NMR (471 MHz, CD_3CN) δ -146.20.

IR (cm^{-1}): 3101, 2284, 1505, 1353, 1248, 1203, 1026, 829, 806.

HRMS: m/z (ESI) Calcd for $C_{10}H_6N_4BF_4$ $[M+BF_4]^+$: 269.0616, found: 269.0611.

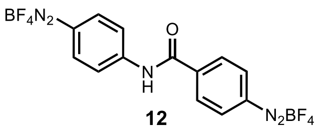

12

Prepared according to General Procedure B with *N*-(4-aminobenzene)-4-aminobenzamide **S4** (227 mg, 1.00 mmol). *N*-(4-diazobenzene)-4-diazobenzamide **12** was obtained as a yellow solid (380 mg, 89%).

^{1}H NMR (400 MHz, CD_3CN) δ 9.98 (s, 1H), 8.74 – 8.58 (m, 2H), 8.52 – 8.42 (m, 2H), 8.41 – 8.31 (m, 2H), 8.29 – 8.20 (m, 2H).

^{13}C NMR (126 MHz, CD_3CN) δ 164.92, 150.89, 145.54, 135.62, 133.86, 131.89, 122.61, 118.95, 106.67.

^{19}F NMR (471 MHz, CD_3CN) δ -151.36.

HRMS: m/z (ESI) Calcd for $C_{13}H_{10}N_5O$ $[M+H]^+$: 252.0874, found: 252.0864.

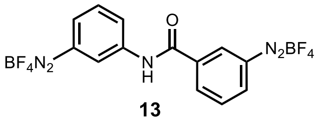

13

Prepared according to General Procedure B with *N*-(3-aminobenzene)-3-aminobenzamide **S3** (227 mg, 1.00 mmol). *N*-(3-diazobenzene)-3-diazobenzamide **13** was obtained as a pale orange solid (330 mg, 77%).

^{1}H NMR (500 MHz, CD_3CN) δ 9.81 (s, 1H), 9.25 (t, *J* = 2.2 Hz, 1H), 9.02 (t, *J* = 2.0 Hz, 1H), 8.73 (dt, *J* = 8.1, 1.4 Hz, 1H), 8.68 (ddd, *J* = 8.4, 2.2, 1.0 Hz, 1H), 8.38 – 8.25 (m, 2H), 8.12 (t, *J* = 8.2 Hz, 1H), 7.95 (t, *J* = 8.4 Hz, 1H).

^{13}C NMR (126 MHz, CD_3CN) δ 163.32, 141.43, 141.10, 137.79, 136.05, 133.78, 133.40, 133.35, 133.02, 129.39, 122.25, 117.02, 116.21.

^{19}F NMR (471 MHz, CD_3CN) δ -151.03.

HRMS: m/z (ESI) Calcd for $C_{13}H_9N_5O$ $[M]^+$: 251.0796, found: 251.0767.

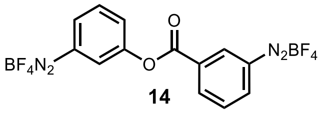

14

Prepared according to General Procedure B with (3-aminophenyl)-3-aminobenzoate **S5** (227 mg, 1.00 mmol). (3-diazophenyl)-3-diazobenzoate **14** was obtained as a beige solid (250 mg, 59%).

^{1}H NMR (400 MHz, CD_3CN) δ 9.25 (t, J = 2.2 Hz, 1H), 8.93 (dt, J = 8.2, 1.4 Hz, 1H), 8.79 (ddd, J = 8.4, 2.2, 1.1 Hz, 1H), 8.56 (t, J = 2.2 Hz, 1H), 8.51 (ddd, J = 8.2, 2.2, 1.1 Hz, 1H), 8.23 (ddd, J = 8.5, 2.2, 1.1 Hz, 1H), 8.17 (t, J = 8.2 Hz, 1H), 8.07 (t, J = 8.4 Hz, 1H).

^{13}C NMR (126 MHz, CD_3CN) δ 161.35, 151.64, 143.29, 137.70, 137.04, 134.73, 134.39, 133.77, 132.87, 131.88, 117.47, 116.62.

^{19}**F** NMR (471 MHz, CD_3CN) δ -151.26.

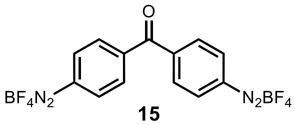

15

Prepared according to General Procedure B with 4,4’-diaminobenzophenone (212 mg, 1.00 mmol). Benzophenone-4,4'-bisdiazonium **15** was obtained as a pale-yellow solid (400 mg, 98%).

^{1}**H NMR** (400 MHz, CD_3CN) δ 8.68 – 8.65 (m, 4H), 8.22 – 8.19 (m, 4H).

^{13}**C NMR** (101 MHz, CD_3CN) δ 191.84, 146.06, 133.88, 133.26, 119.72.

^{19}**F NMR** (471 MHz, CD_3CN) δ -151.36.

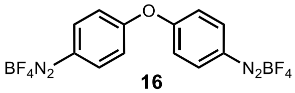

16

Prepared according to General Procedure B with 4,4’-oxydianiline (200 mg, 1.00 mmol). Di(benzene-4-diazonium)ether **16** was obtained as a white solid (370 mg, 93%).

^{1}**H NMR** (400 MHz, CD_3CN) δ 8.63 – 8.55 (m, 1H), 7.65 – 7.57 (m, 1H).

^{13}**C NMR** (101 MHz, CD_3CN) δ 164.98, 137.09, 123.44, 110.25.

^{19}**F NMR** (471 MHz, CD_3CN) δ -151.39.

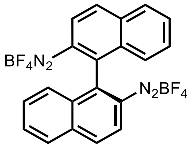

17

Prepared according to General Procedure B with (*R*)-(+)-1,1′-binaphthyl-2,2′-diamine (80.0 mg, 0.281 mmol). (R)-[1,1'-binaphthalene]-2,2'-bisdiazonium **17** was obtained as a yellow solid (93.5 mg, 69%).

^{1}**H NMR** (400 MHz, CD_3CN) δ 8.83 (dd, *J* = 9.2, 0.9 Hz, 2H), 8.66 (d, *J* = 9.2 Hz, 2H), 8.49 (dt, *J* = 8.5, 0.9 Hz, 2H), 8.18 (ddd, *J* = 8.4, 6.9, 1.2 Hz, 2H), 7.90 (ddd, *J* = 8.4, 6.9, 1.2 Hz, 2H), 7.58 (d, *J* = 8.4 Hz, 2H).

^{13}**C NMR** (126 MHz, CD_3CN) δ 140.03, 136.80, 136.76, 135.20, 133.52, 132.22, 131.25, 128.11, 125.28, 114.64.

^{19}**F NMR** (471 MHz, CD_3CN) δ -151.50.

HRMS: m/z (ESI) Calcd for $C_{20}H_{12}N_4BF_4$ $[M+BF_4]^+$: 395.1080, found: 395.1069.

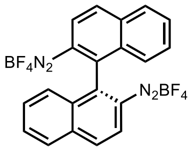

18

Prepared according to General Procedure B with (*S*)-(−)-1,1′-Binaphthyl-2,2′-diamine (80.0 mg, 0.281 mmol). (S)-[1,1'-binaphthalene]-2,2'-bisdiazonium **18** was obtained as a yellow solid (108 mg, 80%).

^{1}H NMR (500 MHz, CD_3CN) δ 8.83 (d, *J* = 9.2 Hz, 2H), 8.66 (dd, *J* = 9.2, 1.2 Hz, 2H), 8.49 (d, *J* = 8.4 Hz, 2H), 8.18 (ddd, *J* = 8.4, 6.9, 1.2 Hz, 2H), 7.90 (ddd, *J* = 8.4, 6.9, 1.2 Hz, 2H), 7.58 (d, *J* = 8.4 Hz, 2H).

^{13}C NMR (126 MHz, CD_3CN) δ 140.05, 136.82, 135.21, 133.52, 132.26, 131.26, 128.14, 125.25, 114.60.

^{19}F NMR (471 MHz, CD_3CN) δ -151.28.

HRMS: m/z (ESI) Calcd for $C_{20}H_{12}N_4BF_4$ $[M+BF_4]^+$: 395.1080, found: 395.1058.

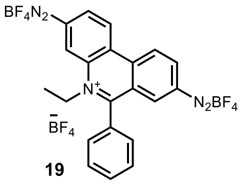

19

Prepared according to General Procedure B with ethidium bromide (98.2 mg, 0.250 mmol). Ethidium bisdiazonium **19** was obtained as a pink solid (127 mg, 85%).

^{1}H NMR (400 MHz, CD_3CN) δ 9.76 (d, *J* = 1.9 Hz, 1H), 9.56 (d, *J* = 9.2 Hz, 1H), 9.48 (d, *J* = 9.2 Hz, 1H), 9.15 (ddd, *J* = 17.5, 9.2, 2.1 Hz, 2H), 8.96 (d, *J* = 2.1 Hz, 1H), 8.00 (t, *J* = 7.6 Hz, 1H), 7.92 (t, *J* = 7.6 Hz, 2H), 7.81 – 7.72 (m, 2H), 4.94 (q, *J* = 7.3 Hz, 2H), 1.65 (t, *J* = 7.3 Hz, 3H).

^{13}C NMR (101 MHz, CD_3CN) δ 170.63, 141.21, 140.51, 136.96, 136.23, 134.16, 134.05, 131.99, 131.94, 131.07, 130.40, 129.56, 128.99, 128.77, 128.61, 121.93, 120.59, 54.76, 14.86.

^{19}F NMR (471 MHz, CD_3CN) δ -151.21.

IR (cm^{-1}): 3095, 2310, 1608, 1582, 1447, 1383, 1339, 1298, 1022, 1002, 892, 829, 766, 698, 601.

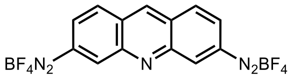

20

Prepared according to General Procedure B with 3,6-diaminoacridine (102 mg, 0.250 mmol). Acridine-3,6-bisdiazonium **20** was obtained as a purple solid (92.3 mg, 91%).

^{1}H NMR (400 MHz, CD_3CN) δ 9.70 (dt, *J* = 1.9, 0.8 Hz, 2H), 9.62 (s, 1H), 8.74 (d, *J* = 9.4 Hz, 2H), 8.38 (dd, *J* = 9.3, 2.1 Hz, 2H).

^{13}C NMR (101 MHz, CD_3CN) δ 148.32, 142.29, 142.01, 135.27, 133.54, 123.27, 119.94.

^{19}F NMR (471 MHz, CD_3CN) δ -151.13.

IR (cm^{-1}): 3100, 2290, 1601, 1559, 1292, 1036, 1012, 930, 832, 804, 768, 740, 682, 636, 608.

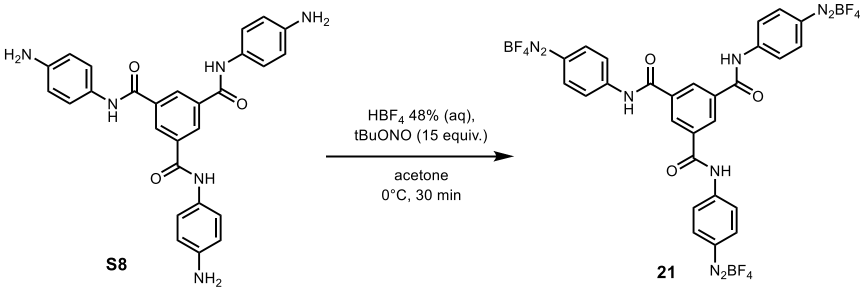

The starting amine was first prepared according to General Procedure 1 with *N*-Boc-1,4-phenylenediamine and benzene-1,3,5-tricarboxylic acid. Instead of an Iron-Acid reduction, 200 mg (0.25 mmol) of coupled product was treated with 2 mL of TFA and allowed to stir for 30 minutes. The product was crashed out with diethyl ether, filtered by vacuum filtration, and washed with ether to obtain *N1,N3,N5*-tris(4-aminobenzene)benzene-1,3,5-tricarboxamide **S8** (120 mg, quant.). Using amine **S8** under general Procedure B, *N1,N3,N5*-tris(benzene-4-diazonium)benzene-1,3,5-tricarboxamide **21** was obtained as a dark brown solid (140 mg, 72%). The NMR spectra show minor impurity. The compound was used without further purification.

^{1}H NMR (400 MHz, CD_3CN) δ 10.02 (s, 3H), 8.82 (s, 3H), 8.52 – 8.40 (m, 6H), 8.39 – 8.27 (m, 6H). ^{19}F NMR (471 MHz, CD_3CN) δ -151.32.

^{13}C NMR (101 MHz, CD_3CN) δ 165.43, 151.04, 135.16, 135.04, 131.94, 121.90, 105.09.

^{19}F NMR (471 MHz, CD_3CN) δ -151.32.

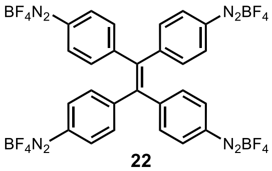

22

Prepared according to General Procedure B with 4,4',4'',4'''-(ethene-1,1,2,2-tetrayl)tetraaniline (118 mg, 0.200 mmol). Tetrakis(benzene-4-diazonium)ethylene **22** was obtained as a yellow solid (145 mg, 92%).

^{1}H NMR (400 MHz, CD_3CN) δ 8.42 – 8.31 (m, 8H), 7.71 – 7.54 (m, 8H).

^{13}C NMR (126 MHz, CD_3CN) δ 151.91, 143.44, 134.91, 133.74, 115.72.

^{19}F NMR (471 MHz, CD_3CN) δ -151.15.

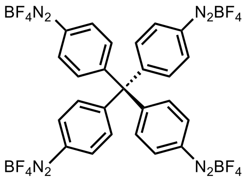

23

Prepared according to General Procedure B with tetrakis(4-aminobenzene)methane (80.0 mg, 0.210 mmol). Tetrakis(benzene-4-diazonium)methane **23** was obtained as a pale beige solid (140 mg, 86%).

^{1}H NMR (400 MHz, CD_3CN) δ 8.52 (d, *J* = 9.1 Hz, 8H), 7.87 (d, *J* = 9.2 Hz, 8H).

^{13}C NMR (126 MHz, CD_3CN) δ 154.96, 134.06, 133.97, 115.26, 99.29.

^{19}F NMR (471 MHz, CD_3CN) δ -151.31.

HRMS: m/z (ESI) Calcd for $C_{25}H_{16}N_8NaB_4F_{16}$ $[M+Na+B_4F_{16}]^+$: 799.1523, found: 799.1527.

IR (cm^{-1}): 3112, 2284, 1574, 1422, 1291, 1030, 827, 757, 553.

4. General Procedure C for Synthesis of Glycosyl Diazonium Precursors

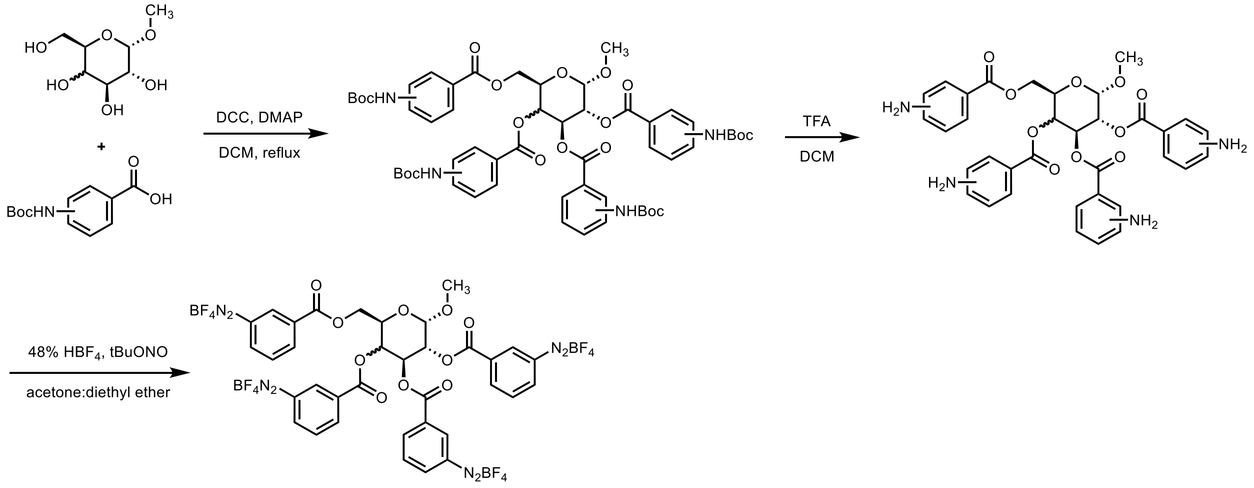

In a round bottom flask, the corresponding Boc-protected benzoic acid derivative (1.1 equiv. / hydroxyl), DMAP (1.0 equiv.) and DCC (DCC = dicyclohexylcarbodiimide) (1.1 equiv. / hydroxyl) were added to a solution of the corresponding sugar in DCM (0.1 M). The flask was sealed with a reflux condenser, purged with nitrogen, and allowed to stir at reflux for 16 hours. The mixture was allowed to cool to room temperature and an equivalent volume amount of diethyl ether was added. The solution was filtered through celite. The filtrate was concentrated and purified by flash column chromatography. The corresponding coupled product was deprotected by dissolving the sugar in DCM (0.5 M) followed by the addition of an equivalent volume of trifluoroacetic acid. The solution was stirred for 2 hours, neutralized with sodium bicarbonate (white precipitate observed) and extracted in DCM and water. The organic layer was concentrated to afford the product, which was used without further purification. In a 20 mL glass vial, 0.100-1.00 mmol of corresponding amine was suspended in a mixture of 1 mL acetone, 1 mL diethyl ether, and 1 mL of a 48% aqueous solution of HBF_4. The vial was placed in an ice-brine bath and tert-butyl nitrite (6 equiv. per amine) was added dropwise, after which precipitate would form in the mixture. The slurry was allowed to stir in the ice-brine bath for 30 minutes. The solid was filtered off and washed with diethyl ether (2 x 10 mL). The collected diazonium salt was dried over vacuum for 5 minutes and collected without further purification.

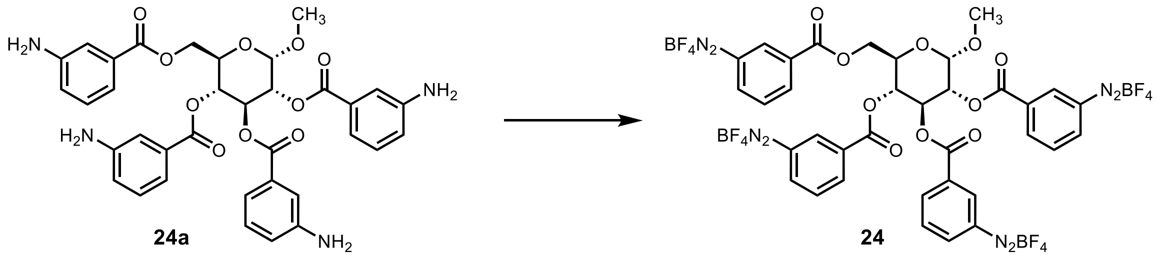

Prepared according to General Procedure C with methyl-α-D-glucopyranoside as the starting glycoside and Boc-3-aminobenzoic acid. Using Glycoside **24a** (72 mg, 0.10 mmol), diazonium salt **24** was obtained as a white solid (97 mg, 91%). The NMR spectra show minor impurity. The compound was used without further purification.

^{1}H NMR (400 MHz, CD_3CN) δ 9.12 – 9.10 (m, 1H), 9.00 – 8.90 (m, 3H), 8.82 (dt, J = 8.2, 1.4 Hz, 1H), 8.76 – 8.55 (m, 7H), 8.11 (t, J = 8.2 Hz, 1H), 8.06 – 7.91 (m, 3H), 6.05 (dd, J = 10.0, 9.1 Hz, 1H), 5.84 (dd, J = 10.0, 9.1 Hz, 1H), 5.54 (dd, J = 10.0, 3.6 Hz, 1H), 5.29 (d, J = 3.6 Hz, 1H), 4.71 – 4.61 (m, 2H), 4.56 (dt, J = 10.0, 3.0 Hz, 1H), 3.54 (s, 3H).

^{13}C NMR (101 MHz, CD_3CN) δ 163.06, 163.01, 162.61, 162.47, 142.87, 142.87, 142.77, 142.65, 137.09, 136.98, 134.57, 134.11, 134.05, 133.55, 133.38, 133.36, 133.23, 117.11, 97.40, 73.46, 73.24, 71.03, 67.64, 64.63, 56.43.

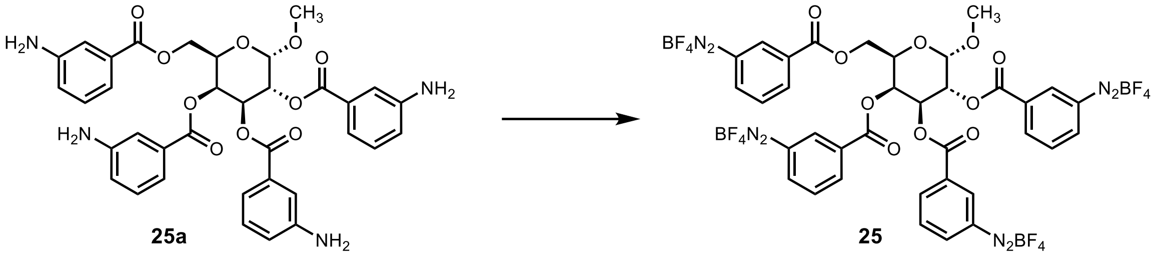

Prepared according to General Procedure C with methyl-α-D-galactopyranoside as the starting glycoside and Boc-3-aminobenzoic acid. Using Glycoside **25a** (100 mg, 0.15 mmol), diazonium salt **25** was obtained as a white solid (145 mg, 90%).

^{1}H NMR (400 MHz, CD_3CN) δ 9.12 (t, J = 1.9 Hz, 1H), 9.00 (t, J = 1.9 Hz, 1H), 8.96 (t, J = 1.9 Hz, 1H), 8.87 (t, J = 1.9 Hz, 1H), 8.78 – 8.55 (m, 8H), 8.10 – 7.92 (m, 4H), 6.05 (dd, J = 3.5, 1.2 Hz, 1H), 5.94 (dd, J = 10.7, 3.5 Hz, 1H), 5.72 (dd, J = 10.7, 3.6 Hz, 1H), 5.43 (d, J = 3.6 Hz, 1H), 4.78 – 4.56 (m, 3H), 3.53 (s, 3H).

^{13}C NMR (101 MHz, CD_3CN) δ 163.18, 162.88, 162.68, 162.55, 143.08, 142.77, 142.56, 137.24, 136.98, 136.93, 134.79, 134.16, 134.14, 134.01, 133.91, 133.55, 133.51, 133.41, 133.31, 133.23, 133.11, 117.10, 117.07, 117.05, 97.78, 71.92, 71.30, 70.71, 67.02, 64.64, 56.42.

^{19}F NMR (471 MHz, CD_3CN) δ -151.10.

IR (cm^{-1}): 3096, 2359, 2293, 1735, 1599, 1568, 1433, 1266, 1031, 966, 813, 746, 651, 541.

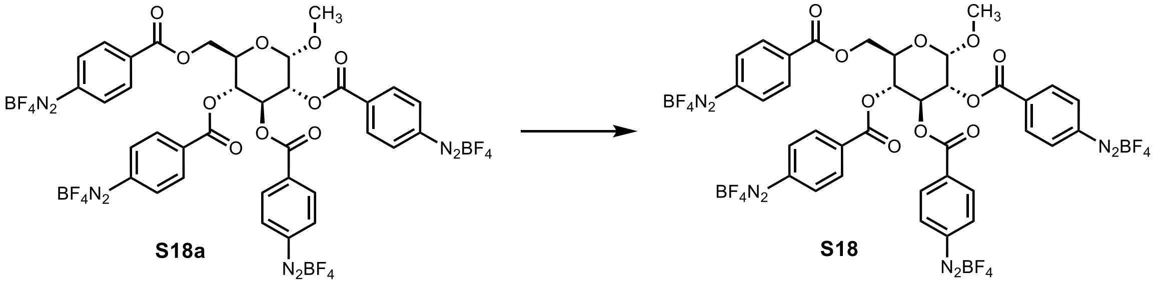

Prepared according to General Procedure C with methyl-α-D-glucopyranoside as the starting glycoside and Boc-4-aminobenzoic acid. Using Glycoside **S18a** (91 mg, 0.14 mmol), diazonium salt **S18** was obtained as a white solid (125 mg, 84%).

^{1}H NMR (400 MHz, CD_3CN) δ 8.64 – 8.45 (m, 10H), 8.37 – 8.22 (m, 6H), 6.07 (t, J = 9.6 Hz, 1H), 5.83 (t, J = 9.6 Hz, 1H), 5.51 (dd, J = 10.0, 3.6 Hz, 1H), 5.27 (d, J = 3.6 Hz, 1H), 4.72 – 4.52 (m, 3H), 3.53 (s, 3H).

^{13}C NMR (101 MHz, CD_3CN) δ 163.83, 163.78, 163.39, 163.33, 141.45, 141.45, 141.04, 140.93, 140.72, 133.78, 133.78, 133.67, 133.64, 133.12, 133.00, 132.97, 132.94, 120.09, 119.97, 119.92, 119.83, 97.41, 73.57, 73.35, 71.40, 67.58, 65.04, 56.39.

IR (cm^{-1}): 3113, 2294, 1732, 1603, 1508, 1417, 1265, 1034, 1011, 915, 855, 755, 687, 666, 578.

5. Additional Diazonium Substrates

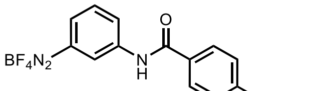

S9

Prepared according to General Procedure B with N-(3-aminobenzene)-4-aminobenzamide **S6** (90 mg, 0.396 mmol). N-(3-diazobenzene) 4-diazobenzamide **S9** was obtained as a yellow solid (154 mg, 92%).

^{1}H NMR (500 MHz, CD_3CN) δ 9.80 (s, 1H), 9.25 (t, *J* = 2.2 Hz, 1H), 8.68 – 8.62 (m, 2H), 8.38 – 8.35 (m, 2H), 8.31 – 8.24 (m, 2H), 7.94 (t, *J* = 8.4 Hz, 1H).

^{13}C NMR (126 MHz, CD_3CN) δ 164.53, 145.76, 141.40, 133.85, 133.81, 133.42, 131.75, 129.47, 122.24, 116.22.

^{19}F NMR (471 MHz, CD_3CN) δ -151.44.

IR (cm^{-1}): 3351, 2303, 2271, 1704, 1605, 1574, 1532, 1483, 1417, 1335, 1310, 1262, 1037, 1003, 994, 950, 915, 888, 853, 833, 818, 768, 739, 673.

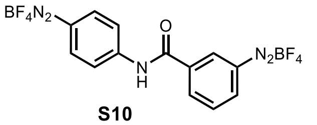

S10

Prepared according to General Procedure B with N-(4-aminobenzene)-3-aminobenzamide **S7** (80.0 mg, 0.352 mmol). N-(4-diazobenzene) 3-diazobenzamide **S10** was obtained as a yellow solid (116 mg, 78%).

^{1}H NMR (400 MHz, CD_3CN) δ 10.05 (s, 1H), 9.00 (t, *J* = 1.9 Hz, 1H), 8.71 (dddd, *J* = 13.3, 8.4, 1.9, 1.0 Hz, 2H), 8.54 – 8.43 (m, 2H), 8.34 – 8.22 (m, 2H), 8.13 (t, *J* = 8.2 Hz, 1H).

^{13}C NMR (126 MHz, CD_3CN) δ 163.69, 150.96, 141.31, 137.69, 136.26, 135.60, 133.39, 133.13, 122.59, 117.08, 106.54.

^{19}F NMR (471 MHz, CD_3CN) δ -151.25.

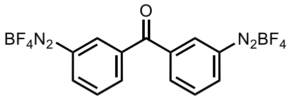

S11

Prepared according to General Procedure B with 3,3'-diaminobenzophenone (80.0 mg, 0.377 mmol). Benzophenone-3,3'-bisdiazonium **S11** was obtained as a pale-yellow solid (109 mg, 71%).

^{1}H NMR (400 MHz, CD_3CN) δ 8.85 (t, *J* = 1.9 Hz, 2H), 8.77 (ddd, *J* = 8.4, 2.1, 1.1 Hz, 2H), 8.61 (ddd, *J* = 8.0, 1.7, 1.1 Hz, 2H), 8.15 (t, *J* = 8.2 Hz, 2H).

^{13}C NMR (101 MHz, CD_3CN) δ 188.60, 143.12, 138.72, 136.94, 134.50, 133.61, 117.11.

^{19}F NMR (471 MHz, CD_3CN) δ -151.25.

HRMS: m/z (ESI) Calcd for $C_{13}H_8N_4OBF_4$ $[M+BF_4]^+$: 323.0716, found: 323.0693.

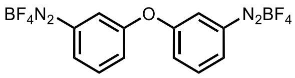

S12

Prepared according to General Procedure B with 3,3'-oxydianiline (80.0 mg, 0.400 mmol). Di(benzene-3-diazonium)ether **S12** was obtained as a pale yellow solid (129 mg, 81%).

^{1}H NMR (400 MHz, CD_3CN) δ 8.40 (dt, J = 6.8, 2.1 Hz, 2H), 8.22 – 8.15 (m, 2H), 8.06 – 7.96 (m, 4H).

^{13}C NMR (101 MHz, CD_3CN) δ 156.85, 134.88, 134.37, 130.22, 122.61, 117.02.

^{19}F NMR (471 MHz, CD_3CN) δ -151.30.

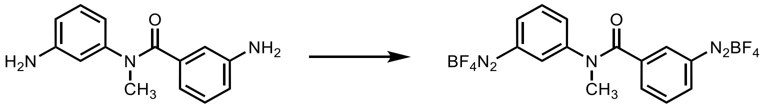

S13 **S14**

During the synthesis of **S3** under General Procedure 1, some of the coupled nitro product (1.0 g, 3.5 mmol, 1 equiv.) was methylated with iodomethane (Under inert conditions, 1.25 equiv. of 60% NaH in 0.2 M THF, stirring at room temperature for 1 hour, then addition of 4 equiv. of iodomethane and stirring at room temperature overnight, followed by flash column chromatography 4:1 hexanes:ethylacetate) and then continued on with the reduction of the methylated nitroarene to yield *N*-(3-aminobenzene)-*N*-methyl-3-aminobenzamide **S13.** The final diazonium salt was prepared according to General Procedure B with **S13** (241 mg, 1.00 mmol). *N*-(3-diazobenzene)-*N*-methyl-3-diazobenzamide **S14** was obtained as a beige solid (310 mg, 71%).

^{1}H NMR (400 MHz, CD_3CN) δ 8.51 – 8.46 (m, 2H), 8.41 (t, J = 2.2 Hz, 1H), 8.34 (ddd, J = 8.4, 2.1, 1.0 Hz, 1H), 8.22 – 8.15 (m, 1H), 8.14 – 8.08 (m, 1H), 7.93 – 7.83 (m, 2H), 3.45 (s, 3H).

^{13}C NMR (126 MHz, CD_3CN) δ 166.38, 146.15, 141.85, 141.01, 139.17, 134.57, 133.77, 133.16, 132.64, 131.55, 130.44, 116.55, 38.97.

^{19}F NMR (471 MHz, CD_3CN) δ -151.22.

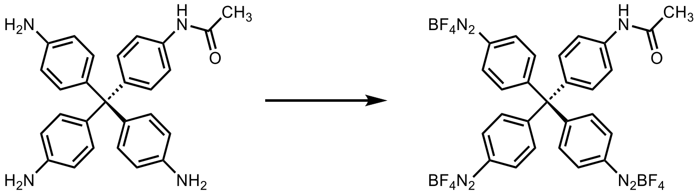

S15 **S16**

In a round bottom flask, 720 mg tetrakis(4-aminobenzene)methane (1.9 mmol, 1 equiv.) was dissolved in 100 mL dry THF. Then, 0.6 mL of diisopropylethylamine (2 equiv.) was added and the flask was placed in

an ice-brine bath. A 10 mL 0.19 M solution of acetyl chloride in dry THF was prepared (1 equiv.) and added via a syringe pump (1 mL / 1 min) to the flask. The flask was then removed from the bath and allowed to stir at room temperature for 1 hour (TLC analysis confirmed presence of monoacetylated spot). The mixture was concentrated by solvent evaporation and purified by flash column chromatograph (0 to 5% MeOH in DCM) to achieve **S15** as a white solid (180 mg, 23%). The final diazonium was prepared according to General Procedure B with **S15** (80.0 mg, 0.210 mmol). Diazonium **S16** was obtained as a white solid (140 mg, 86%).

^{1}H NMR (400 MHz, CD_3CN) δ 8.52 (s, 1H), 8.50 – 8.43 (m, 6H), 7.93 – 7.85 (m, 6H), 7.66 – 7.57 (m, 2H), 7.24 – 7.12 (m, 2H), 2.06 (s, 3H).

^{13}C NMR (101 MHz, CD_3CN) δ 157.86, 134.28, 134.01, 131.72, 120.85, 114.41, 68.40, 24.25.

IV. Examination of DNA-Cleaving Ability of Diazonium Salts

Before the reaction, a fresh 500 μM stock solution of the diazonium salt was prepared by dissolving 10 μmol of the salt in 20 mL of ddH$_2$O. In 1.5 mL Eppendorf tubes, a serial dilution was performed to achieve a concentration range between 1000 nM and 1 nM solutions of the diazonium salt in ddH$_2$O. In a standard reaction in 0.2 mL PCR tubes, 1 μL (0.5 μg) of aqueous supercoiled pBR322 plasmid DNA (0.5 μg/μL aqueous stock) and 20 μL of the corresponding diazonium solutions were mixed. The samples were incubated at 25°C in between two Kessil pro160 - 525 nm green LED lights (44 W) for 1.5 hours. The DNA-cleaving properties of the diazonium salts were then examined by agarose gel electrophoresis. The cleavage assays were performed in duplicates. *As a control within every assay, a sample of DNA with 20 μL of ddH$_2$O in the absence of diazonium was also irradiated along with the rest of the samples and used as an internal standard for the starting percentage of supercoiled plasmid and ensured any cleavage observed was not from irradiation or other factors during the reaction.*

Note: For monodiazonium salts, the serial dilution from the stock solution was performed to achieve a concentration range of 4,000 nM, 2,000 nM, 1,000 nM, 500 nM, 400 nM, 200 nM, 100 nM and 50 nM instead. For benzenediazonium **1**, the serial dilution from the stock solution was performed to achieve 80 μM, 40 μM, 30 μM, 20 μM, 10 μM, 4 μM, and 0.5 μM instead. Similarly, for the 4-methoxy-benzene-1-diazonium **5**, the serial dilution from the stock solution was performed to achieve 250 μM, 100 μM, 80 μM, 60 μM, 40 μM, 10 μM, and 5 μM instead.

V. Agarose Gel Electrophoresis

The agarose gel was prepared by heating a suspension of 0.08% - 1 % (w/v) agarose in 1X Tris-acetate-EDTA (TAE) buffer in a microwave until fully dissolved. GelRed® 10,000X (Biotium) was added to the solution and the stained molten suspension was then poured into a cast and allowed to solidify at room temperature for 45-60 minutes. DNA reaction samples were mixed with 4 μL of Gel Loading Dye Purple 6X (New England Biolabs) and loaded into the wells of the agarose gel. The gel was run at 90 V in TAE buffer for 2 hours at room temperature. After electrophoresis, the gel was analyzed using a UV tray in a Bio-Rad ChemiDoc Imaging System. The different DNA bands were quantified with Bio-Rad Image Lab and plotted against the corresponding diazonium salt concentration in a dose-response plot to generate the EC_{50} value.

VI. DNA Cleavage Assay Controls and Restriction Enzyme Calibrations

Nb.BtsI (Nicking) and EcoRV-HF (Linearizing) Digestion of pBR322: Into two 1.5 mL Eppendorf tubes was added 0.5 μg of pBR322 (1 μL from stock of 0.5 μg/μL in each tube), 16 μL of ddH$_2$O (to reach total 20 μL reaction volume), and 2 μL buffer (10X CutSmart Buffer, NEB Labs). In one tube was added 1 μL Nb.BtsI (10,000 units/mL, NEB Labs) and in the second tube added 1 μL EcoRV-HF (100,000 units/mL, NEB Labs). The tubes were placed in a 37 °C incubator and allowed to incubate for 2 hours. The DNA was then examined by the standard agarose gel electrophoresis (Figure S1).

1,5-diaminonaphthalene and diazonium **11** digestion of pBR322: Into two 0.2 mL PCR tubes was added 0.5 μg of pBR322 (1 μL from stock of 0.5 μg/μL in each tube). Into one of the PCR tubes was added 20 μL of a 500 nM working solution of diazonium **11** in ddH$_2$O (prepared from fresh 500 μM stock solution in ddH$_2$O) and into the other PCR tube was added 20 μL of a 500 nM working solution of the corresponding amine precursor, 1,5-diaminonaphthalene, in ddH$_2$O (prepared from a fresh 500 μM stock solution in DMSO). Into a third PCR tube was added 0.5 μg of pBR322 (1 μL from stock of 0.5 μg/μL in each tube) and 20 μL

ddH$_2$O in the absence of any additional compound. The samples were incubated together at 25°C in between two Kessil pro160 - 525 nm green LED lights (44 W) for 1.5 hours. The DNA-cleavage of the diazonium, amine, and water controls were then examined by the standard agarose gel electrophoresis (Figure S1).

The cleavage pattern of pBR322 (Form I) in the presence of diazonium **11** (500 nM) lead to two new bands (Lane 2), which are assigned as the nicked (Form II) and linear (Form III) strands. These two bands were confirmed as such by their alignment in the same gel with the matching bands formed by pBR322 incubated with the known nicking plasmid, Nb.BtsI, leading to nicked DNA (Lane 1) and pBR322 incubated with restriction enzyme EcoRV-HF, leading to linear DNA (Lane 3), respectively. Additionally, when pBR322 was irradiated with the same concentration (500 nM) of 1,5-diaminonaphthalene, the amine precursor of the diazonium, or with water alone no cleavage was observed (Lanes 4 and 5, respectively). This confirmed that in instances when pBR322 cleavage is observed, it is not from light irradiation nor the general presence of an organic small molecule.

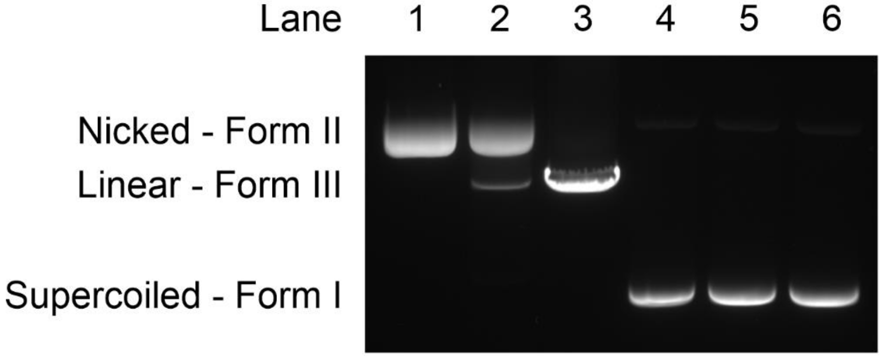

Figure S1. Gel Image of DNA Cleavage Assay Controls with Restriction Enzymes and Amine precursor. Lane 1: pBR322 incubated with nicking enzyme Nb.BtsI; Lane 2: pBR322 irradiated in the presence of 500 nM of diazonium **11**; Lane 3: pBR322 incubated with restriction enzyme EcoRV-HF; Lane 4: pBR322 irradiated in the presence of 500 nM of 1,5-diaminonaphthalene; Lane 5: pBR322 irradiated with water alone; Lane 6: pBR322 with no irradiation.

VII. DNA Cleavage by Irradiation with Different Wavelengths and in the Dark

Experimental Details for Cleavage Assay of Diazonium in Blue, Red, and Ambient light: Similar to the standard assay, a fresh 500 µM stock solution of diazonium salt **23** was prepared by dissolving 10 µmol (7.8 mg) of the salt in 20 mL of ddH_2O. In 1.5 mL Eppendorf tubes, a serial dilution was performed to achieve a concentration range between 1000 nM and 1 nM solutions of the diazonium salt in ddH_2O. In 0.2 mL PCR tubes, 1 µL (0.5 µg) of aqueous supercoiled pBR322 plasmid DNA (0.5 µg/µl aqueous stock) and 20 µL of the corresponding diazonium solutions were mixed. The samples were incubated at 25 °C in between either two Kessil pro160 - 467 nm blue LED lights (34 W), two Kessil pro160 - 660 nm red LED lights (35W), or with ambient light for 1.5 hours (For ambient light reactions, the tubes were left on the bench countertop). The DNA-cleaving properties of the diazonium salts were then examined by the standard agarose gel electrophoresis procedure (Figures S2-S7). The cleavage assays were performed in duplicates.

Experimental Details for the Cleavage Assay of Diazonium in the Dark: Similar to the standard assay, a fresh 500 µM stock solution of diazonium salt **23** was prepared by dissolving 10 µmol (7.8 mg) of the salt in 20 mL of ddH_2O. In a Dark Room, a serial dilution in 1.5 mL Eppendorf tubes was performed to achieve a range between 50,000 nM and 2.5 nM solutions of the diazonium salt in ddH_2O. In 0.2 mL PCR tubes, 1 µL (0.5 µg) of aqueous supercoiled pBR322 plasmid DNA (0.5 µg/µl aqueous stock) and 20 µL of the corresponding diazonium solutions were mixed. The samples were incubated at room temperature wrapped in aluminum foil in the dark room for 1.5 hours. The DNA-cleaving properties of the diazonium salts were then examined by the standard agarose gel electrophoresis procedure (Figure S8-S9). The cleavage assay was performed in duplicates.

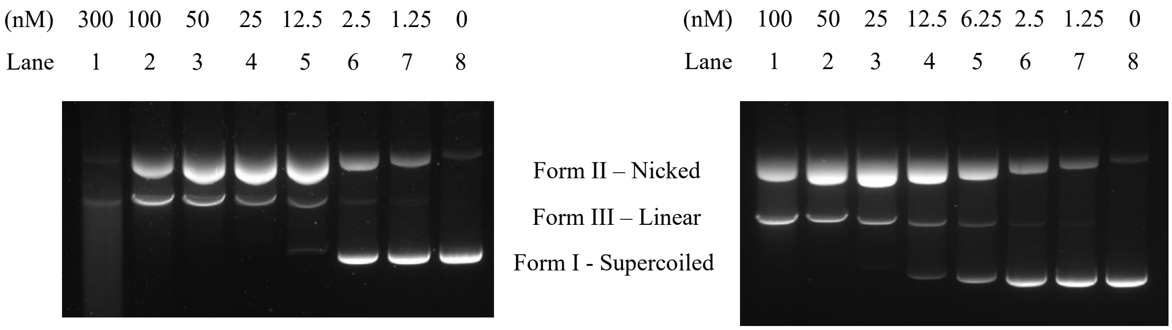

Figure S2. Agarose Gel Images Using Diazonium **23** with 467 nm Blue Light

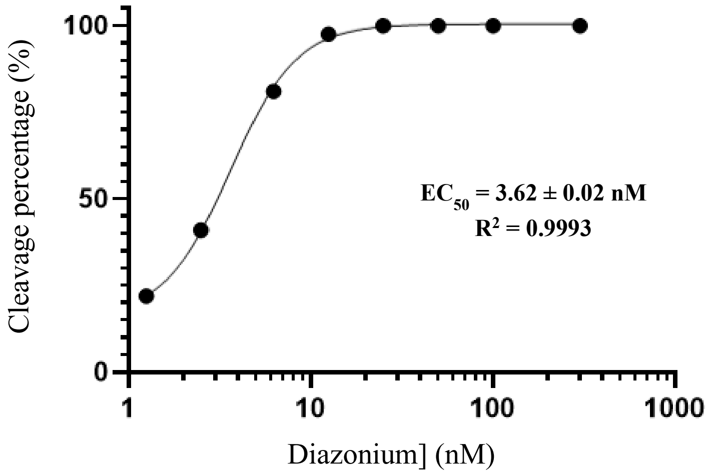

Figure S3. Dose Response Plot of Cleaved DNA Percentage against Concentration of **23** with 467 nm Blue Light

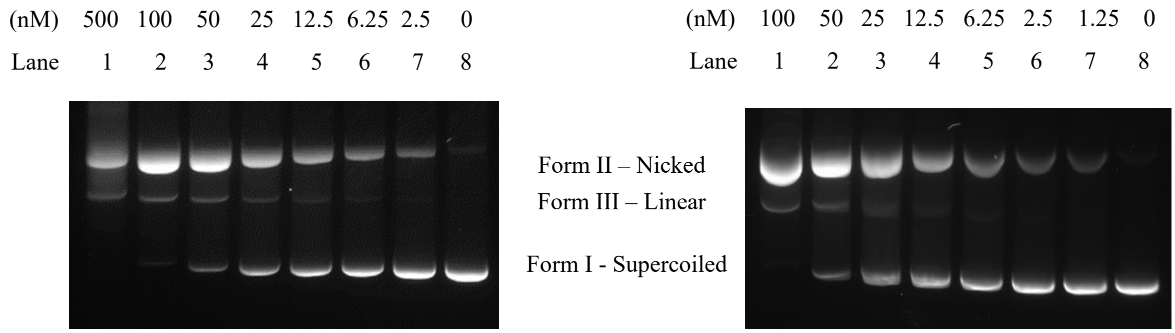

Figure S4. Agarose Gel Images Using Diazonium **23** with 660 nm Red Light

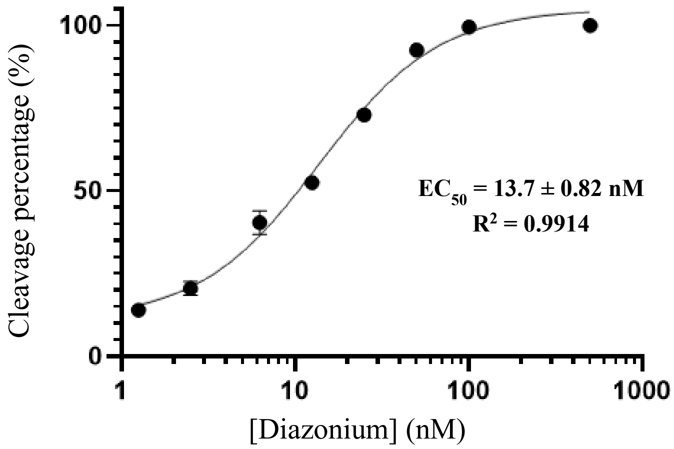

Figure S5. Dose Response Plot of Cleaved DNA Percentage against Concentration of **23** with 660 nm Red Light

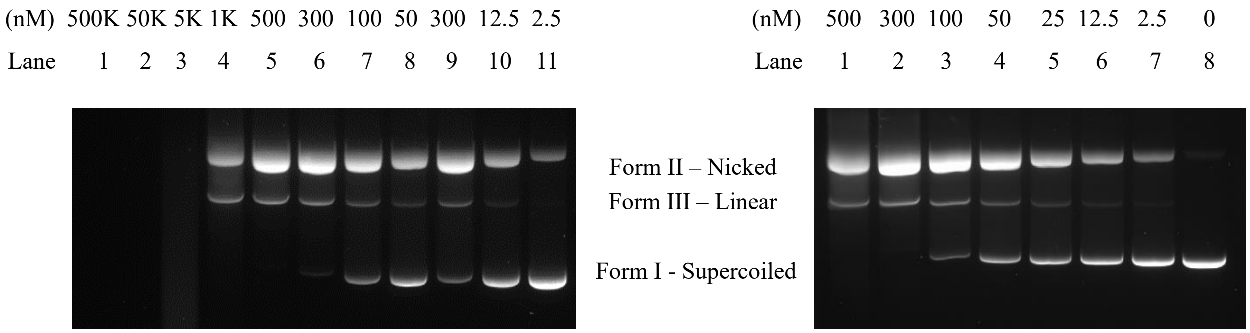

Figure S6. Agarose Gel Images Using Diazonium **23** with Ambient Light

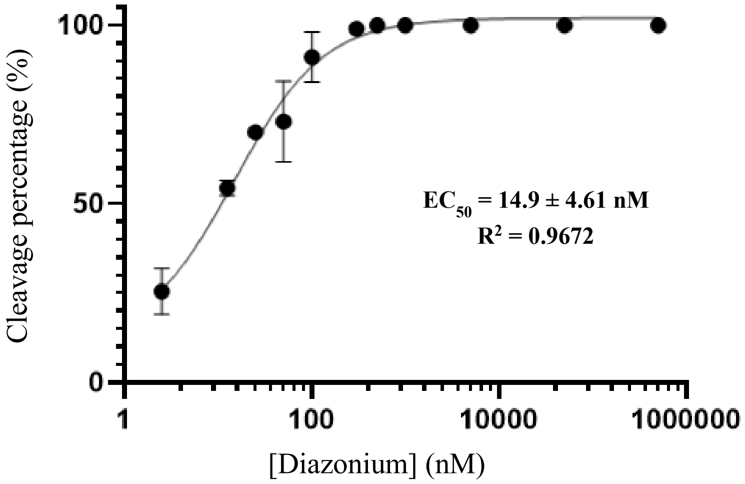

Figure S7. Dose Response Plot of Cleaved DNA Percentage against Concentration of **23** with Ambient Light

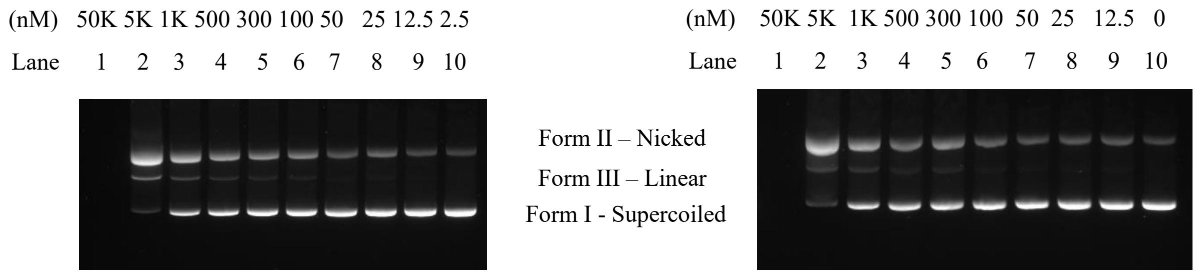

Figure S8. Agarose Gel Images Using Diazonium **23** in the Dark

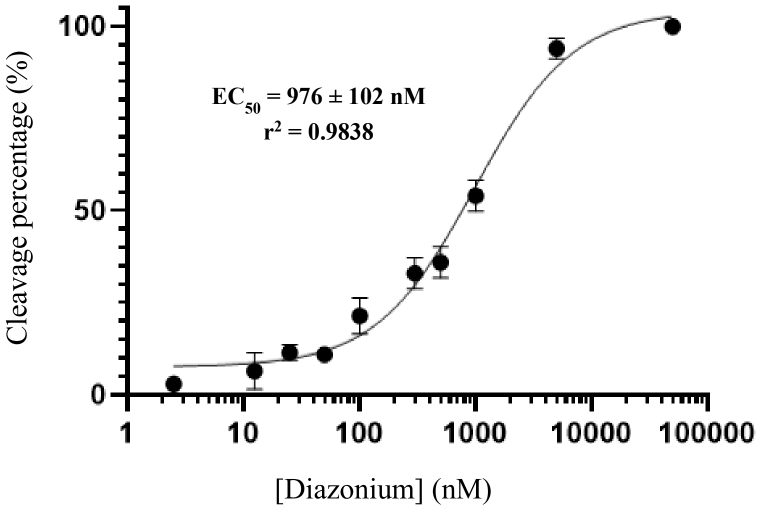

Figure S9. Dose Response Plot of Cleaved DNA Percentage against Concentration of **23** in the Dark

VIII. DNA Cleavage under Deoxygenated Conditions

A sample of double distilled water (ddH_2O) was freeze-pump-thawed (degassed) four times and then brought into an argon-filled glovebox.

The subsequent serial dilution and reaction setup were performed in an argon-filled-glovebox: Similar to the standard assay, a fresh 500 µM stock solution of diazonium salt **23** was prepared by dissolving 2.5 µmol (1.6 mg) of the salt in 4 mL of the deoxygenated ddH_2O. In 1.5 mL Eppendorf tubes, a serial dilution was performed to achieve a range between 500 nM and 1 nM solutions of the diazonium salt in the deoxygenated ddH_2O. In 0.2 mL PCR tubes, 0.5 µL (0.5 µg) of aqueous supercoiled pBR322 plasmid DNA (1 µg/µl stock) and 20 µL of the corresponding diazonium solutions were mixed.

The reaction mixtures were taken out of the glovebox and incubated at 25°C in between two Kessil pro160 - 525 nm green LED lights (44 W) for 1.5 hours. The DNA-cleaving properties of the diazonium salt under deoxygenated conditions were then examined by the standard agarose gel electrophoresis procedure (Figure S10A). A standard reaction with normal ddH2O was setup outside the glovebox and performed alongside as a control. It was also examined by the standard agarose gel electrophoresis procedure (Figure S10B). The dose-response plots were generated for each to obtain their respective EC_{50} values (Figure 2B). The EC_{50} value using deoxygenated ddH_2O was 172 nM, more than a 2-log scale higher than the EC_{50} value of the control using normal ddH_2O, which was 1.64 nM.

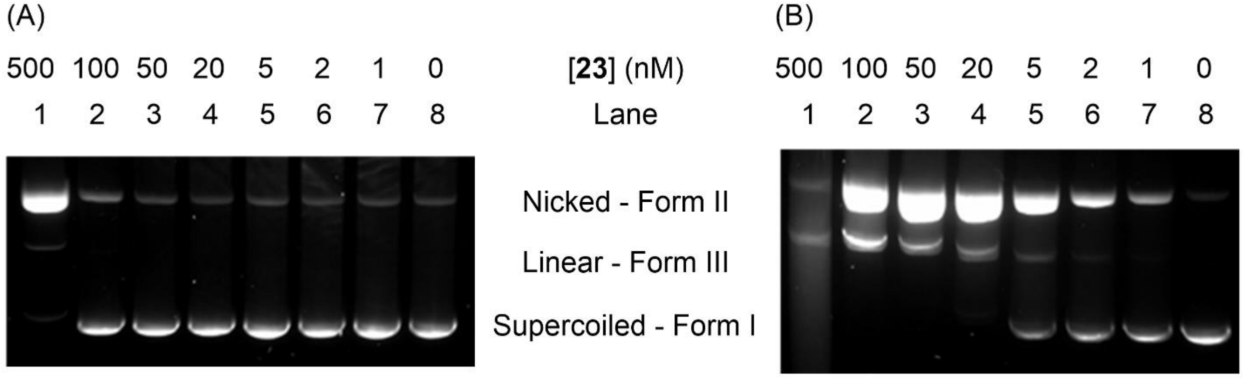

Figure S10. (A) Agarose Gel Images using deoxygenated water and (B) using standard water.

IX. Cleavage Profile of Isolated Linearized DNA

Digestion of pBR322: Into 8x 1.5 mL Eppendorf tubes was added 5 µg of pBR322 (5 µL from stock of 1 µg/µL in each tube), 165 µL of molecular grade water (to reach total 200 µL reaction volume), 20 µL buffer (10X CutSmart Buffer, NEB Labs) and 5 µL EcoRV-HF (100,000 units/mL, NEB Labs). The tubes were placed in a 37 °C incubator and allowed to incubate for 2.5 hours. A 0.8% gel with two rows of wells was cast (large gel cast with 180 mL of molten agarose solution stained with 18 µL of 10,000X GelRed). The eight digestive reactions were combined (between the eight tubes, a total of 40 µg of pBR322 was digested). 320 µL of Gel Loading Dye Purple 6X (New England Biolabs) was added to the combined sample and the solution was loaded across the individual wells on the gel. The gel was run at 100 V in 1X TAE buffer for 1.5 hours. After electrophoresis, the gel was analyzed using a UV tray in a Bio-Rad ChemiDoc Imaging System to confirm the complete linearization of the DNA. The DNA fragments were cut out from the gel and purified with a QIAquick Gel Extraction Kit (Qiagen). After the purification between two QIAprep® 2.0 spin columns was performed, the DNA was eluted from each with 30 µL molecular grade water. The final concentration of the combined digested DNA was 350 ng/µl (measure by nanodrop). Digestions and purifications performed led to 50% recovery yields of the digested pBR322 DNA.

Cleavage Assay of Linearized pBR322: A fresh 500 µM stock solution of Diazonium salt **23** was prepared by dissolving 10 µmol of the salt in 20 mL of ddH_2O. In 1.5 mL Eppendorf tubes, a serial dilution was performed to achieve a concentration range between 500 nM and 2.5 nM solutions of the diazonium salt in ddH_2O. In 0.2 mL PCR tubes, 0.5 µg of EcoRV-HF digested pBR322 (from the purified sample) and 20 µL of the corresponding diazonium solutions were mixed. The reaction mixtures were incubated at 25°C in between two Kessil pro160 - 525 nm green LED lights (44 W) for 1.5 hours. The DNA-cleaving properties of the diazonium salts were then examined by the standard agarose gel electrophoresis procedure (Figure S11A). The cleavage assay of **23** on linear pBR322 was performed a second time, with the addition of 0.5 µg of supercoiled pBR322 (1 µg/µL), as an internal standard, after the addition of the 6X purple loading dye immediately before loading the samples in the gel (Figure 11B). The DNA-cleaving properties of the diazonium salts were then examined by the standard agarose gel electrophoresis procedure to obtain the does-response plot and an EC_{50} value (Figure S11C).

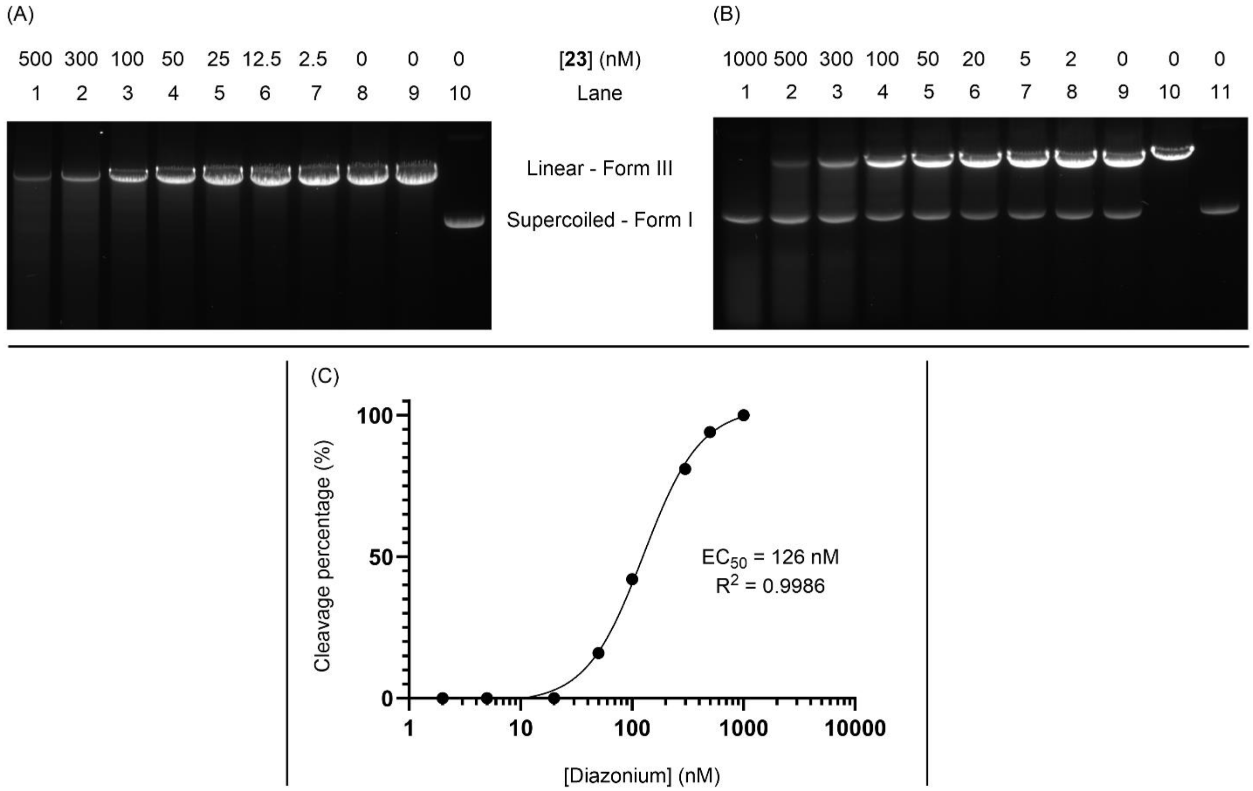

Figure S11. (A) Gel Image of the DNA Cleavage Assay of Linearized pBR322 in presence of Diazonium **23**. In Lanes 1-8: purified EcoRV-HF-digested pBR322 (0.5 µg) and corresponding solution of diazonium **23**; Lane 9: purified EcoRV-HF-digested pBR322 (0.5 µg) with no diazonium nor light irradiation; Lane 10: supercoiled pBR322 plasmid (0.5 µg) with no diazonium nor light irradiation. The cleavage of the pre-linearized pBR322 DNA to lower molecular weight fragments is evident at concentrations ≥ 100 nM (Lanes 1-3). Comparable cleavage profiles were observed by calicheamicin γ1 and shishijimicin A.[5] (B) Gel Image of the DNA Cleavage Assay of Linearized pBR322 in presence of Diazonium **23** with supercoiled pBR322 as an internal standard. In Lanes 1-8: purified EcoRV-HF-digested pBR322 (0.5 µg) and corresponding concentration of diazonium **23** and added supercoiled pBR322 (0.5 µg); Lane 9: Mix of purified EcoRV-HF-digested pBR322(0.5 µg) and supercoiled pBR322 plasmid (0.5 µg) with no diazonium nor light irradiation; Lane 10: purified EcoRV-HF-digested pBR322 (0.5 µg) with no diazonium nor light irradiation; Lane 11: supercoiled pBR322 plasmid (0.5 µg) with no diazonium nor light irradiation. (C) Dose-response curve for the cleavage assay of **23** on linearized pBR322 with supercoiled pBR322 as an internal standard.

X. UV-VIS Spectra for DNA and Diazonium Complexation

Procedure Details: 15.4 µg/mL (25 µM/bp) solutions of supercoiled pBR322 in water and linearized (EcoRV-HF-digested) pBR322 in water were prepared and their absorbances were measured (Figure 2D, solid orange trace and solid blue trace, respectively). A 10 µM solution of **23** in water was prepared and its absorbance was immediately measured (solid red trace). A solution containing both **23** (10 µM) and pBR322 (at 25 µM/bp) was prepared and immediately measured (solid black trace). The spectra did not alter after leaving the sample in the dark for 20 minutes (dotted orange trace). Linearized pBR322 DNA (25 µM/bp) mixed with **23** (10 µM) gave a similar absorbance spectra (dotted blue trace) with a redshift compared to **23** alone and increased absorbance extending up to 500 nm. There was a significant change in the spectra when another solution of **23** (10 µM) and supercoiled pBR322 (25 µM/bp) was prepared and irradiated with 525 nm light for 20 minutes (dotted green trace).

XI. Stability of Diazonium Salts in Water

A fresh batch of diazonium compounds **23** and **11** were prepared following General Procedure B. 8 mg of **23** and 4 mg **11** (~10 mmol) were dissolved in 1 mL of deuterated water in two separate vials. 0.5 µL of acetonitrile was added as an internal standard to each vial. The solutions were added to NMR tubes and the NMR spectra were taken at 0-, 1-, 2-, 6-, and 24-hour time points (Figure S12a and Figure S12b). The mol ratio of the diazonium to internal standard was calculated for each spectrum (diazonium/IS) and the ratio from each time point was compared to the ratio from the starting spectra to calculate the percentage of diazonium in solution at each point.

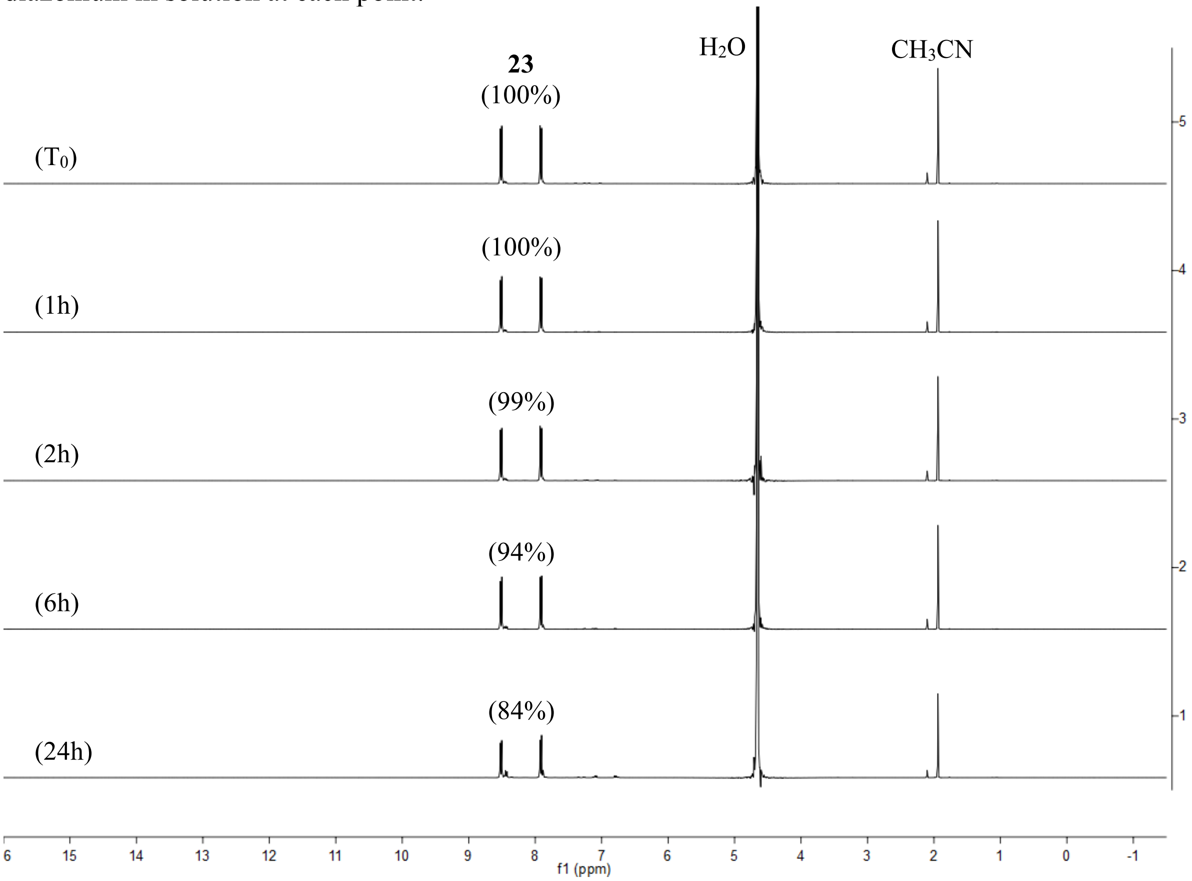

Figure S12a. NMR spectra of **23** in D_2O at different time points. (T_0) spectra immediately after sample preparation of **23** with a **23**/IS ratio of 0.68; (1h) spectra at 1 hour with a **23**/IS ratio of 0.68; (2h) spectra at 2 hours with a **23**/IS ratio of 0.67; (6h) spectra at 6 hours with a **23**/IS ratio of 0.64; (24h) spectra at 24 hours with a **23**/IS ratio of 0.54.

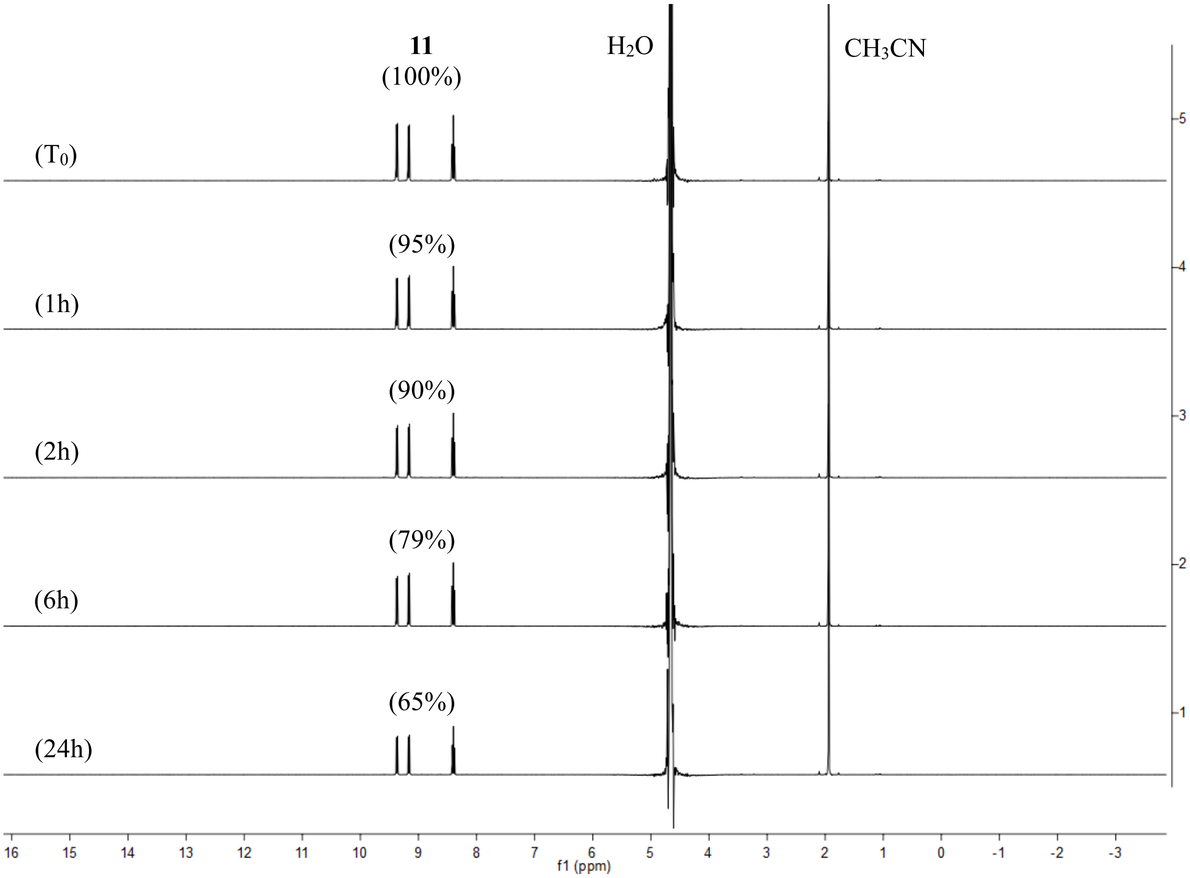

Figure S12b. NMR spectra of **11** in D_2O at different time points**.** (T_0) spectra immediately after sample preparation of **11** with a **11**/IS ratio of 0.62; (1h) spectra at 1 hour with a **11**/IS ratio of 0.59; (2h) spectra at 2 hours with a **11**/IS ratio of 0.56; (6h) spectra at 6 hours with a **11**/IS ratio of 0.49; (24h) spectra at 24 hours with a **11**/IS ratio of 0.41.

XII. Gel Images and EC_{50} Plots

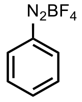

1

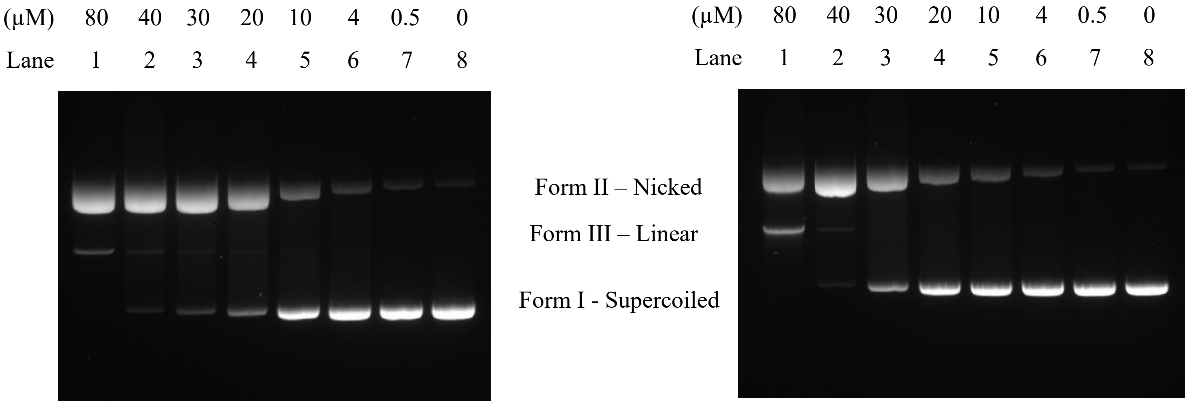

Figure S13a. Agarose Gel Images Using Diazonium **1**

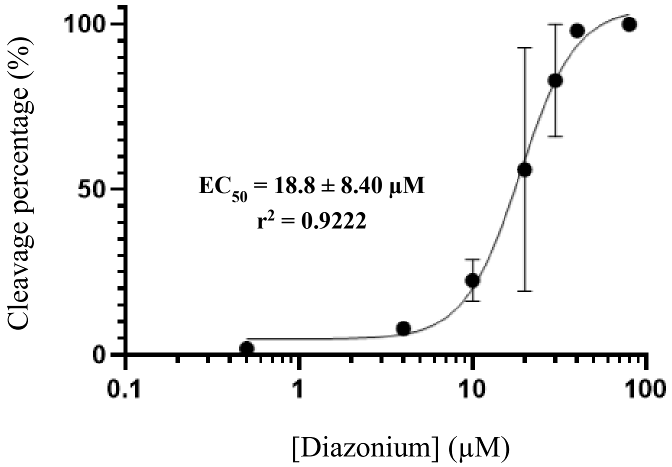

Figure S13b. Dose Response Plot of Cleaved DNA Percentage against Concentration of **1**

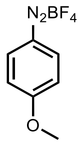

2

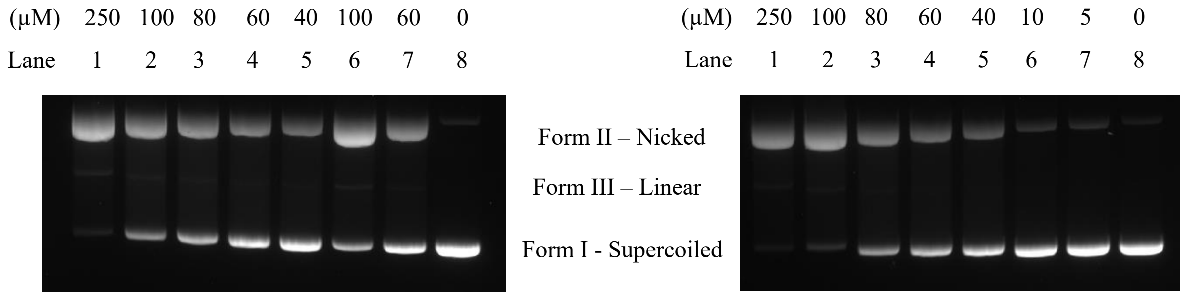

Figure S14a. Agarose Gel Images Using Diazonium **2**

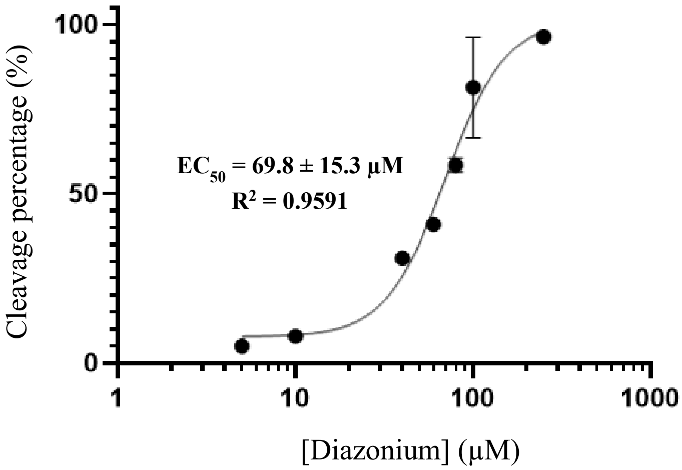

Figure S14b. Dose Response Plot of Cleaved DNA Percentage against Concentration of **2**

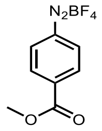

3

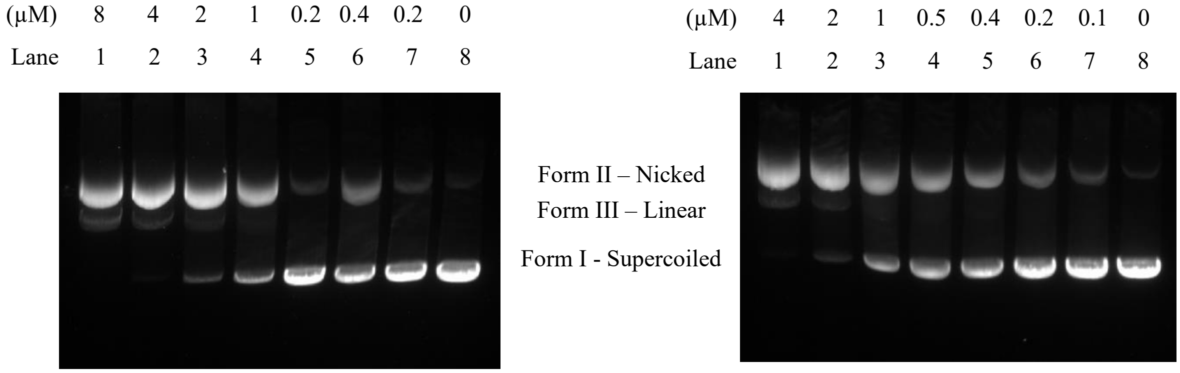

Figure S15a. Agarose Gel Images Using Diazonium **3**

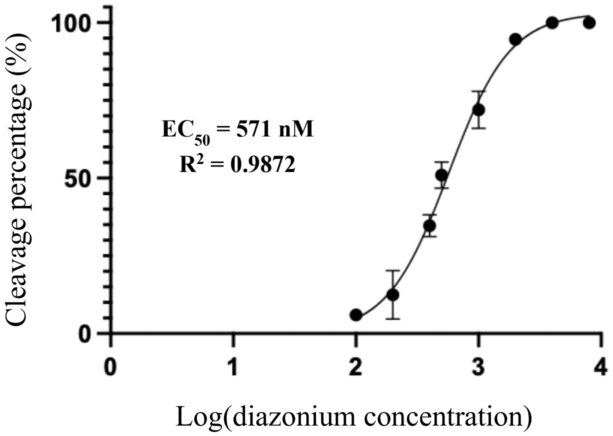

Figure S15b. Dose Response Plot of Cleaved DNA Percentage against Concentration of **3**

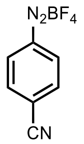

4

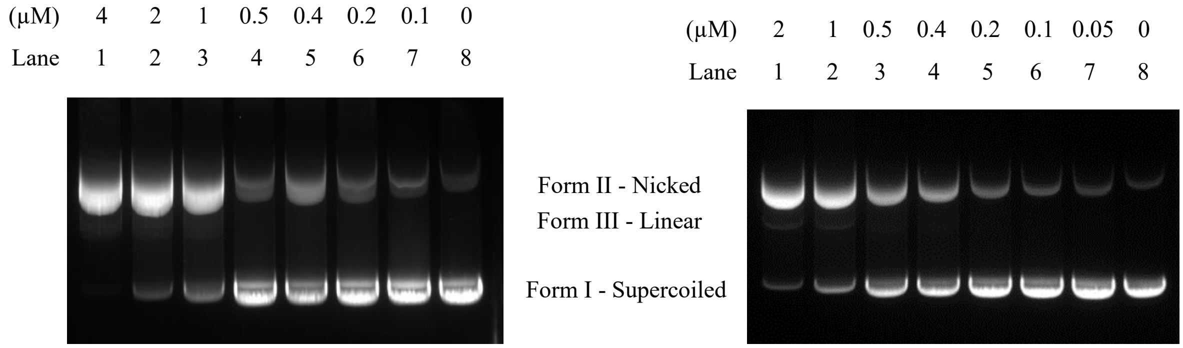

Figure S16a. Agarose Gel Images Using Diazonium **4**

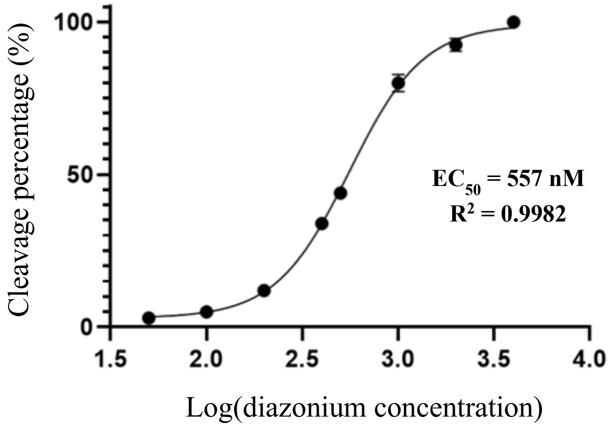

Figure S16b. Dose Response Plot of Cleaved DNA Percentage against Concentration of **4**

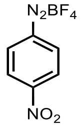

5

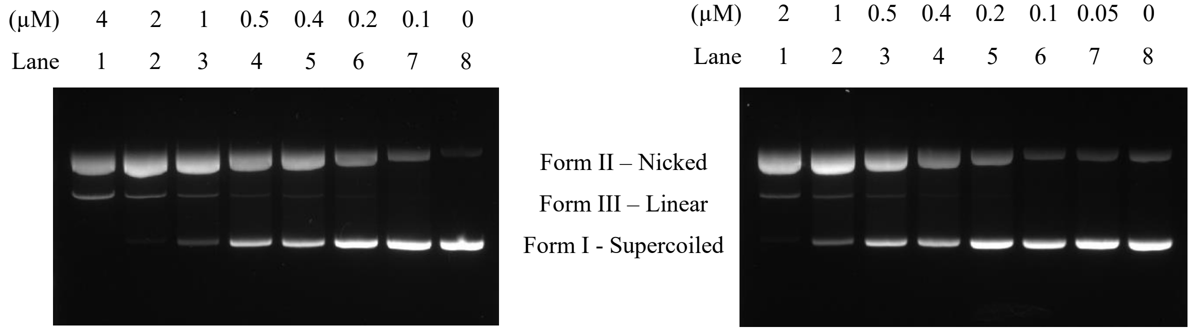

Figure S17a. Agarose Gel Images Using Diazonium **5**

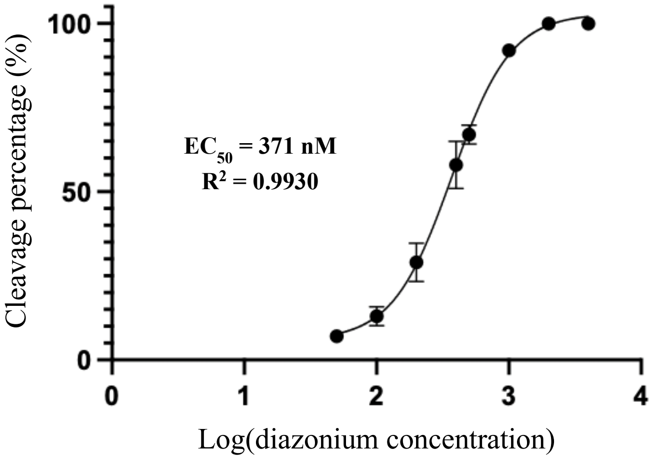

Figure S17b. Dose Response Plot of Cleaved DNA Percentage against Concentration of **5**

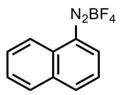

6

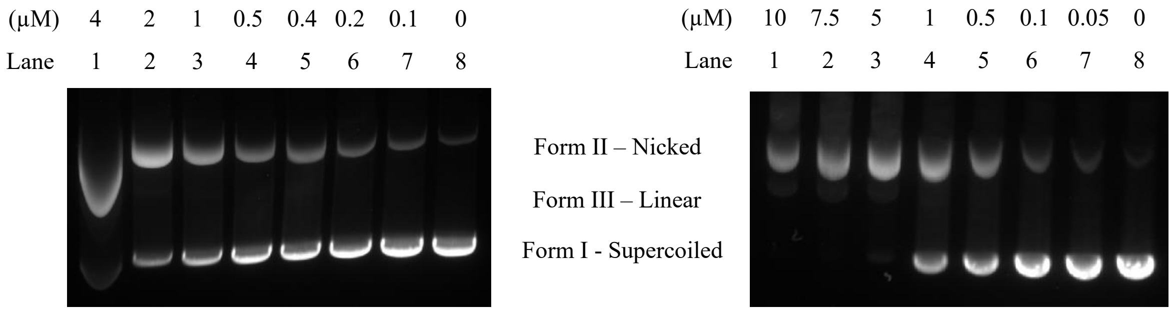

Figure S18a. Agarose Gel Images Using Diazonium **6**

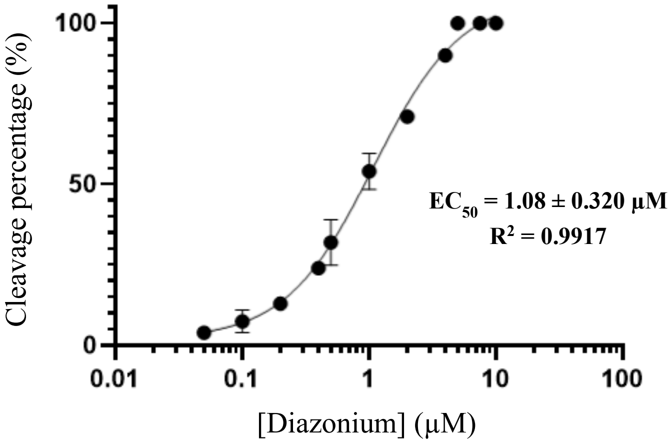

Figure S18b. Dose Response Plot of Cleaved DNA Percentage against Concentration of **6**

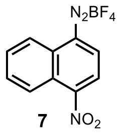

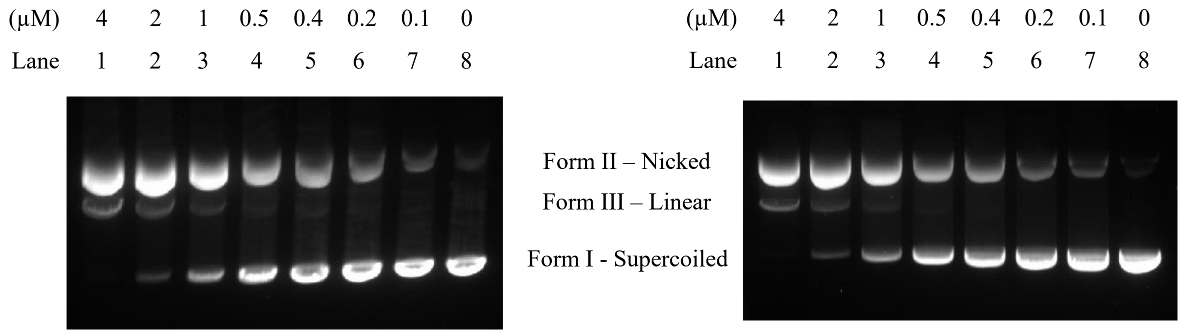

Figure S19a. Agarose Gel Images Using Diazonium **7**

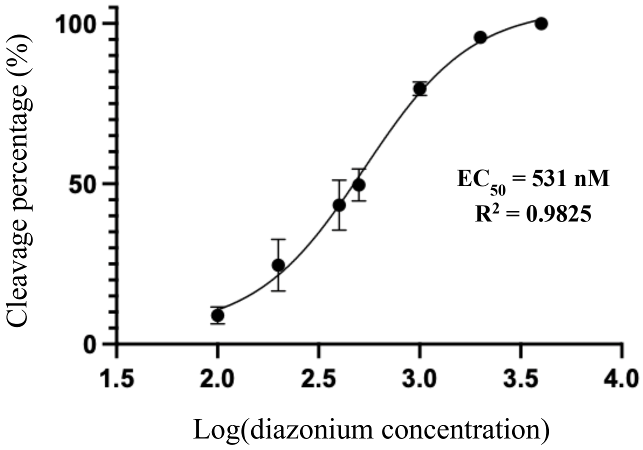

Figure S19b. Dose Response Plot of Cleaved DNA Percentage against Concentration of **7**

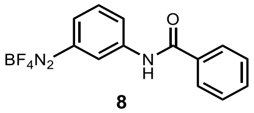

8

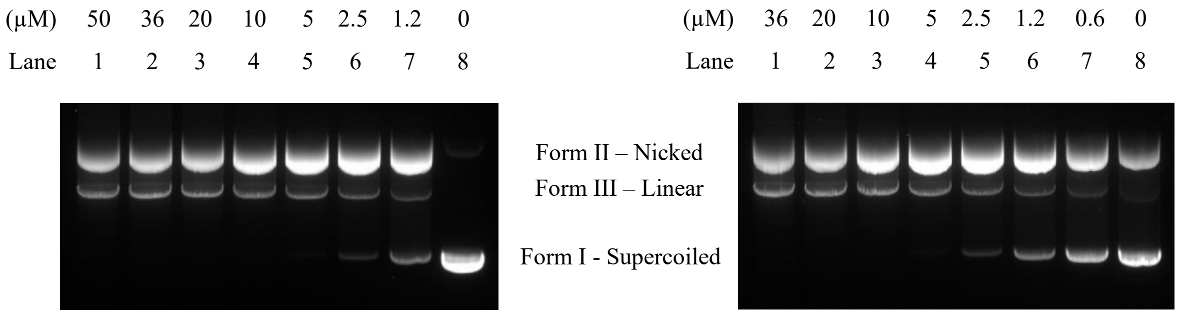

Figure S20a. Agarose Gel Images Using Diazonium **8**

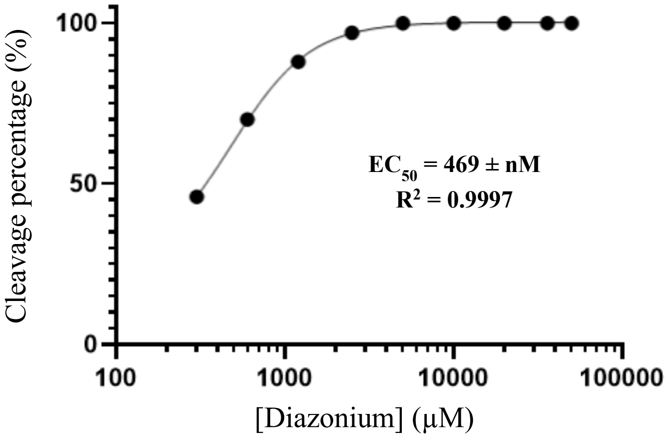

Figure S20b. Dose Response Plot of Cleaved DNA Percentage against Concentration of **8**

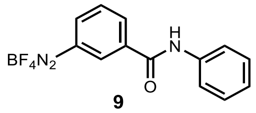

9

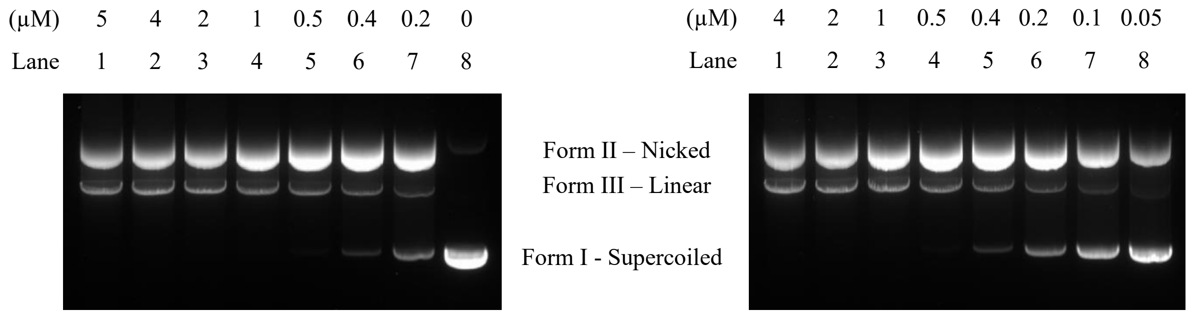

Figure S21a. Agarose Gel Images Using Diazonium **9**

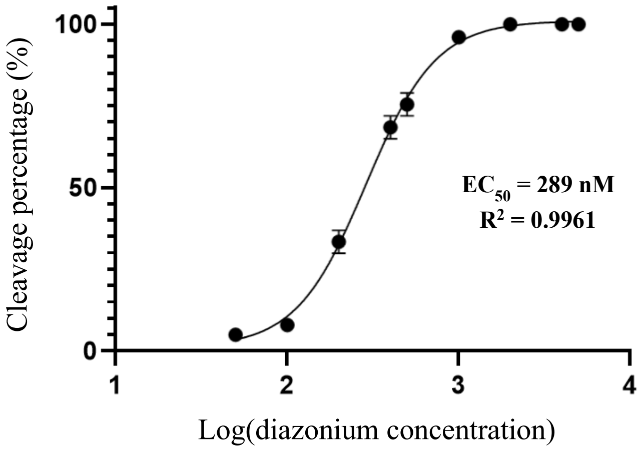

Figure S21b. Dose Response Plot of Cleaved DNA Percentage against Concentration of **9**

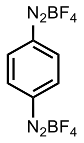

10

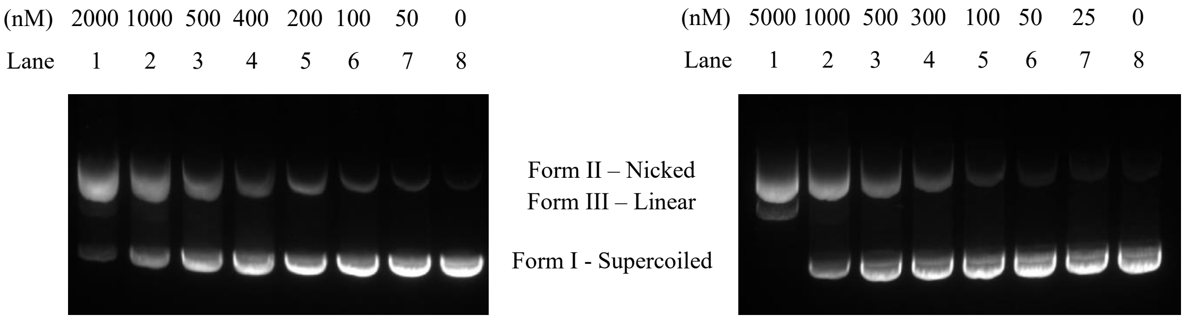

Figure S22a. Agarose Gel Images Using Diazonium **10**

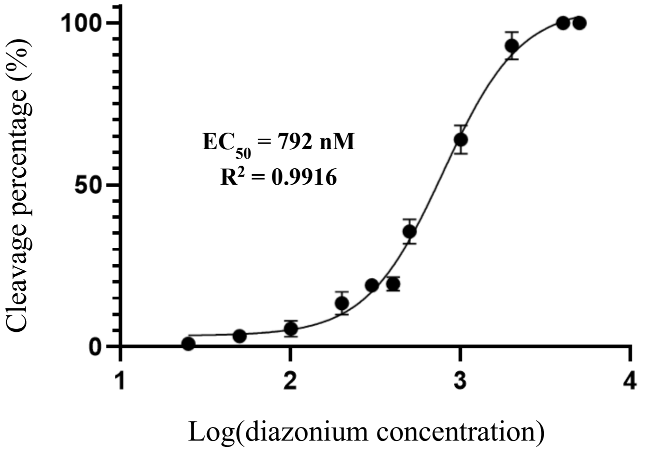

Figure S22b. Dose Response Plot of Cleaved DNA Percentage against Concentration of **10**

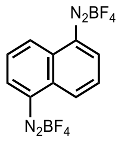

11

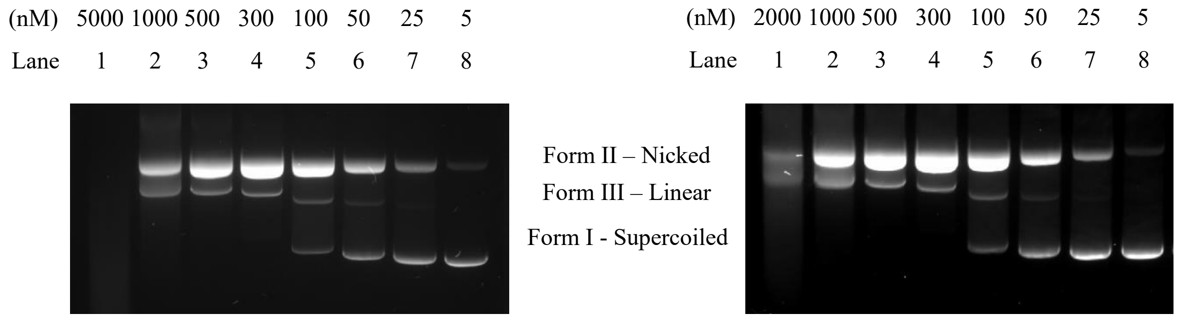

Figure S23a. Agarose Gel Images Using Diazonium **11**

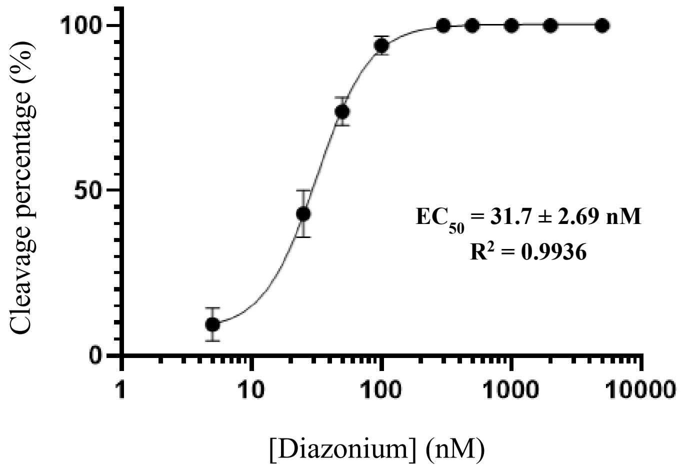

Figure S23b. Dose Response Plot of Cleaved DNA Percentage against Concentration of **11**

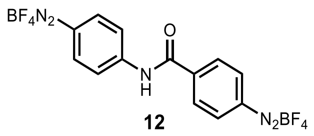

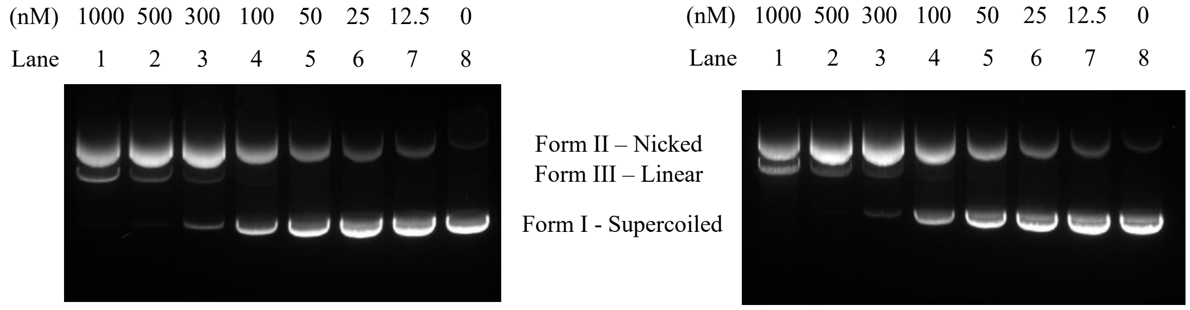

Figure S24a. Agarose Gel Images Using Diazonium **12**

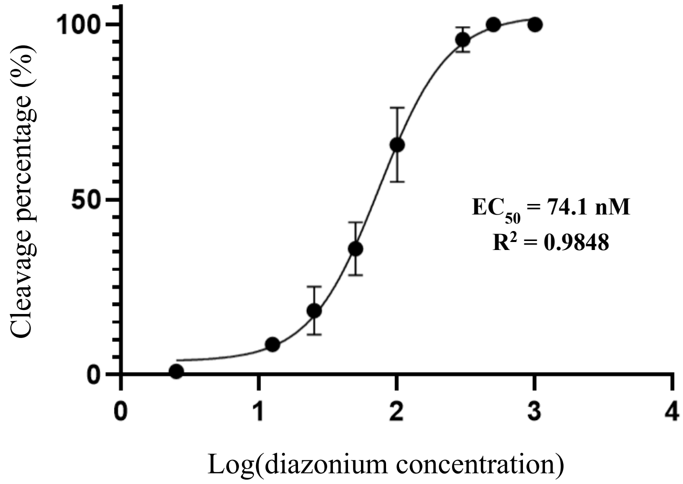

Figure S24b. Dose Response Plot of Cleaved DNA Percentage against Concentration of **12**

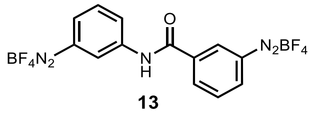

13

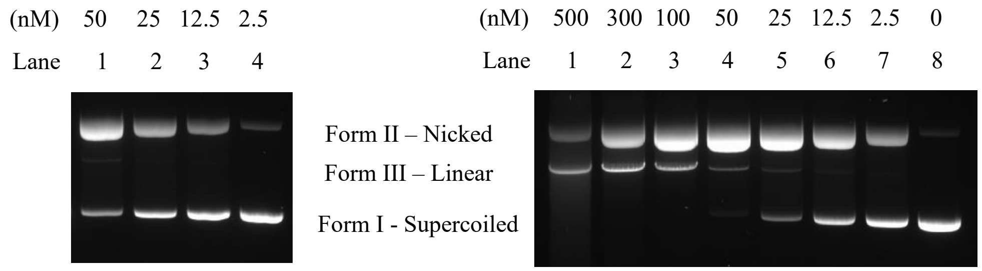

Figure S25a. Agarose Gel Images Using Diazonium **13**

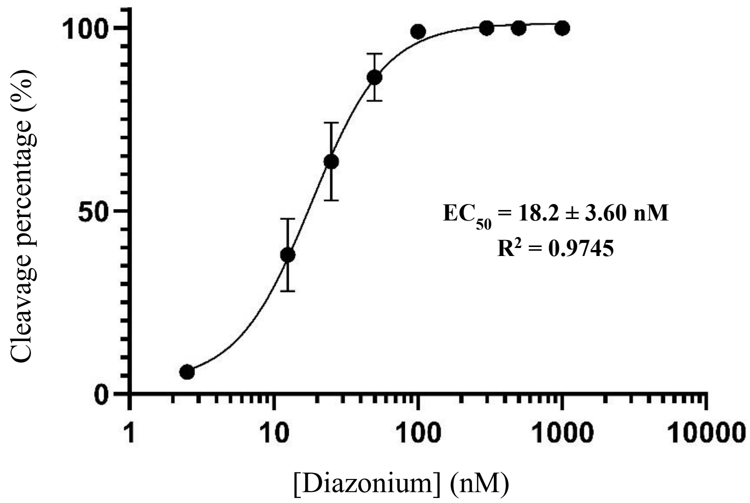

Figure S25b. Dose Response Plot of Cleaved DNA Percentage against Concentration of **13**

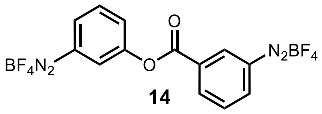

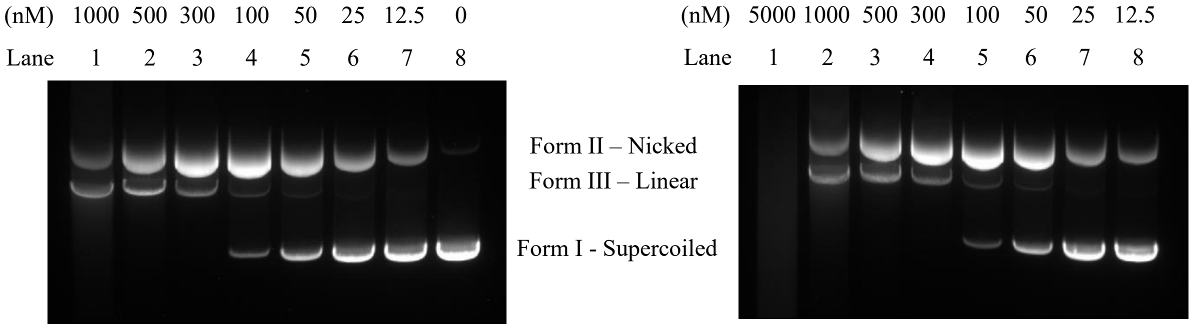

Figure S26a. Agarose Gel Images Using Diazonium **14**

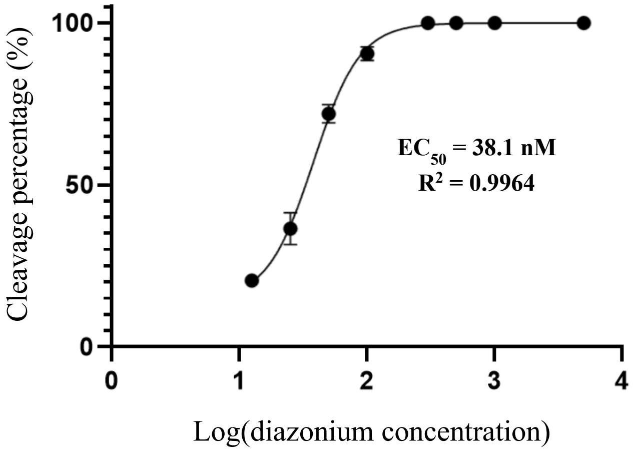

Figure S26b. Dose Response Plot of Cleaved DNA Percentage against Concentration of **14**

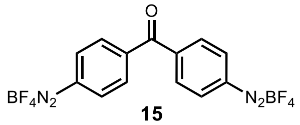

15

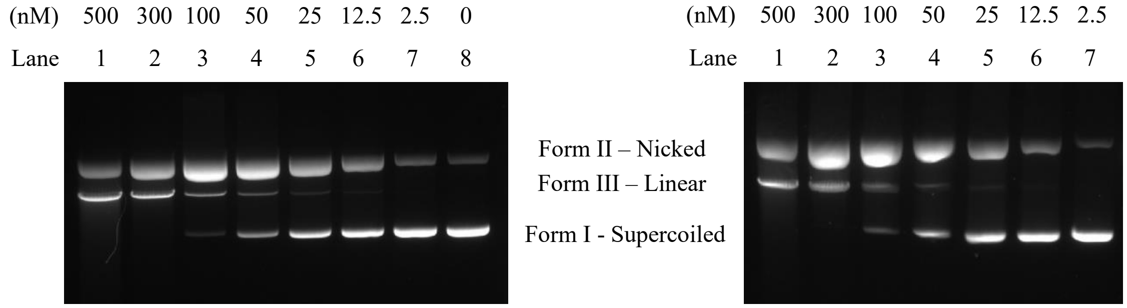

Figure S27a. Agarose Gel Images Using Diazonium **15**

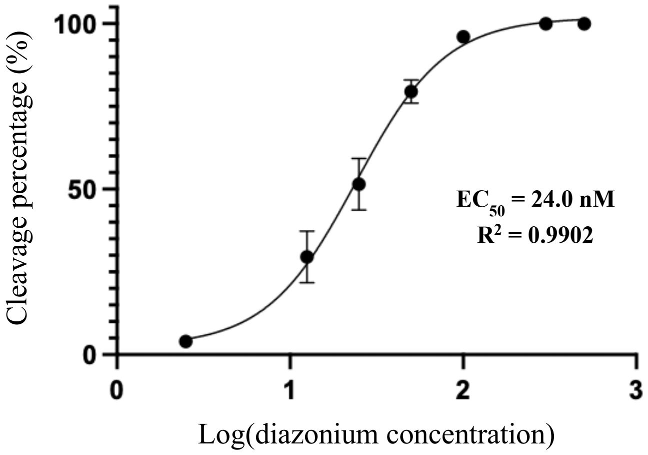

Figure S27b. Dose Response Plot of Cleaved DNA Percentage against Concentration of **15**

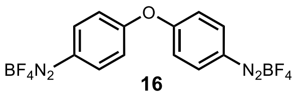

16

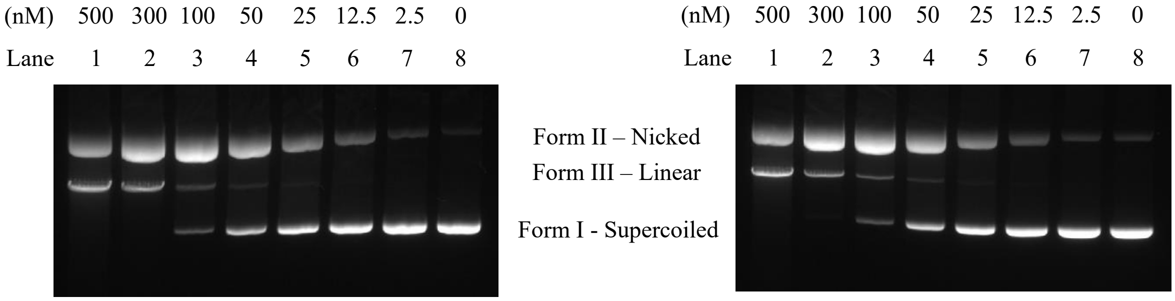

Figure S28a. Agarose Gel Images Using Diazonium **16**

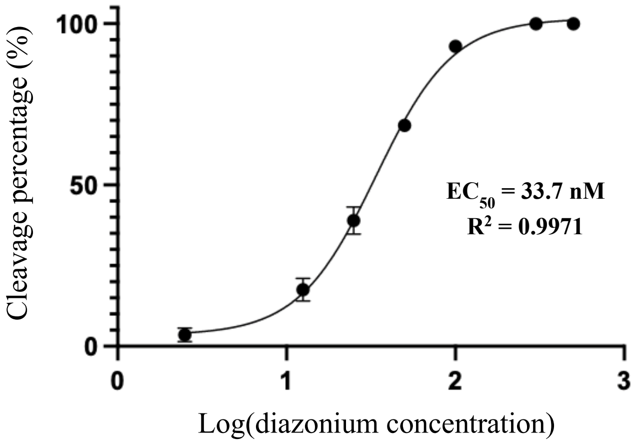

Figure S28b. Dose Response Plot of Cleaved DNA Percentage against Concentration of **16**

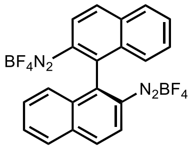

17

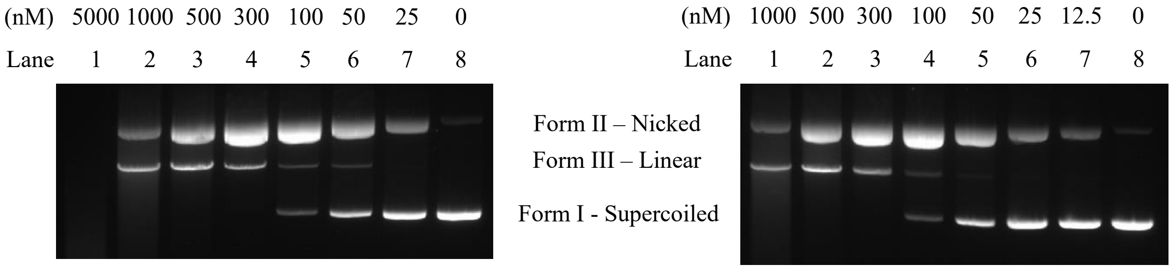

Figure S29a. Agarose Gel Images Using Diazonium **17**

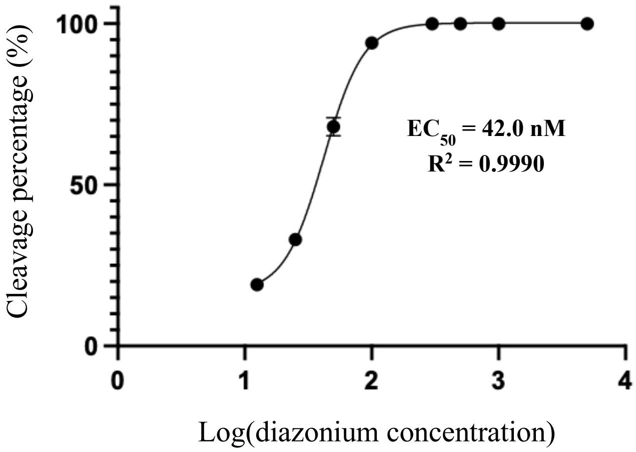

Figure S29b. Dose Response Plot of Cleaved DNA Percentage against Concentration of **17**

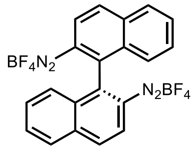

18

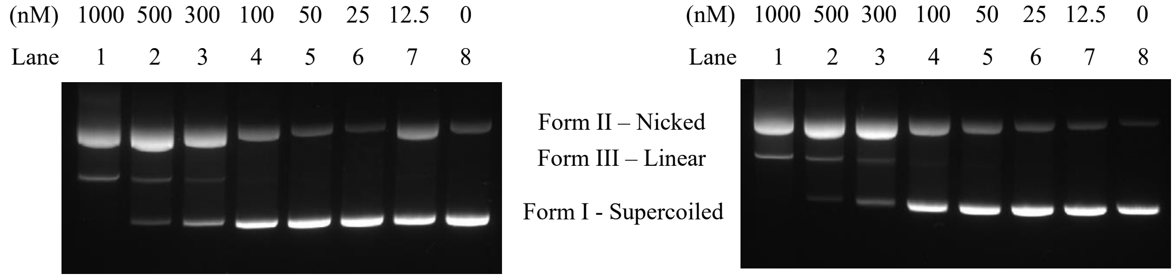

Figure S30a. Agarose Gel Images Using Diazonium **18**

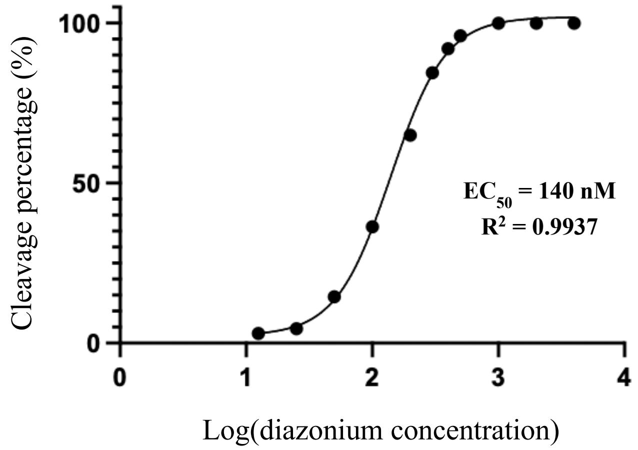

Figure S30b. Dose Response Plot of Cleaved DNA Percentage against Concentration of **18**

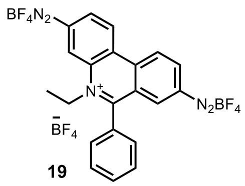

19

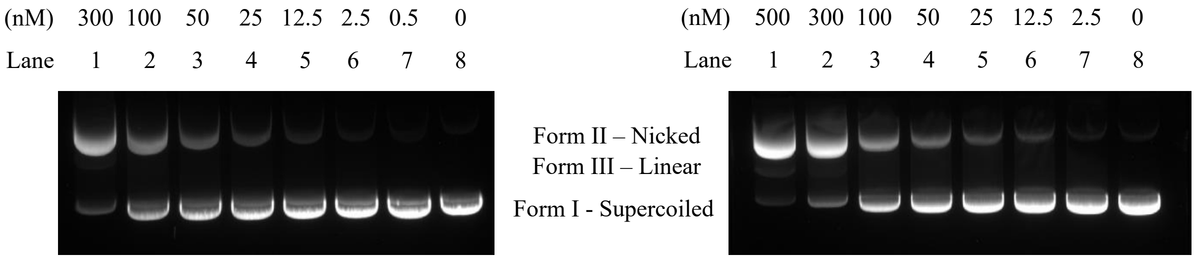

Figure S31a. Agarose Gel Images Using Diazonium **19**

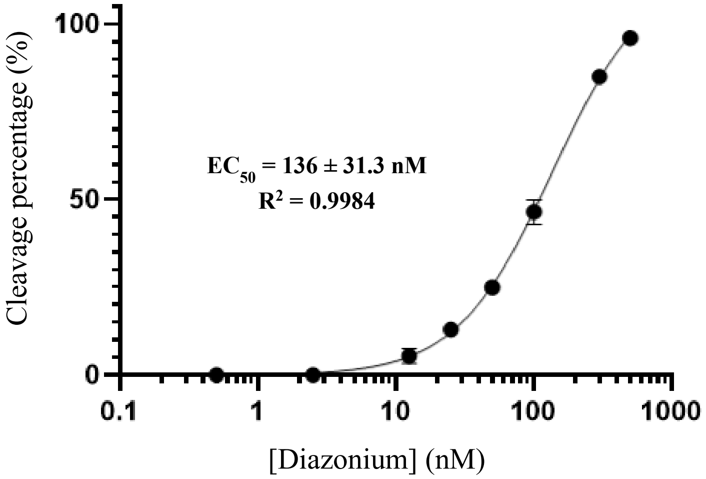

Figure S31b. Dose Response Plot of Cleaved DNA Percentage against Concentration of **19**

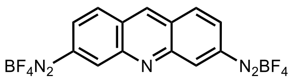

20

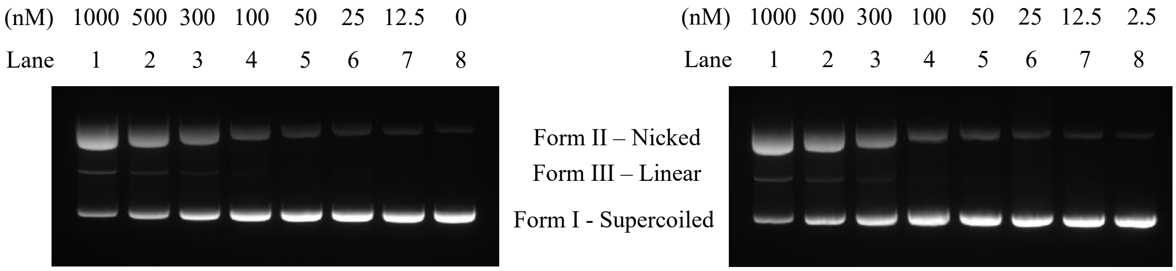

Figure S32a. Agarose Gel Images Using Diazonium **20**

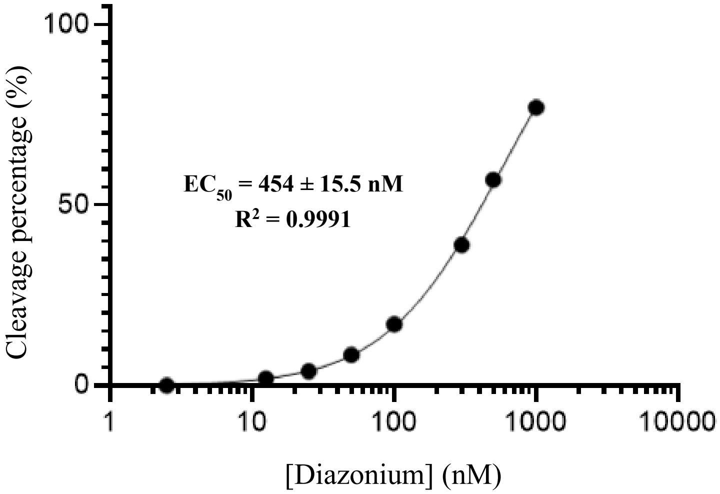

Figure S32b. Dose Response Plot of Cleaved DNA Percentage against Concentration of **20**

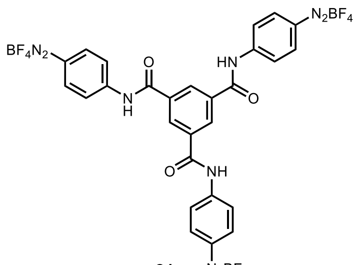

21

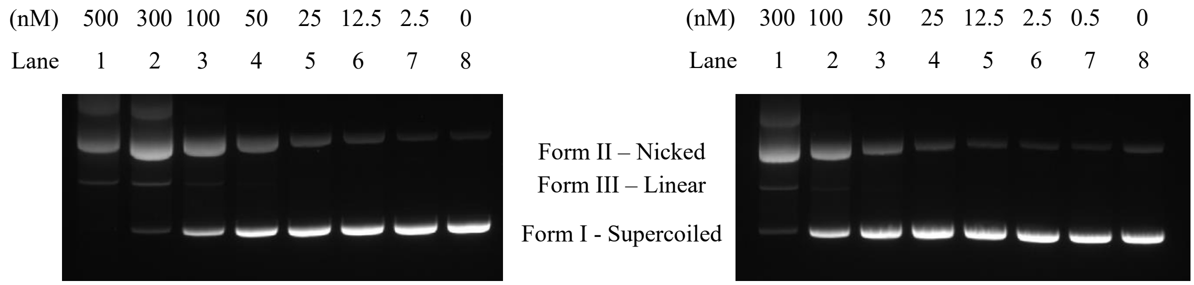

Figure S33a. Agarose Gel Images Using Diazonium **21**

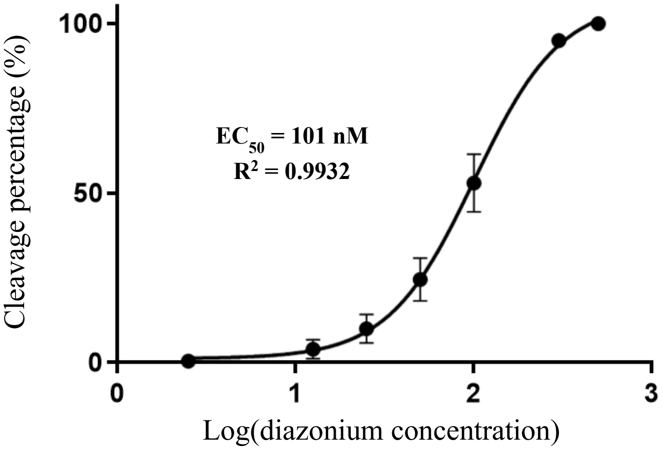

Figure S33b. Dose Response Plot of Cleaved DNA Percentage against Concentration of **21**

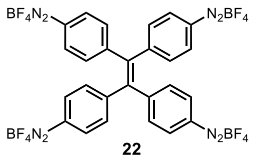

22

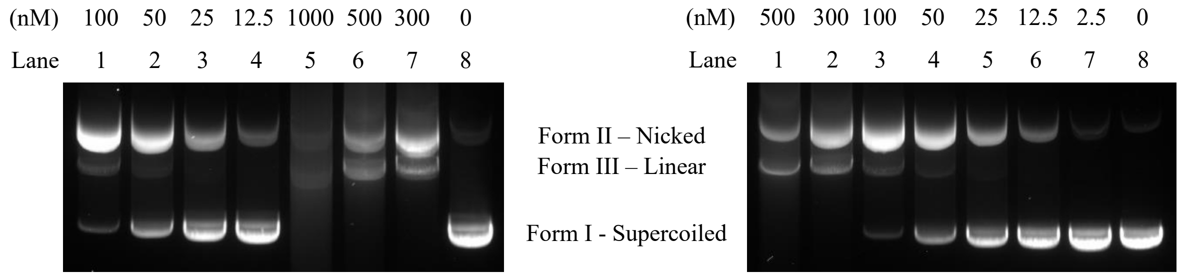

Figure S34a. Agarose Gel Images Using Diazonium **22**

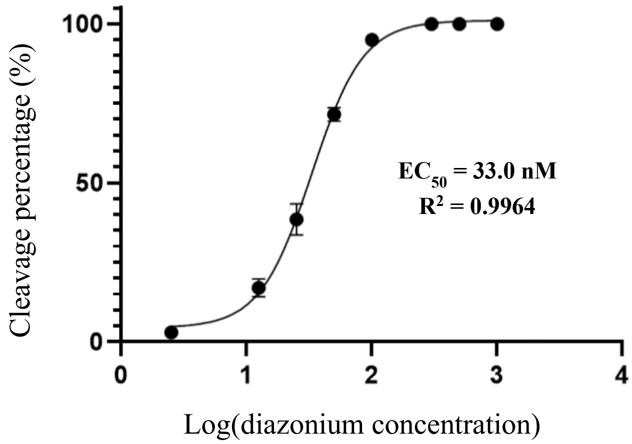

Figure S34b. Dose Response Plot of Cleaved DNA Percentage against Concentration of **22**

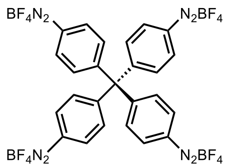

23

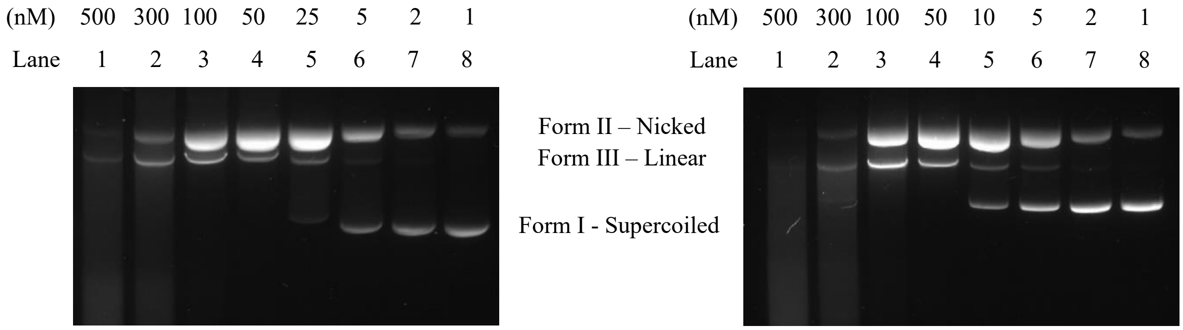

Figure S35a. Agarose Gel Images Using Diazonium **23**

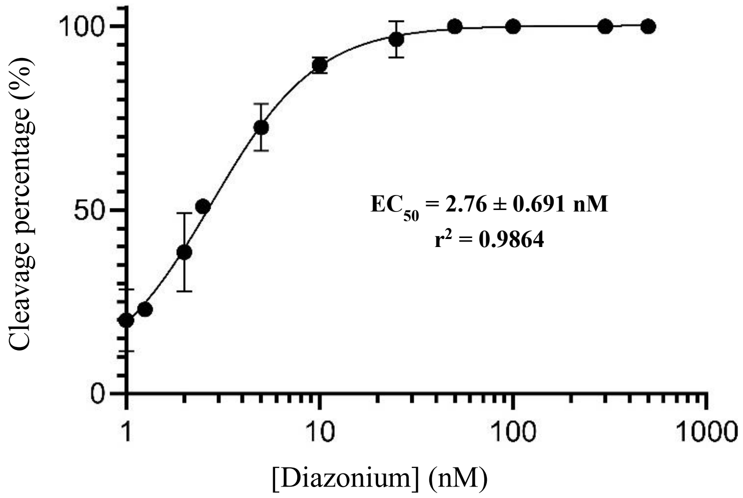

Figure S35b. Dose Response Plot of Cleaved DNA Percentage against Concentration of **23**

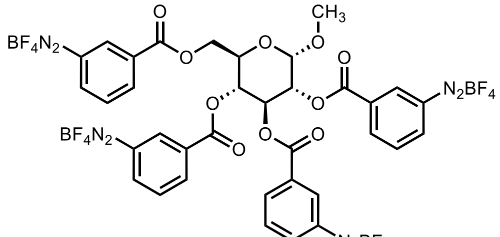

24

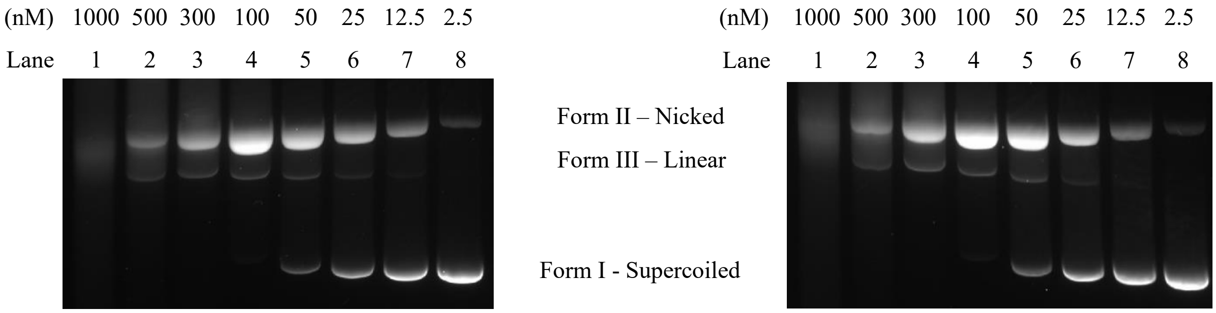

Figure S36a. Agarose Gel Images Using Diazonium **24**

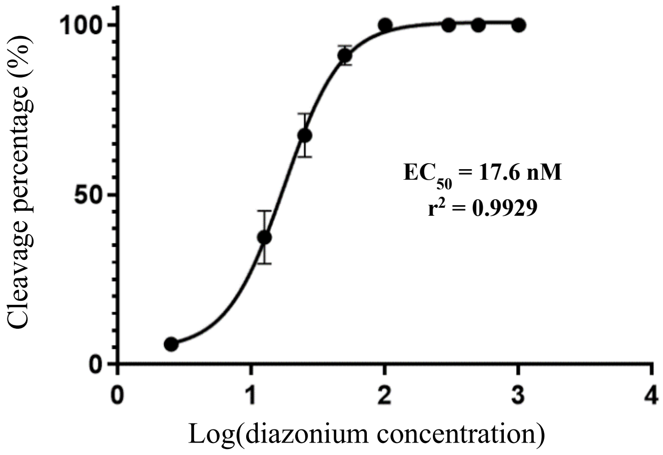

Figure S36b. Dose Response Plot of Cleaved DNA Percentage against Concentration of **24**

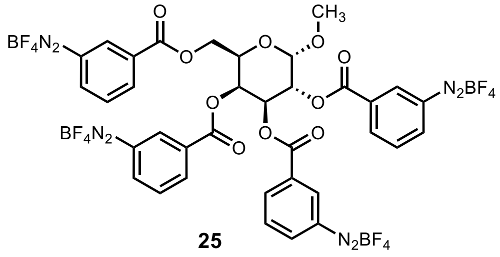

25

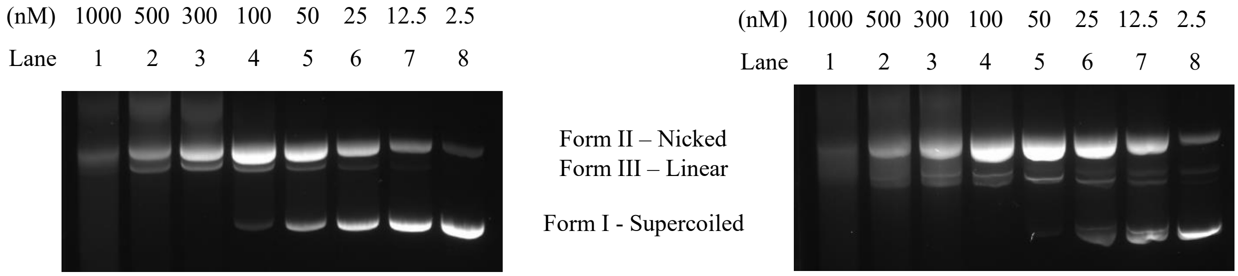

Figure S37a. Agarose Gel Images Using Diazonium **25**

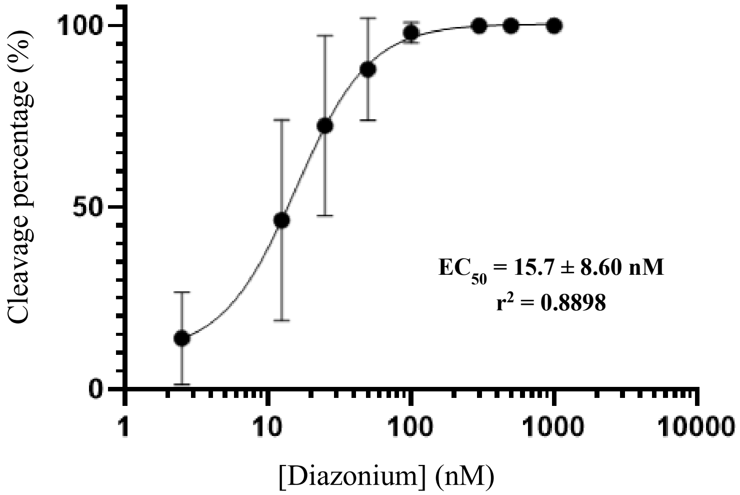

Figure S37b. Dose Response Plot of Cleaved DNA Percentage against Concentration of **25**

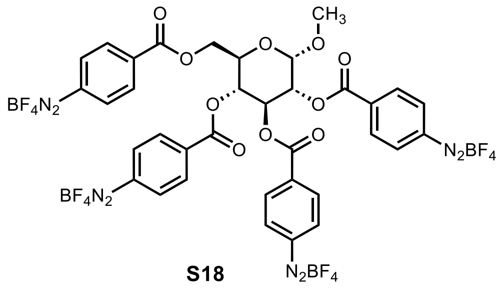

S18

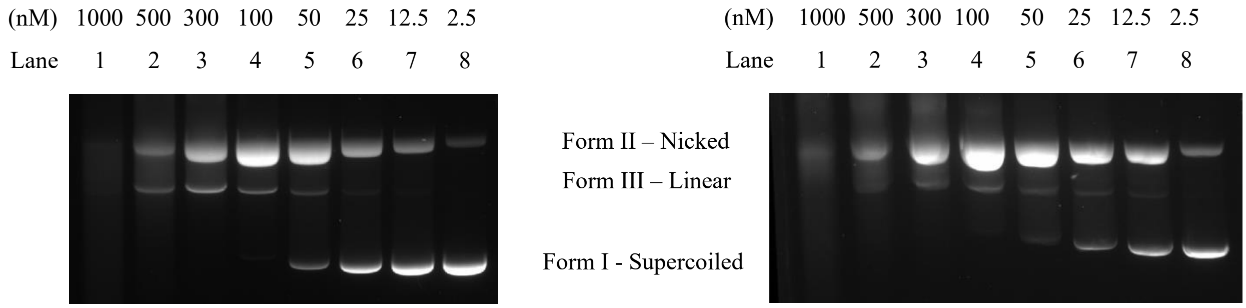

Figure S38a. Agarose Gel Images Using Diazonium **S18**

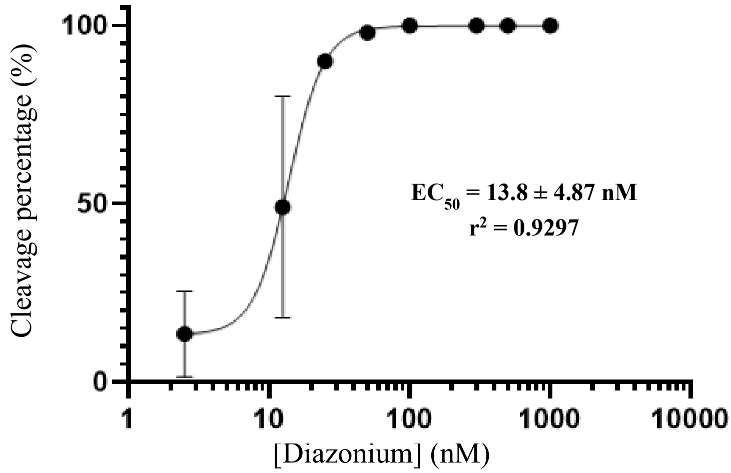

Figure S38b. Dose Response Plot of Cleaved DNA Percentage against Concentration of **S18**

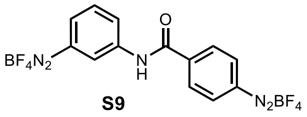

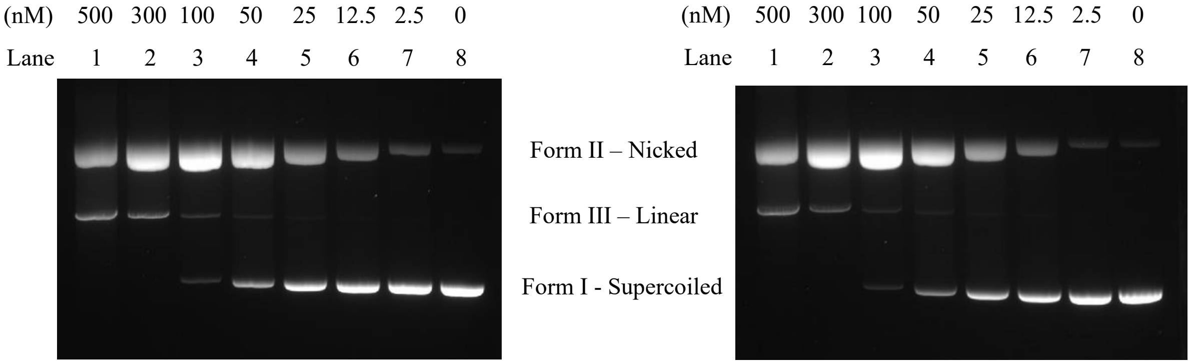

Figure S39a. Agarose Gel Images Using Diazonium **S9**

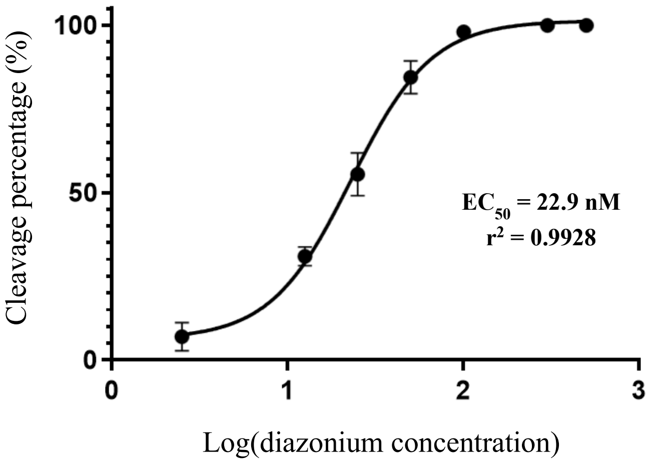

Figure S39b. Dose Response Plot of Cleaved DNA Percentage against Concentration of **S9**

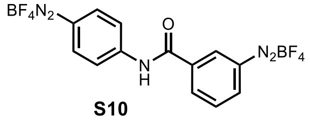

S10

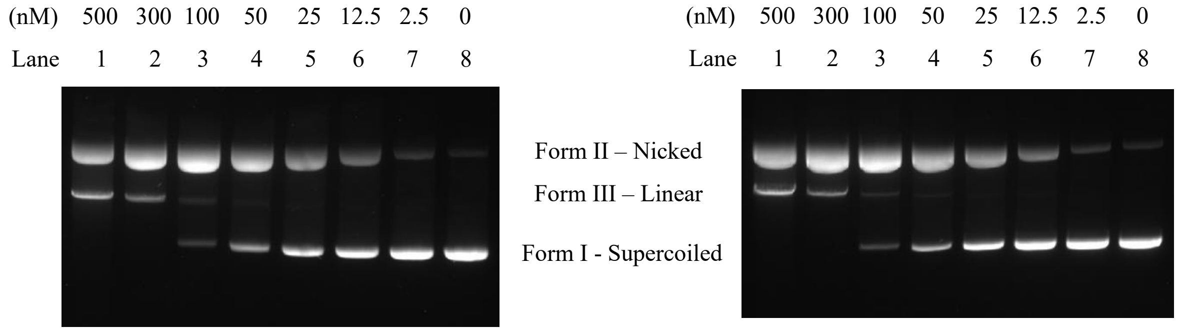

Figure S40a. Agarose Gel Images Using Diazonium **S10**

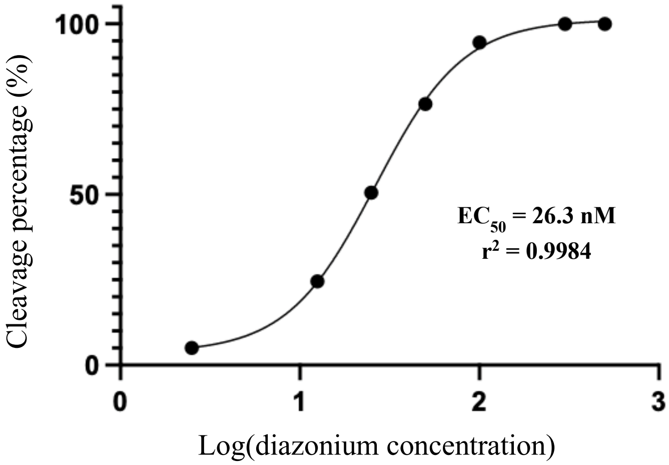

Figure S40b. Dose Response Plot of Cleaved DNA Percentage against Concentration of **S10**

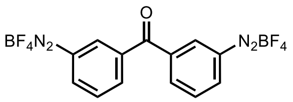

S11

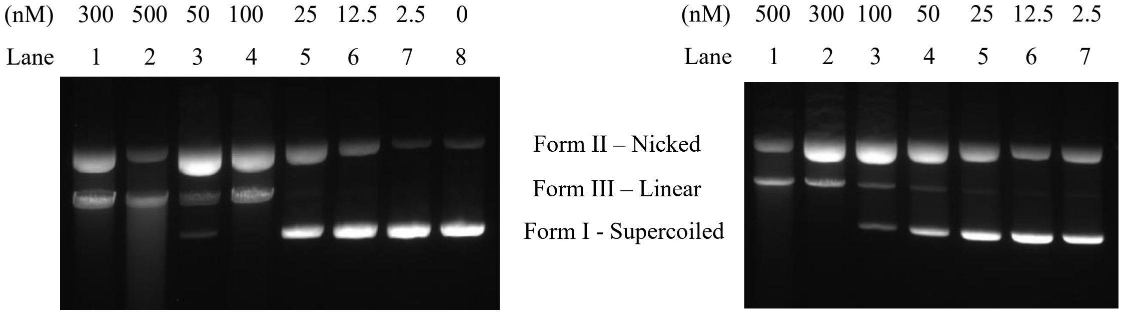

Figure S41a. Agarose Gel Images Using Diazonium **S11**

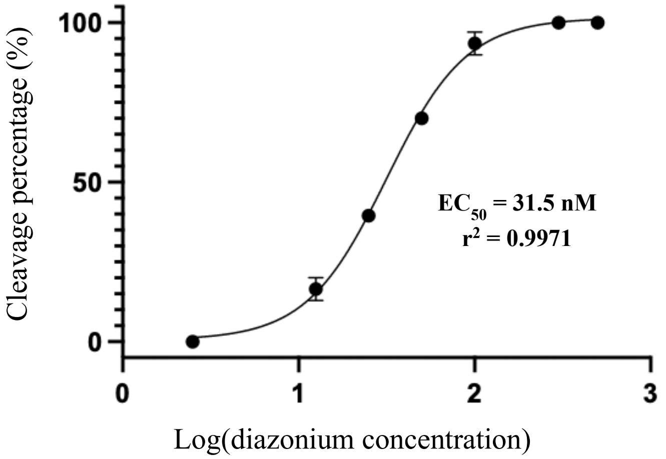

Figure S41b. Dose Response Plot of Cleaved DNA Percentage against Concentration of **S11**

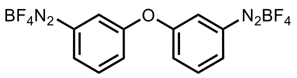

S12

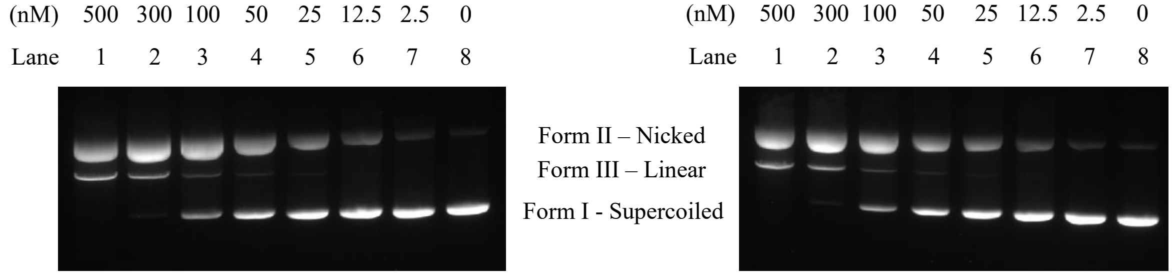

Figure S42a. Agarose Gel Images Using Diazonium **S12**

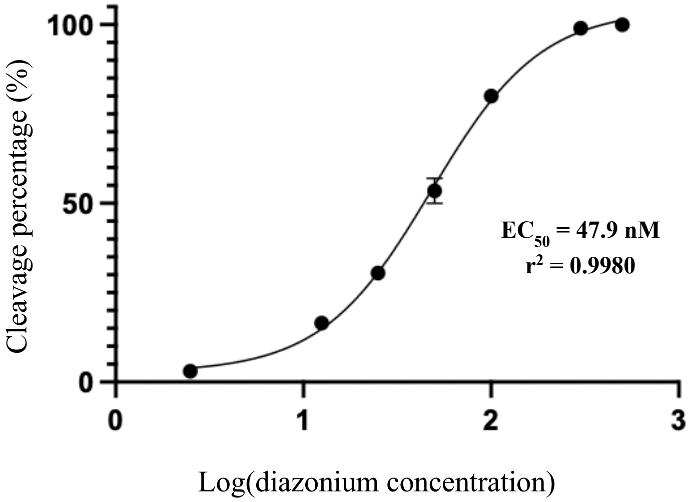

Figure S42b. Dose Response Plot of Cleaved DNA Percentage against Concentration of **S12**

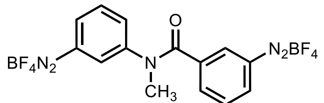

S14

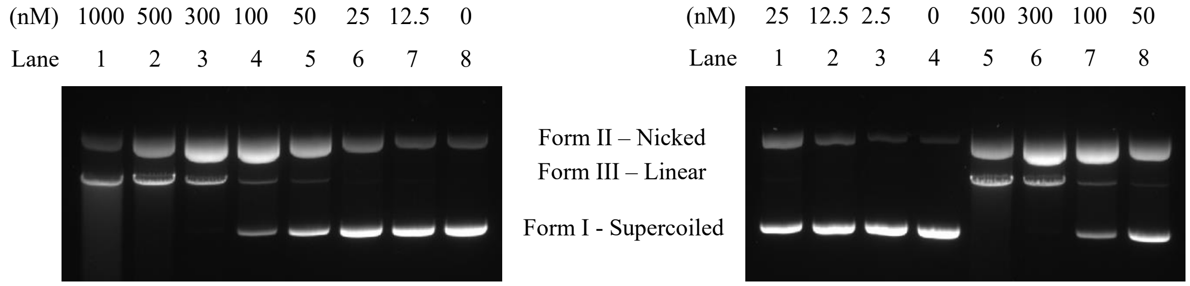

Figure S43a. Agarose Gel Images Using Diazonium **S14**

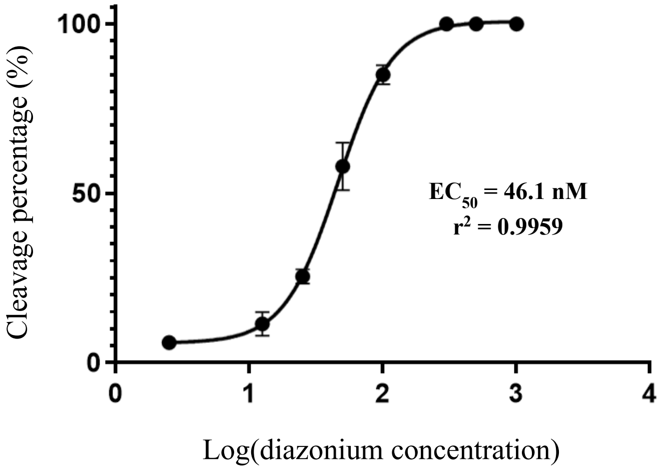

Figure S43b. Dose Response Plot of Cleaved DNA Percentage against Concentration of **S14**

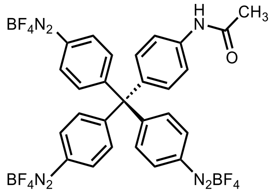

S16

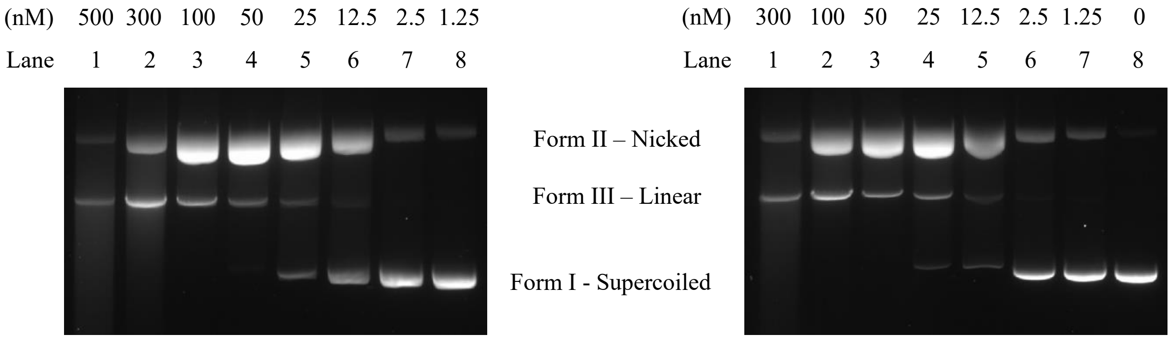

Figure S44a. Agarose Gel Images Using Diazonium **S16**

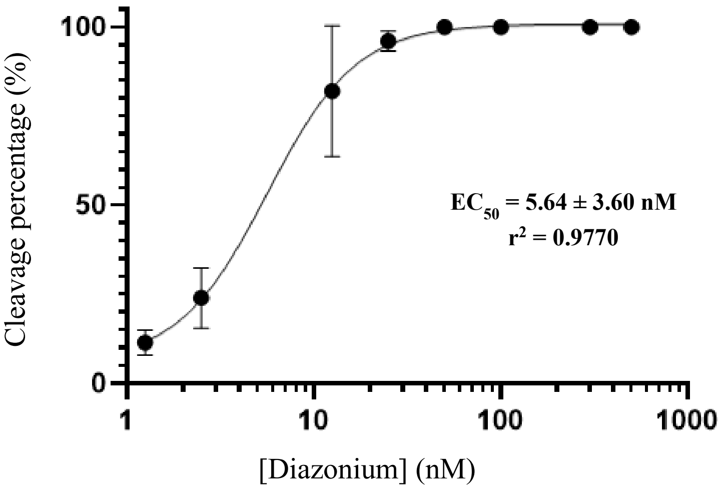

Figure S44b. Dose Response Plot of Cleaved DNA Percentage against Concentration of **S16**

XIII. Cell Culture and Cell Viability Procedures

1. General Cell Culture Protocol

HeLa cells were grown in DMEM growth media (GIBCO 11995065) with 10% FBS (GIBCO A5670401) in 75 cm^2 surface treated culture dishes (Fisherbrand FB012937) and were incubated in a cell culture chamber (37 °C with 5% CO_2). Cells were kept at a maximum confluency of 80% and passaged up to 20 times. For each passage, the cells were washed with 1X PBS (GIBCO 20012027), detached from the plate by incubation with 0.25% Trypsin-EDTA (GIBCO 25200072) for 3-4 minutes in the cell culture chamber and then diluted with growth media. The cells were spun down at 300 xg at 4 °C for 5 minutes, resuspended with fresh growth media, and replated in a new flask.

2. Cell Viability Assay and IC_{50} Procedure

The media in the cell culture dish was aspirated, and the cells washed with 1X PBS. The cells were detached from the plate by 4-minute incubation with 0.25% Trypsin-EDTA in the cell culture chamber. The cells were then diluted with DMEM and were spun down at 300 xg at 4 °C for 5 minutes. The media was aspirated, and the cell pellet was resuspended in fresh DMEM. The cells were counted and seeded in 6-well culture plates at 50,000 cells/well and incubated overnight. Alongside the 6-well plates, 35 mm glass bottom confocal plates (Fisher Scientific NC0409658) were also seeded with 50,000 cells/plate. 24 hours after the initial seeding, the media in the plates were aspirated and the cells were washed with 1X PBS. A 100 µM stock solution of diazonium **23** was prepared in 1X PBS pH 6.6 and a serial dilution was performed to achieve a concentration range of 20 µM to 1.25 µM of **23** in 1X PBS pH 6.6. The wash buffer was aspirated from the plates and 2 mL of the corresponding diazonium dilutions was added to the respective wells in the 6-well plates, all in triplicate, in addition to the confocal plates. All plates were incubated in ambient light for 45 minutes in the cell culture hood and then for 1 hour in the cell culture chamber (37 °C with 5% CO_2). *Control wells with 2 mL 1X PBS pH 6.6 alone with no diazonium as well as additional control wells with 2 mL DMEM alone with no diazonium, all in triplicate, were also incubated alongside the wells with the diazonium solutions.* The plates were then removed from the incubators and the solutions from all the dishes were aspirated and replaced with 2 mL of fresh DMEM. The plates were incubated in the cell culture chamber for 3 days. After the 3-day incubation, the respective cell viability of each well was measured using the PrestoBlue™ Cell Viability Procedure. To each well was added 200 µL of 10X PrestoBlue™ Cell Viability Reagent (ThermoFisher Scientific A13261) and the plates were then incubated in the cell culture chamber for 3 hours. The plates were then analyzed using a plate reader (SpectraMax iD5) by measuring the fluorescence at an excitation of 560 nm and emission of 590 nm. The data was exported from SoftMax Pro7.1 software and used to calculate the relative viability of each well compared with the control wells. The values were plotted against the corresponding diazonium concentrations in a dose-response plot to generate the IC_{50} value (Figure S45A). The cells in the 35 mm glass bottom confocal plates were also imaged after the 3-day incubation using confocal microscopy (Figure S45B). Brightfield imaging was performed using a Leica SP8 confocal microscope equipped with a 10x objective. The TLD PMT Trans detector was used to capture transmitted light images of the cell culture plates. *Note that the confluency and viability of both controls without diazonium, either incubated in 1X PBS pH 6.6 or DMEM, had similar viability and confluency* (Figure S45B Images 1 and 2).

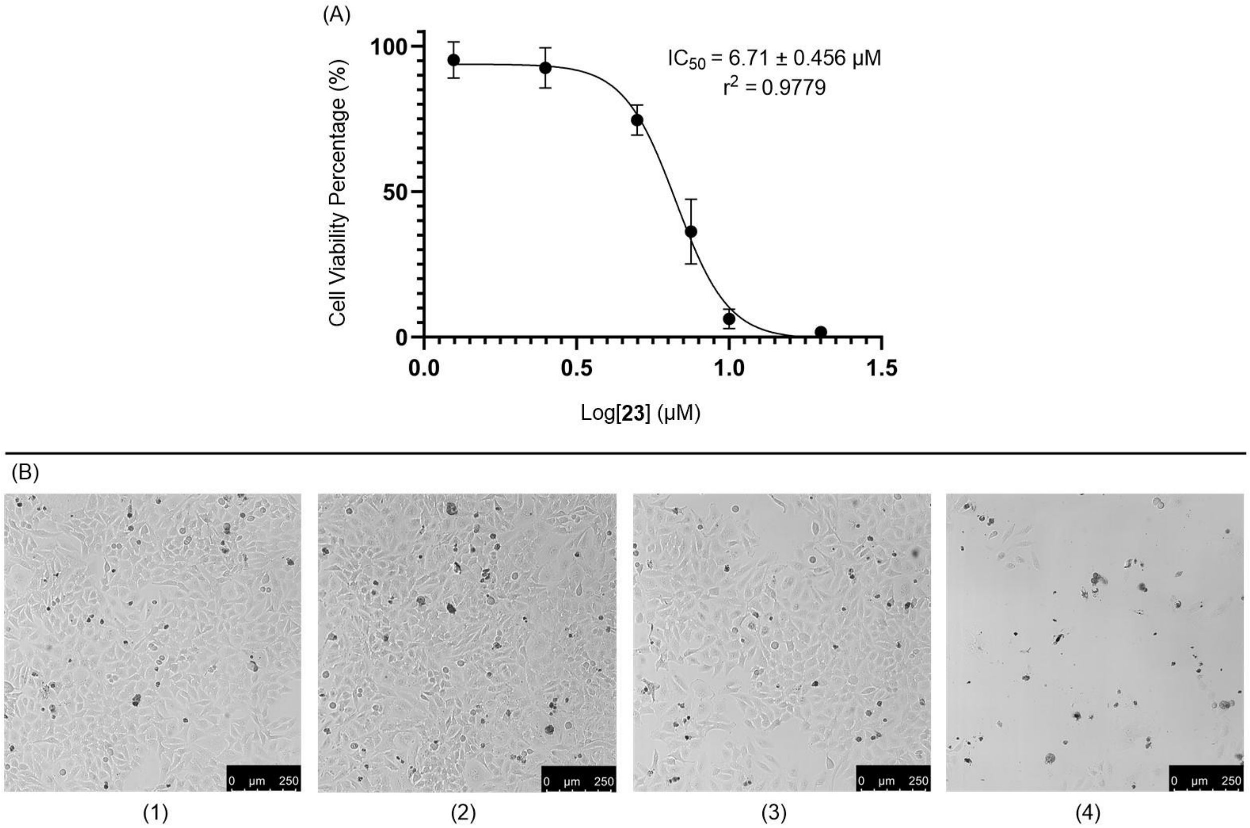

Figure S45. HeLa Cell Viability in the Presence of Diazonium **23**. (A) Cell Viability Dose-Response Curve. (B) Confocal Images of HeLa cells. Image 1: Control plate of HeLa cells incubated with DMEM in the absence of **23**. Plate is nearly completely confluent after 3 days; Image 2: Control plate of HeLa cells incubated with 1x PBS pH 6.6 in the absence of **23**. Plate is nearly completely confluent after 3 days, similar to the plate incubated with DMEM; Image 3: Plate of HeLa cells incubated with 1.25 µM of **23**. Plate is over 90% confluent after 3 days, showing minimal cytotoxicity at this concentration, consistent with the IC_{50} Plot; Image 4: Plate of HeLa cells incubated with 10 µM of **23**. Plate is less than 10% confluent after 3 days, consisting of mainly cellular debris, demonstrating effective cytotoxicity of **23** at this concentration, consistent with the IC_{50} Plot.

XIV. NMR Spectra

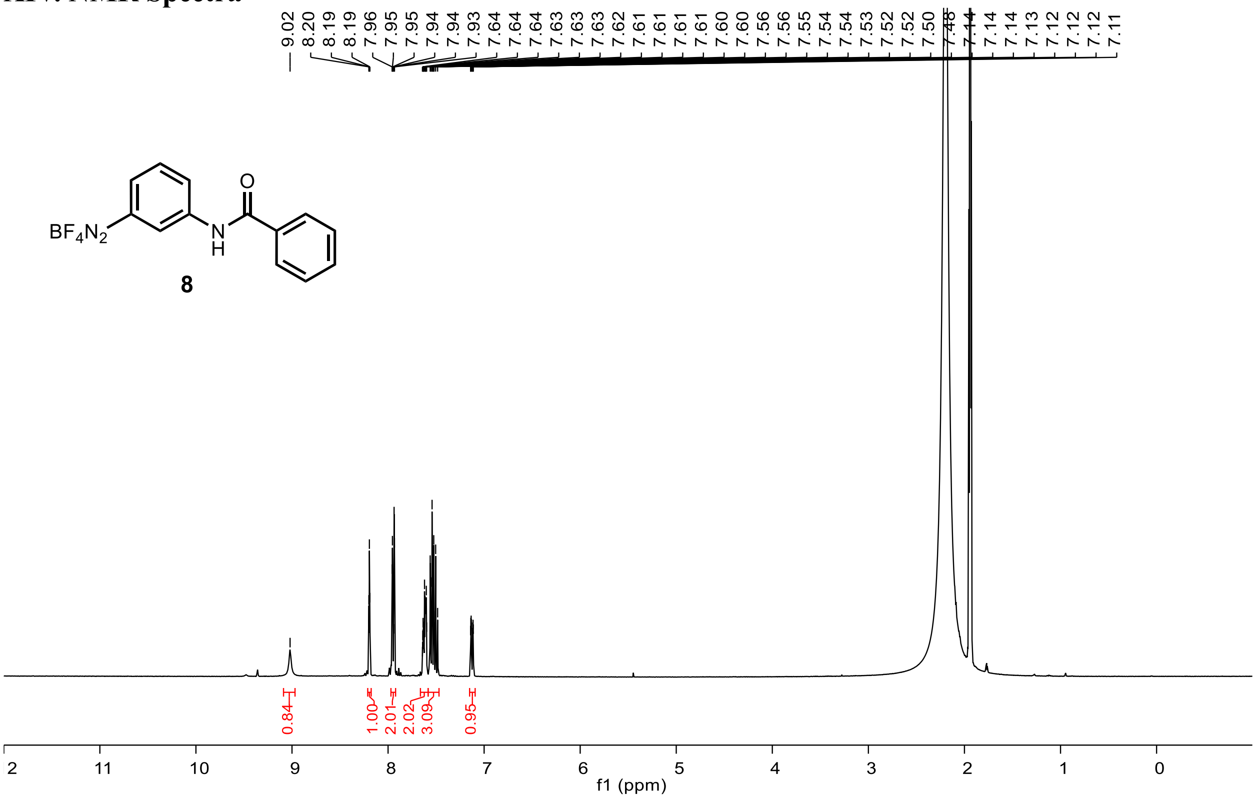

Figure S46a. ^{1}H NMR spectrum (400 MHz, CD_3CN) of **8**

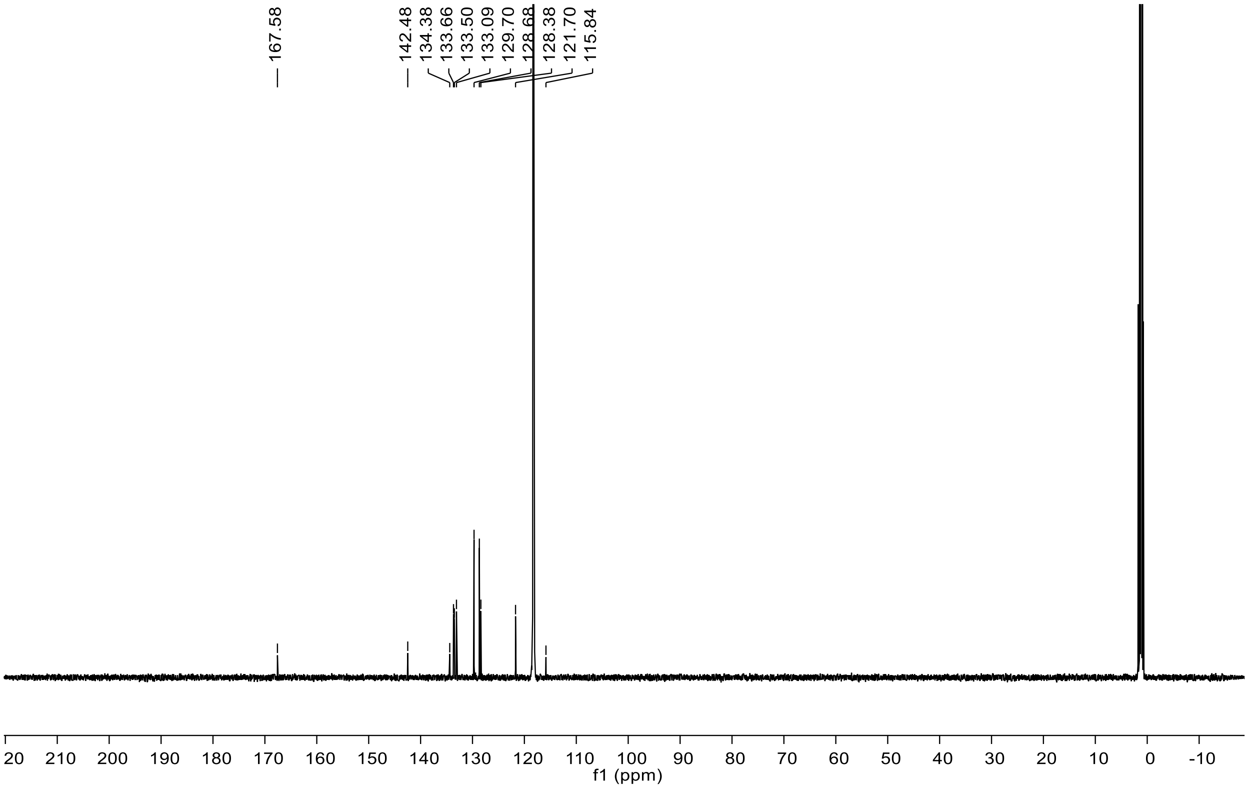

Figure S46b. ^{13}C NMR spectrum (126 MHz, CD_3CN) of **8**

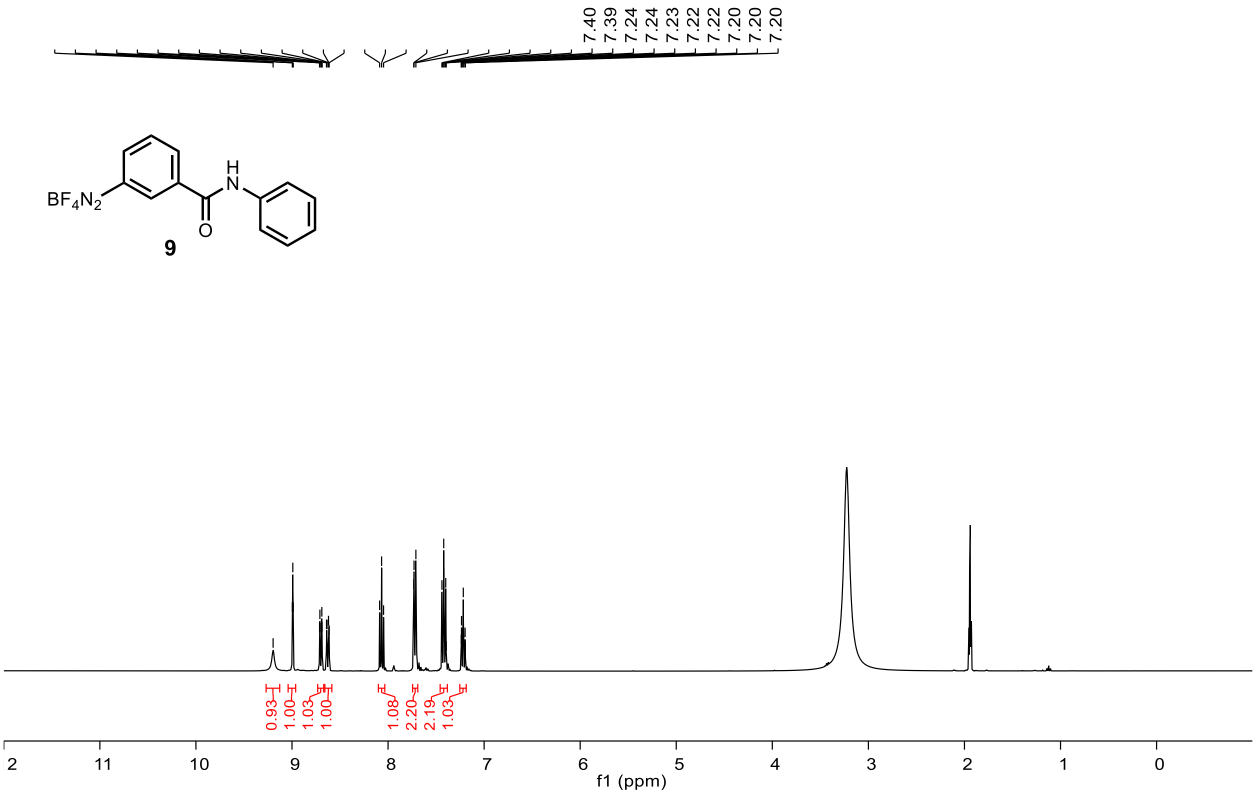

Figure S47a. ^{1}H NMR spectrum (400 MHz, CD_3CN) of **9**

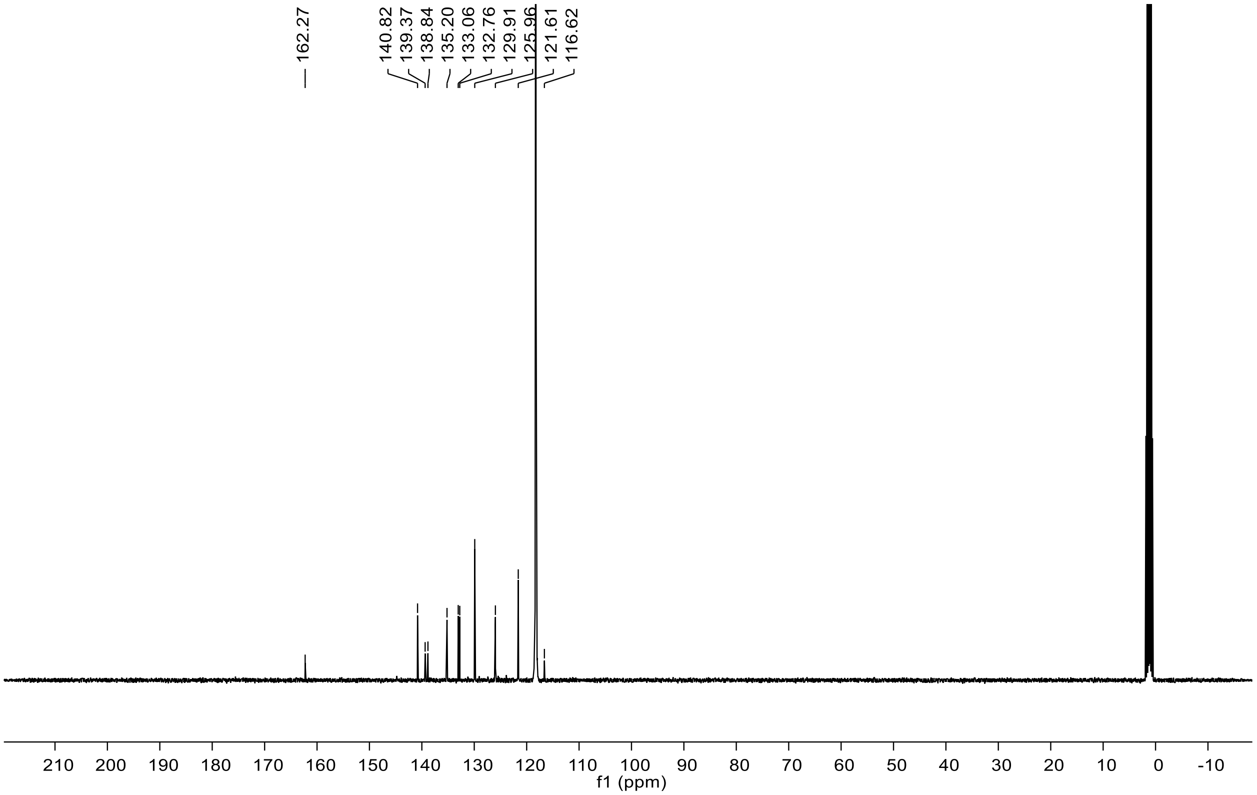

Figure S47b. ^{13}C NMR spectrum (126 MHz, CD_3CN) of **9**

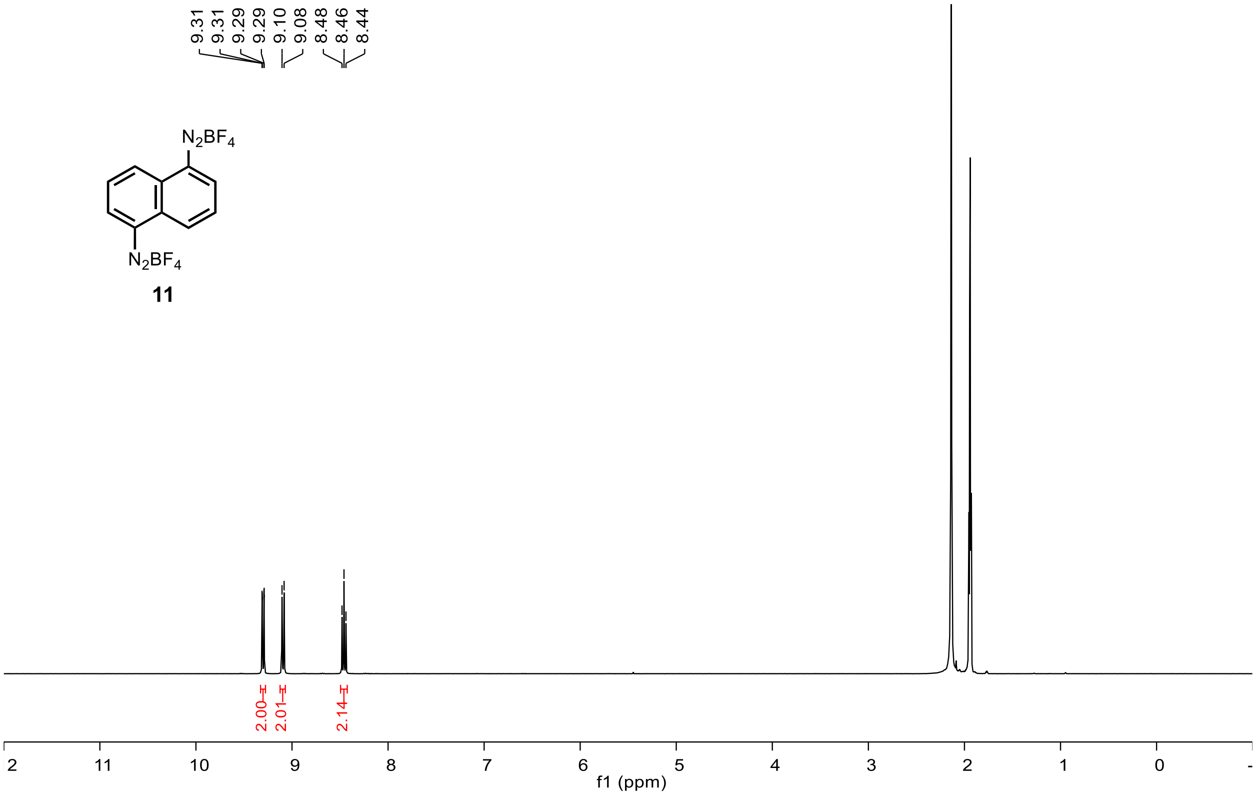

Figure S48a. ^{1}H NMR spectrum (400 MHz, CD_3CN) of **11**

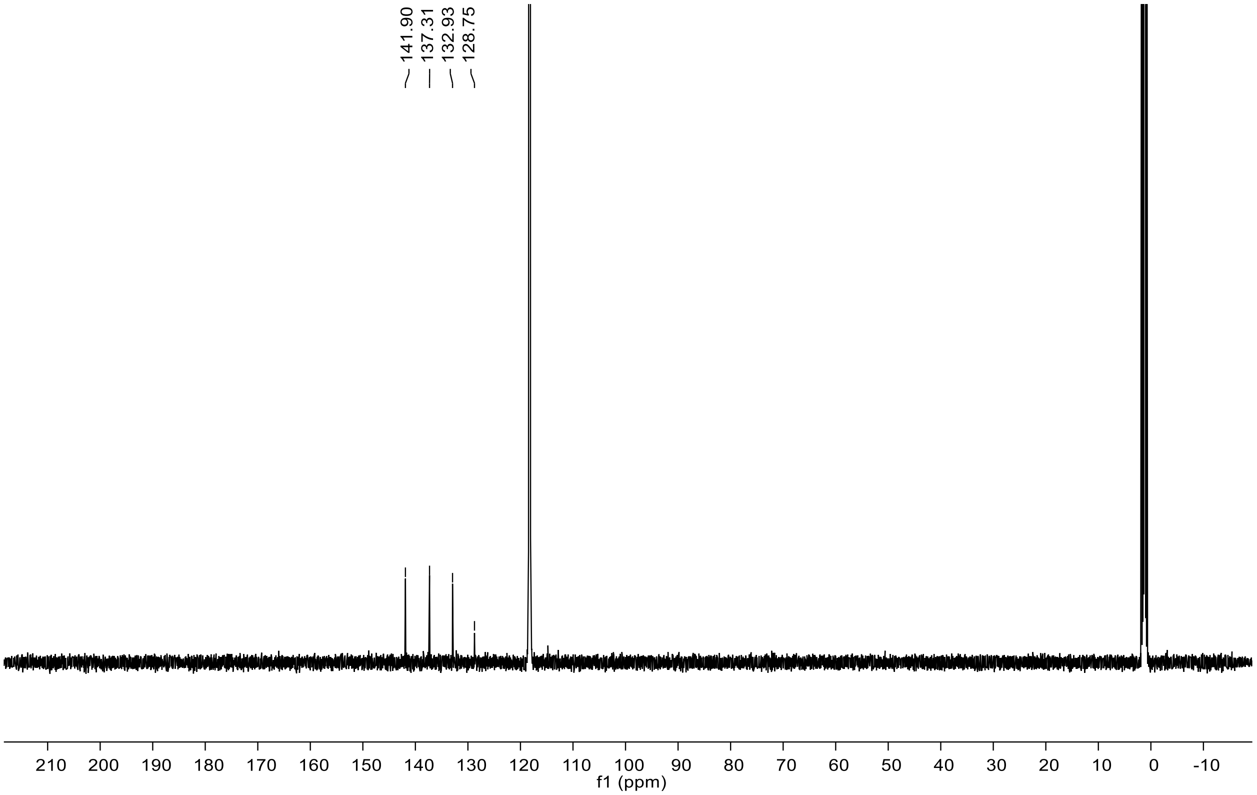

Figure S48b. ^{13}C NMR spectrum (126 MHz, CD_3CN) of **11**

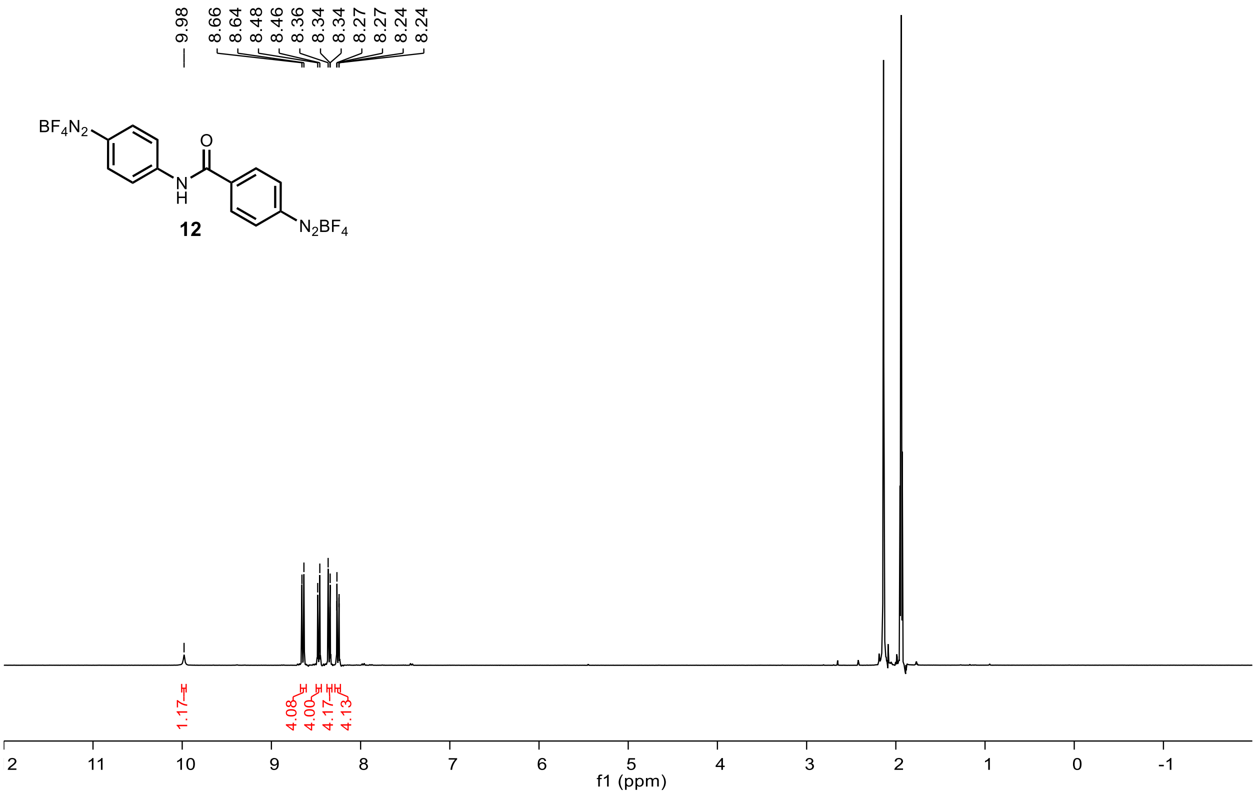

Figure S49a. ^{1}H NMR spectrum (400 MHz, CD_3CN) of **12**

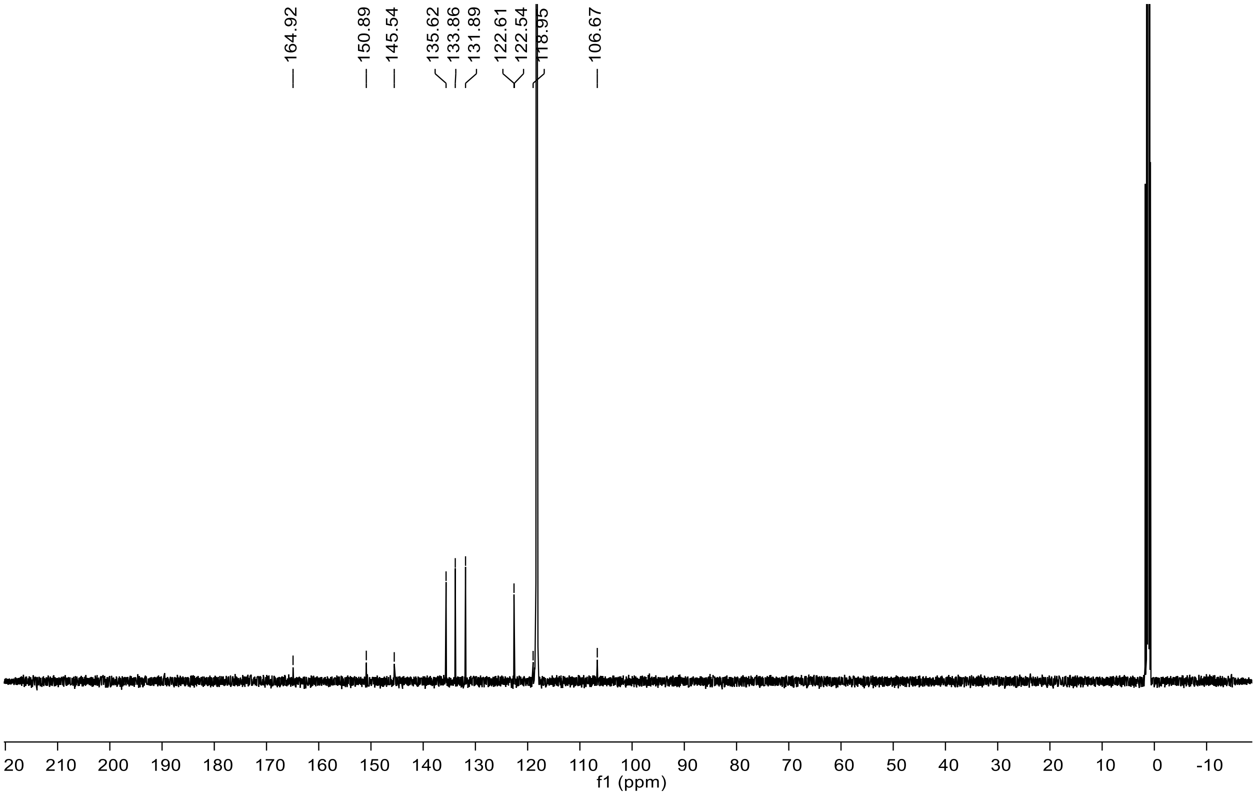

Figure S49b. ^{13}C NMR spectrum (126 MHz, CD_3CN) of **12**

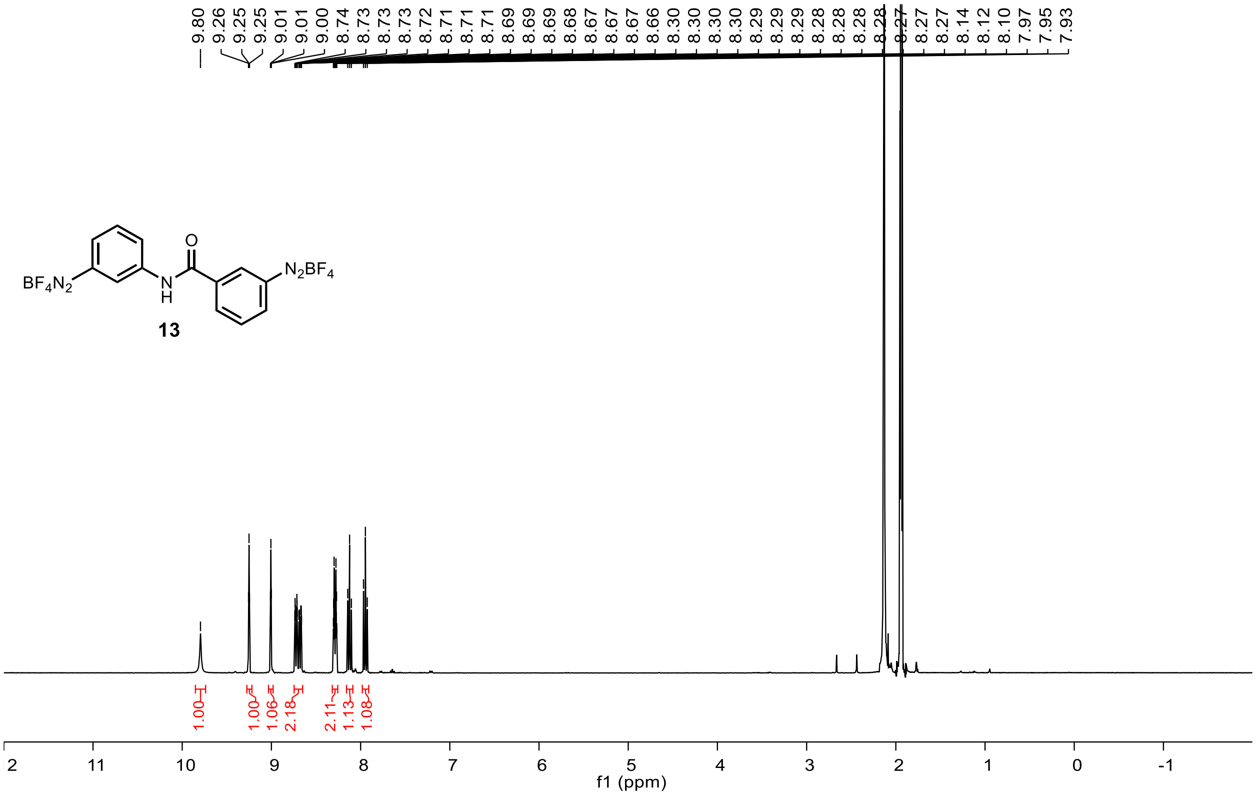

Figure S50a. ^{1}H NMR spectrum (500 MHz, CD_3CN) of **13**

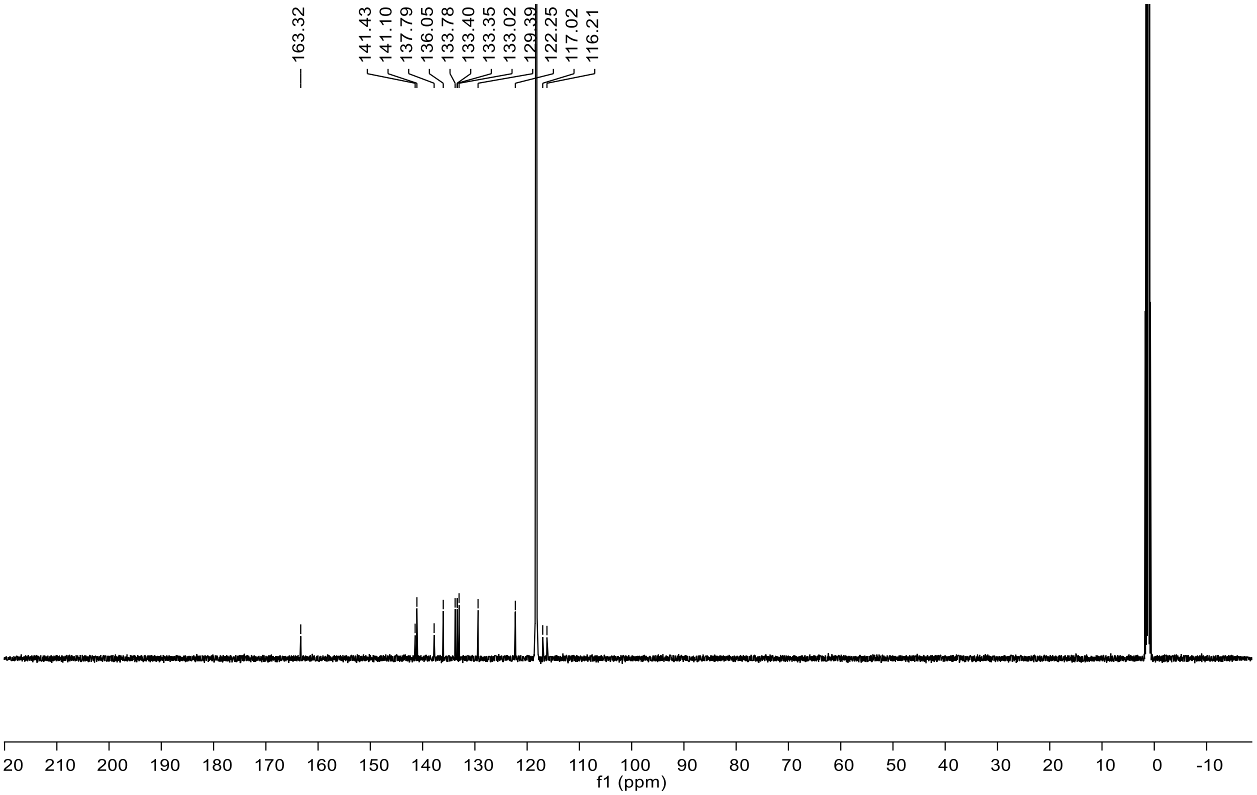

Figure S50b. ^{13}C NMR spectrum (126 MHz, CD_3CN) of **13**

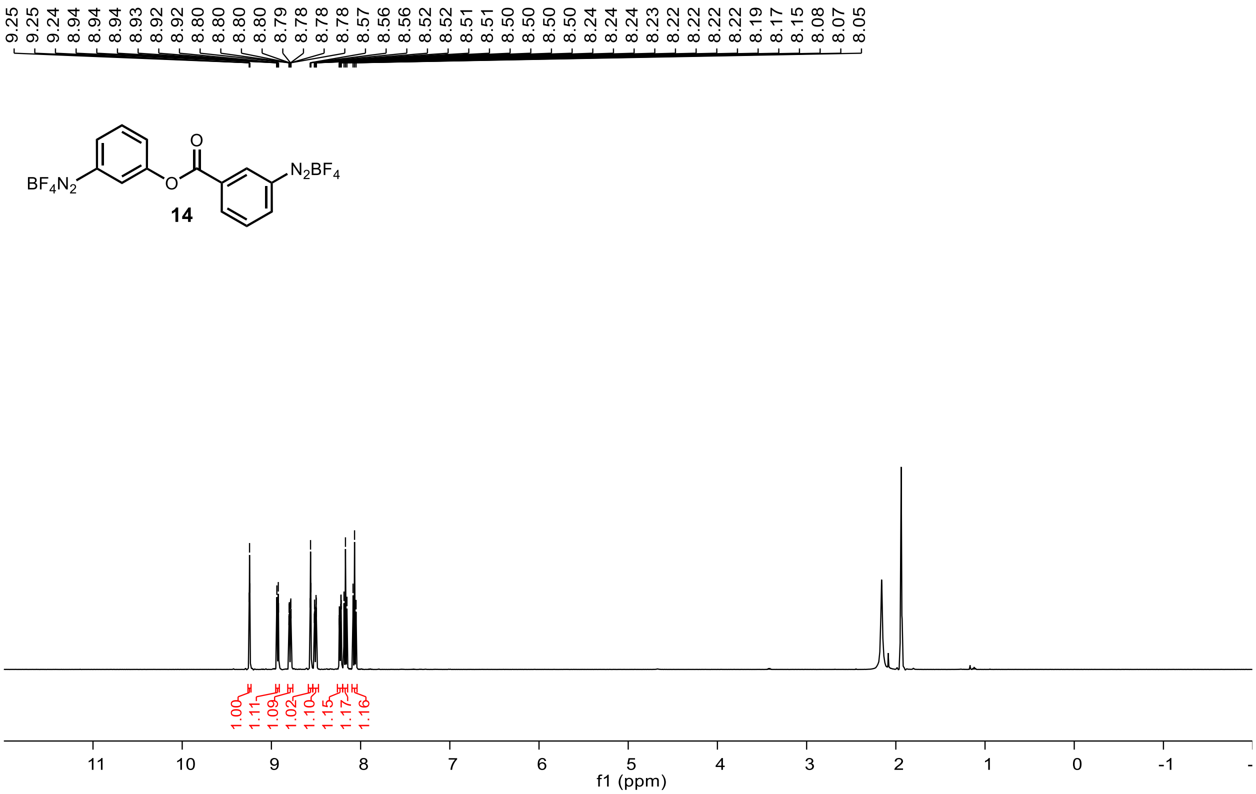

Figure S51a. ^{1}H NMR spectrum (400 MHz, CD_3CN) of **14**

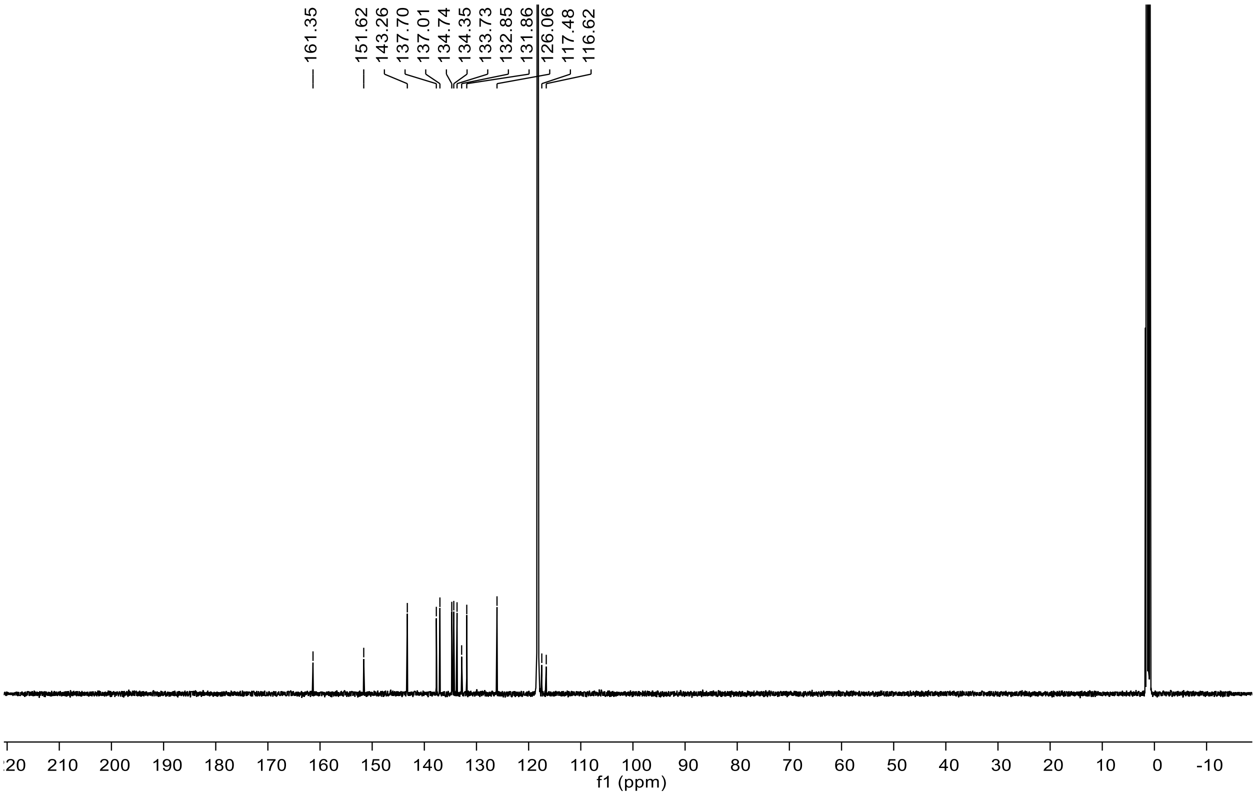

Figure S51b. ^{13}C NMR spectrum (126 MHz, CD_3CN) of **14**

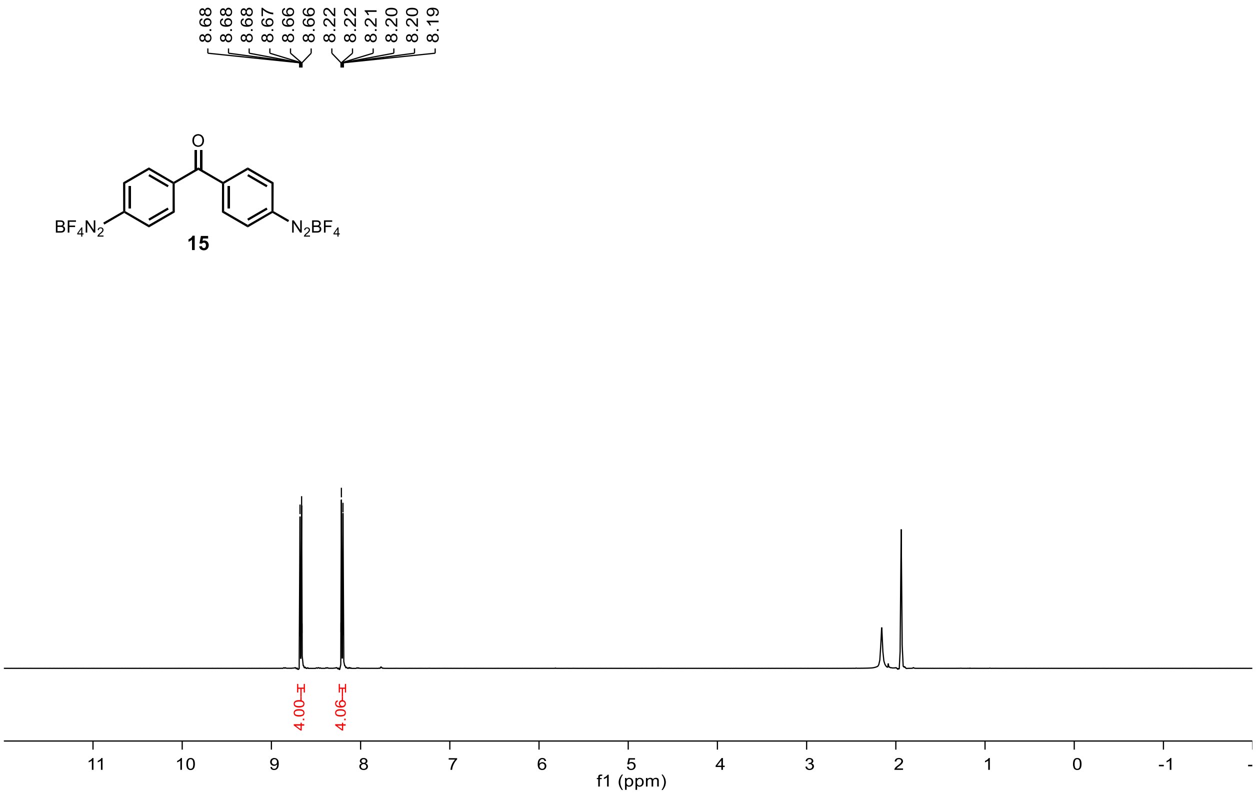

Figure S52a. ^{1}H NMR spectrum (400 MHz, CD_3CN) of **15**

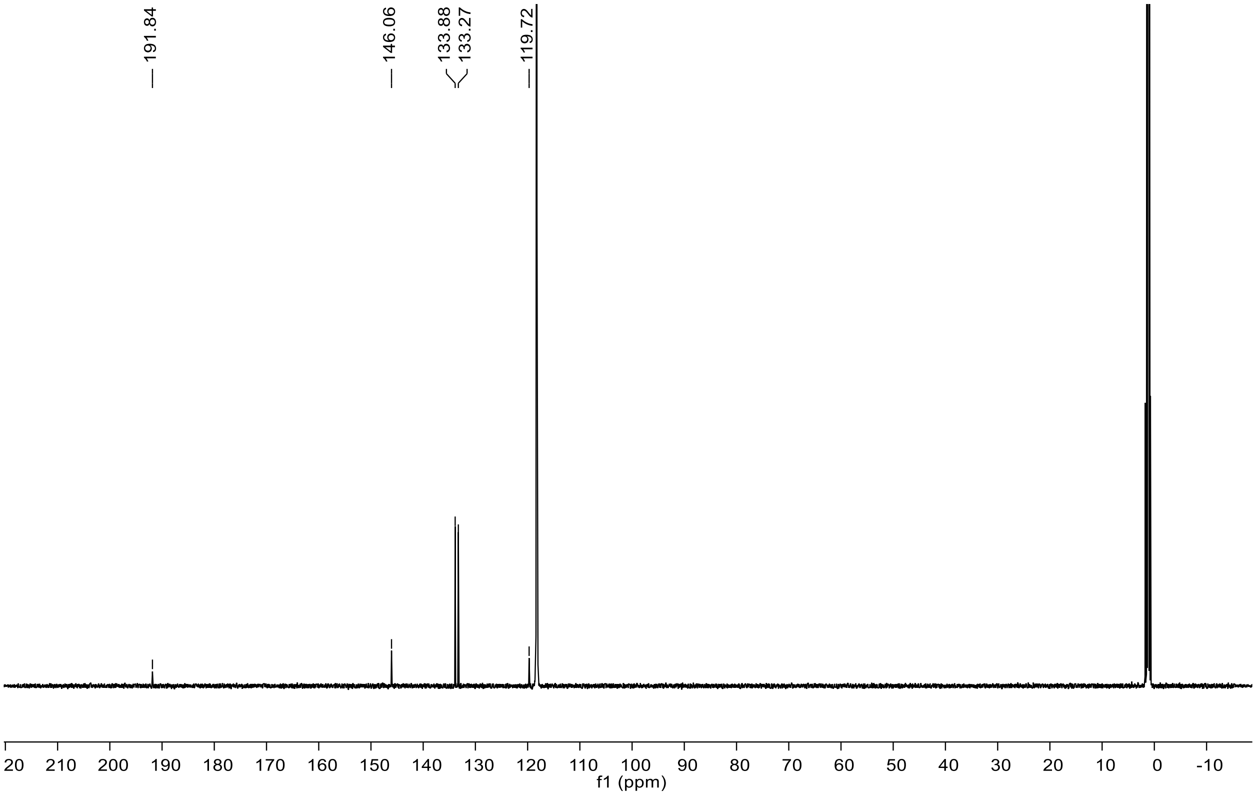

Figure S52b. ^{13}C NMR spectrum (126 MHz, CD_3CN) of **15**

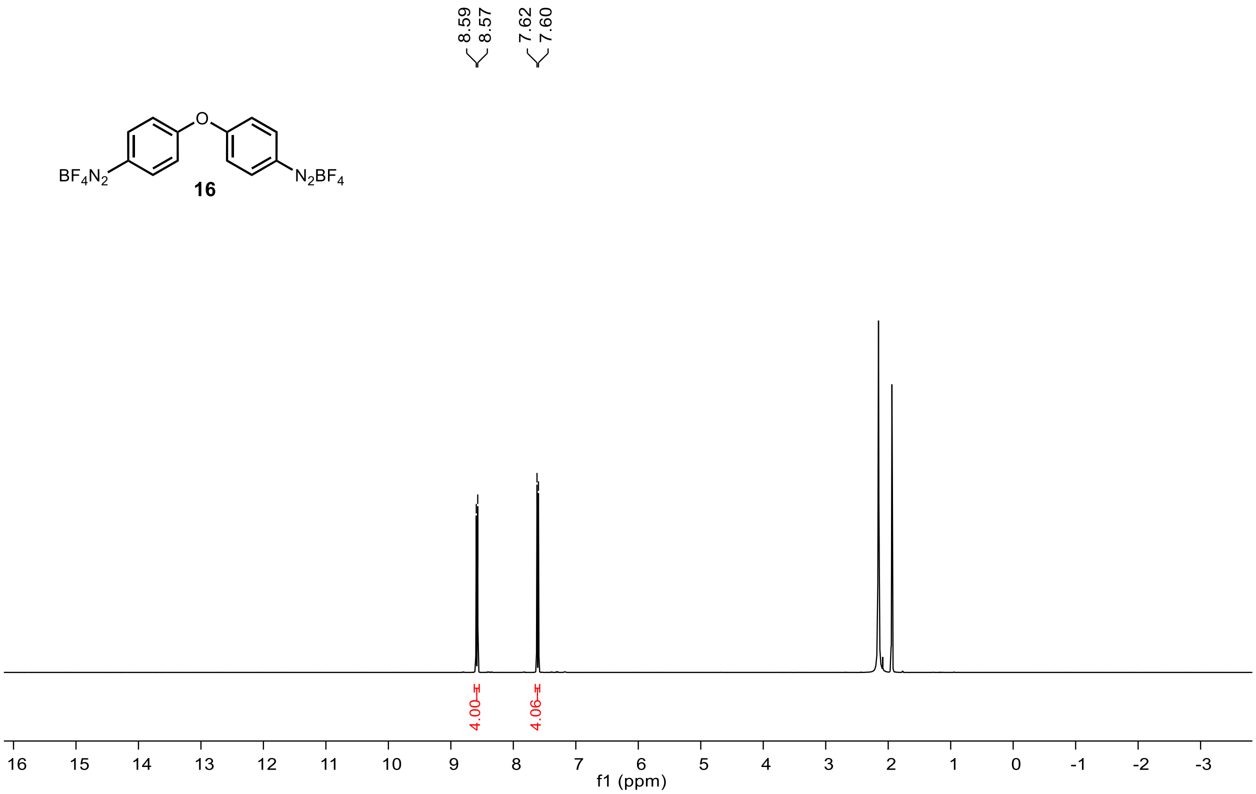

Figure S53a. ^{1}H NMR spectrum (400 MHz, CD_3CN) of **16**

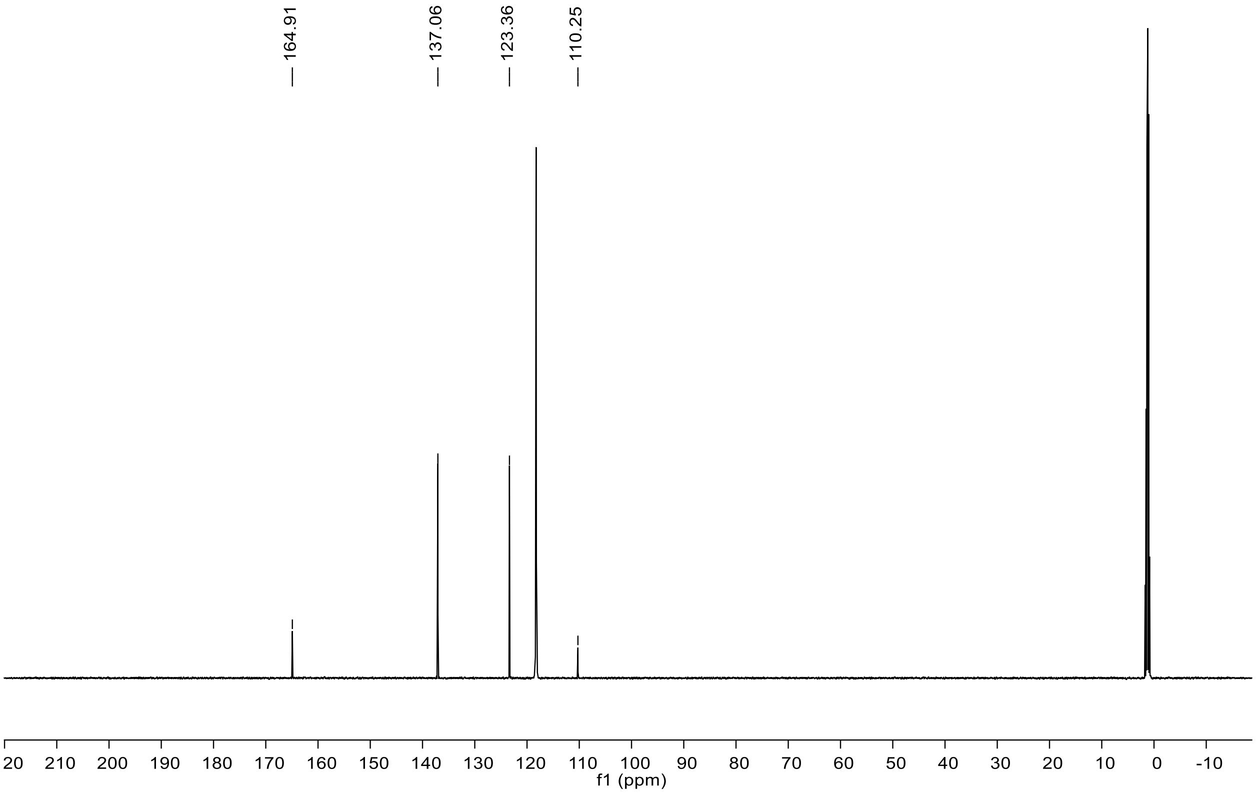

Figure S53b. ^{13}C NMR spectrum (126 MHz, CD_3CN) of **16**

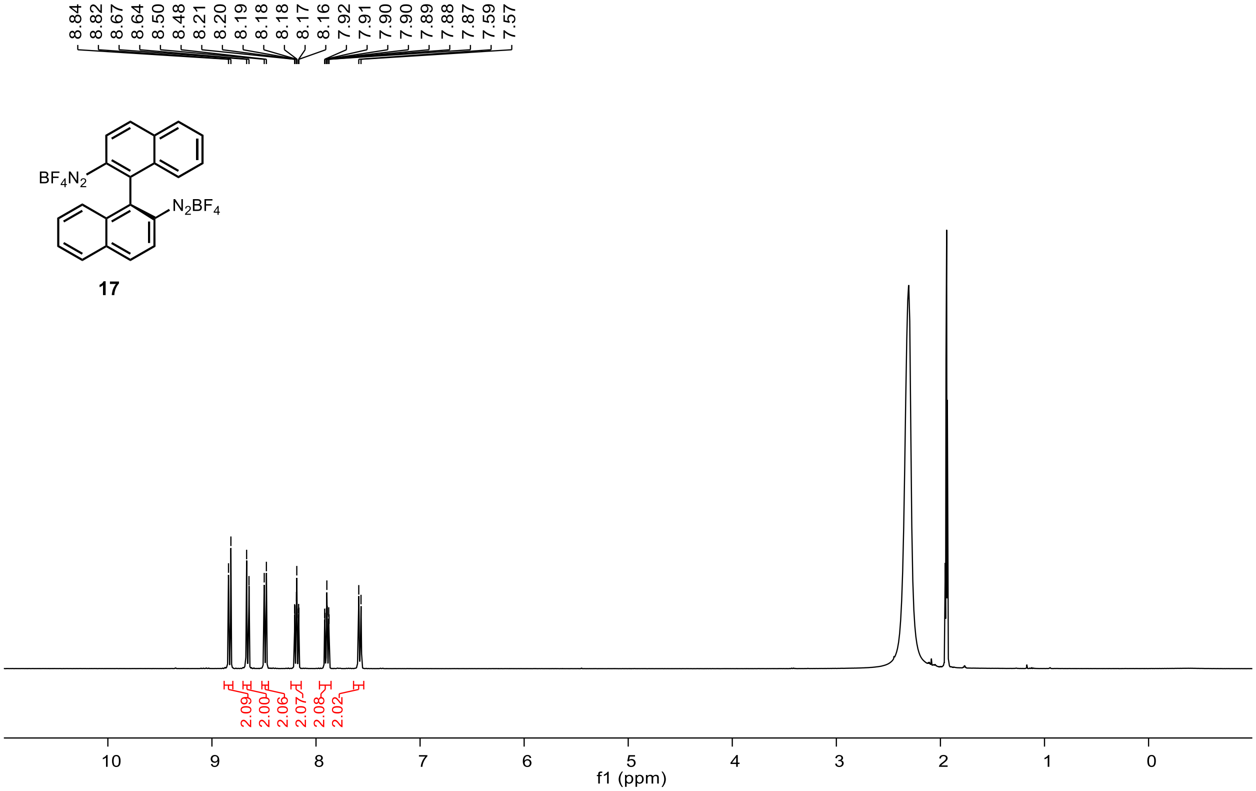

Figure S54a. ^{1}H NMR spectrum (400 MHz, CD_3CN) of **17**

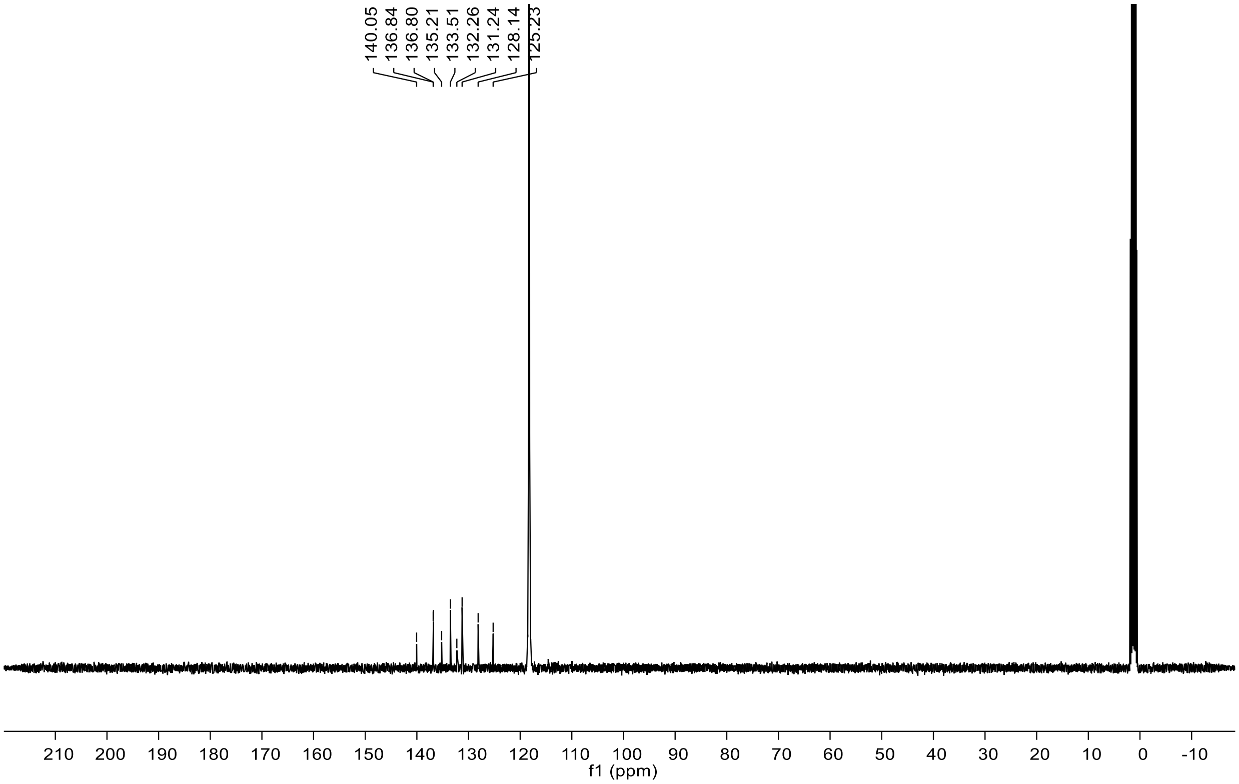

Figure S54b. ^{13}C NMR spectrum (126 MHz, CD_3CN) of **17**

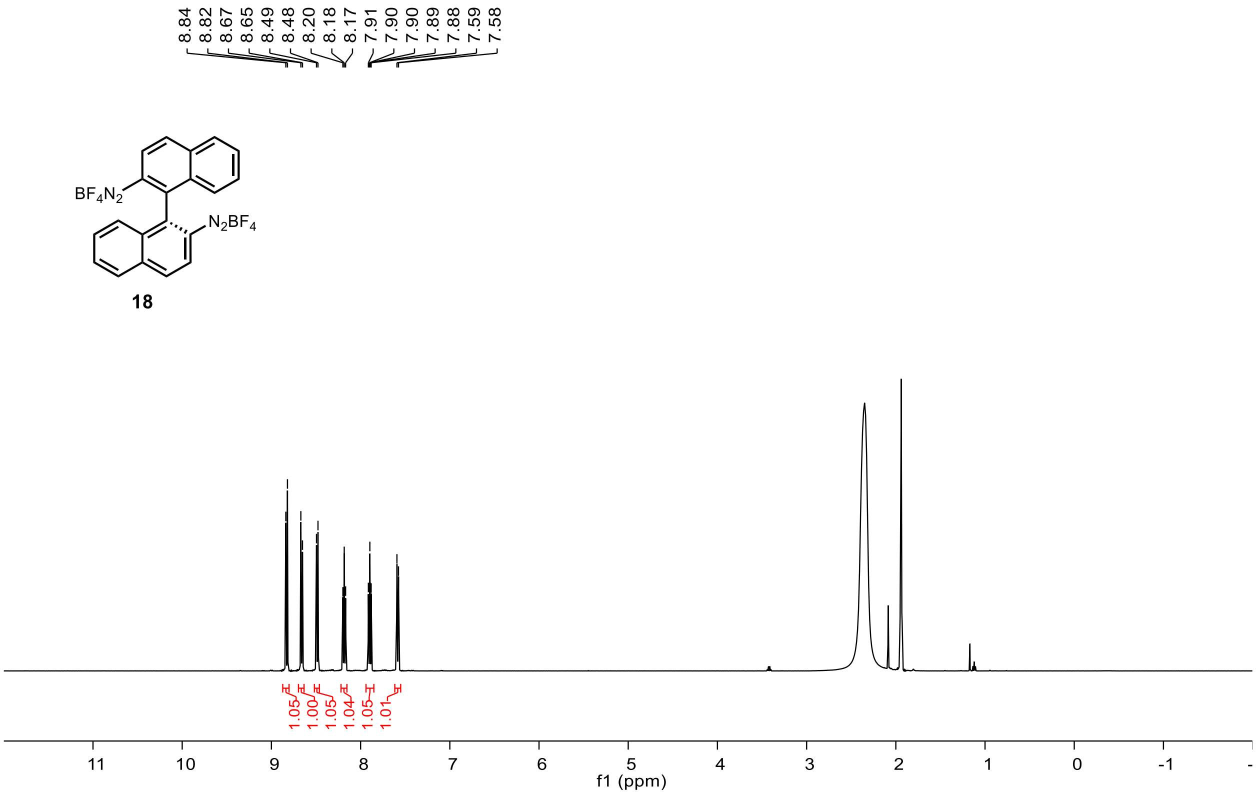

Figure S55a. ^{1}H NMR spectrum (500 MHz, CD_3CN) of **18**

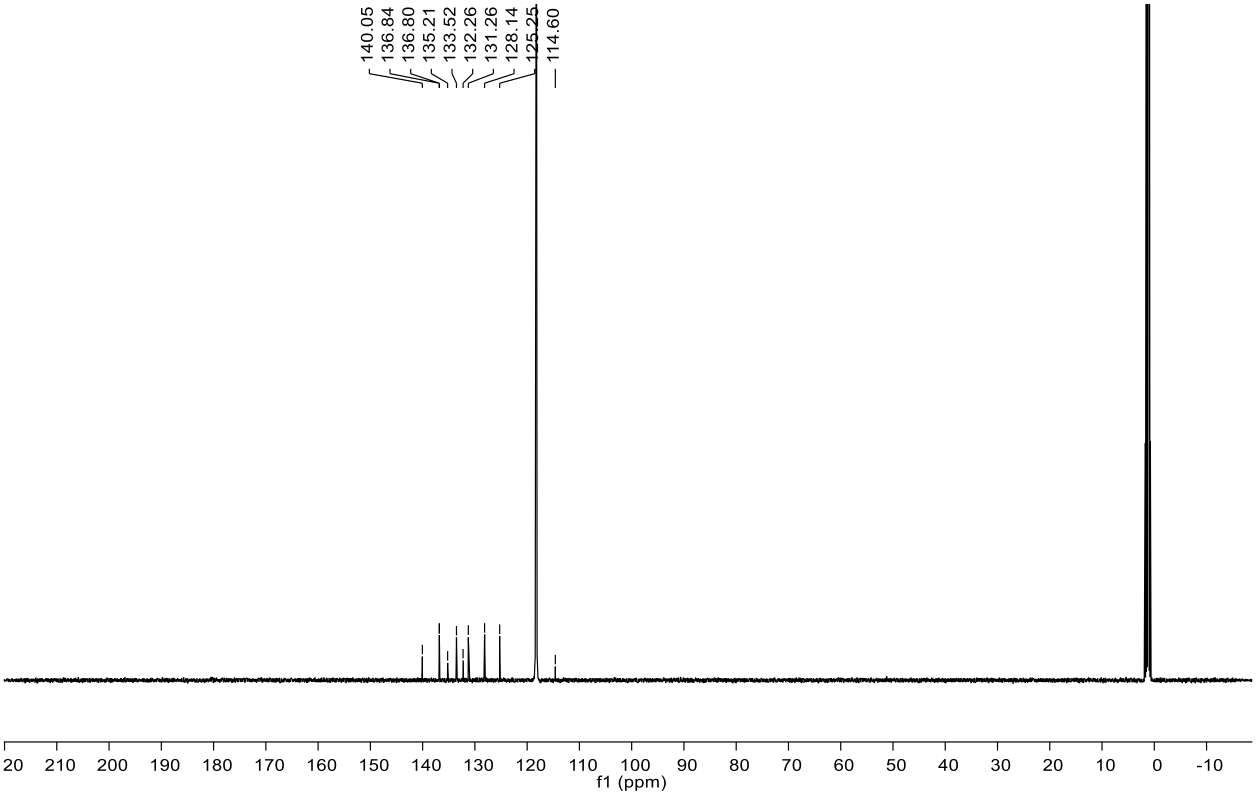

Figure S55b. ^{13}C NMR spectrum (126 MHz, CD_3CN) of **18**

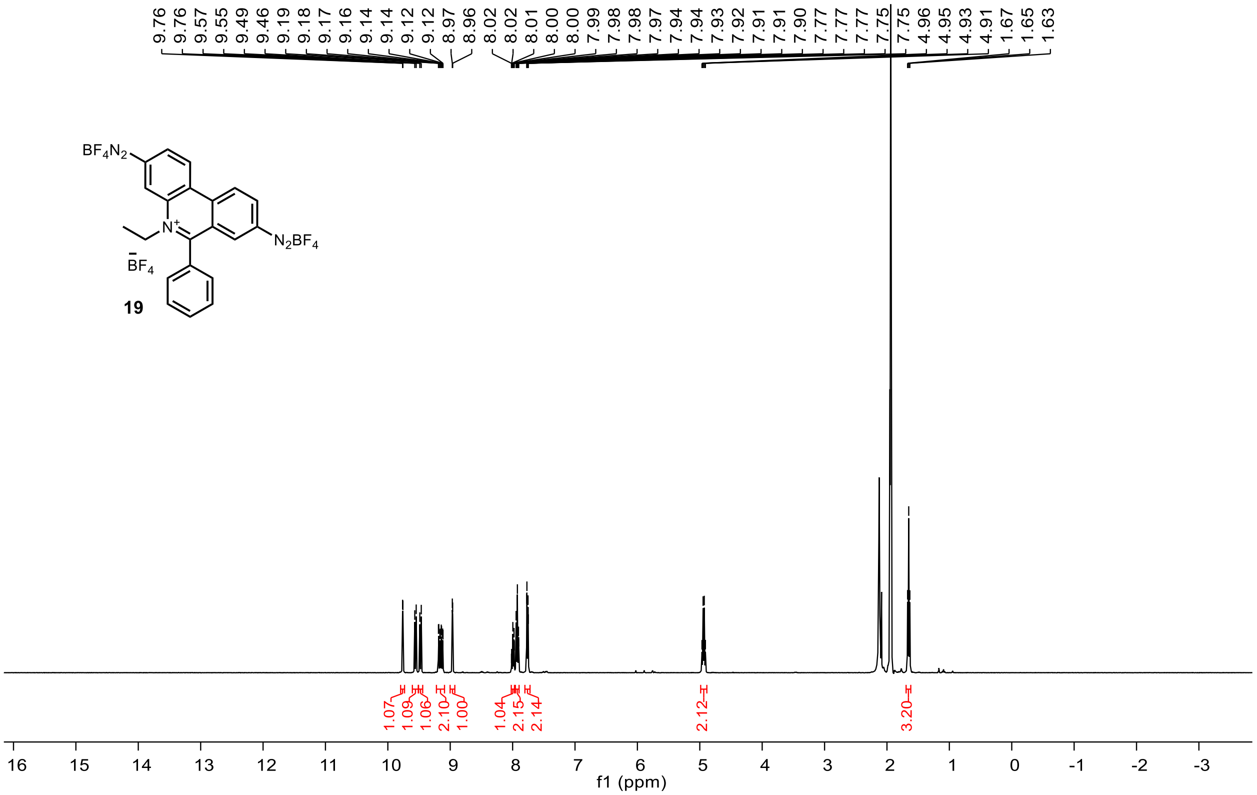

Figure S56a. ^{1}H NMR spectrum (400 MHz, CD_3CN) of **19**

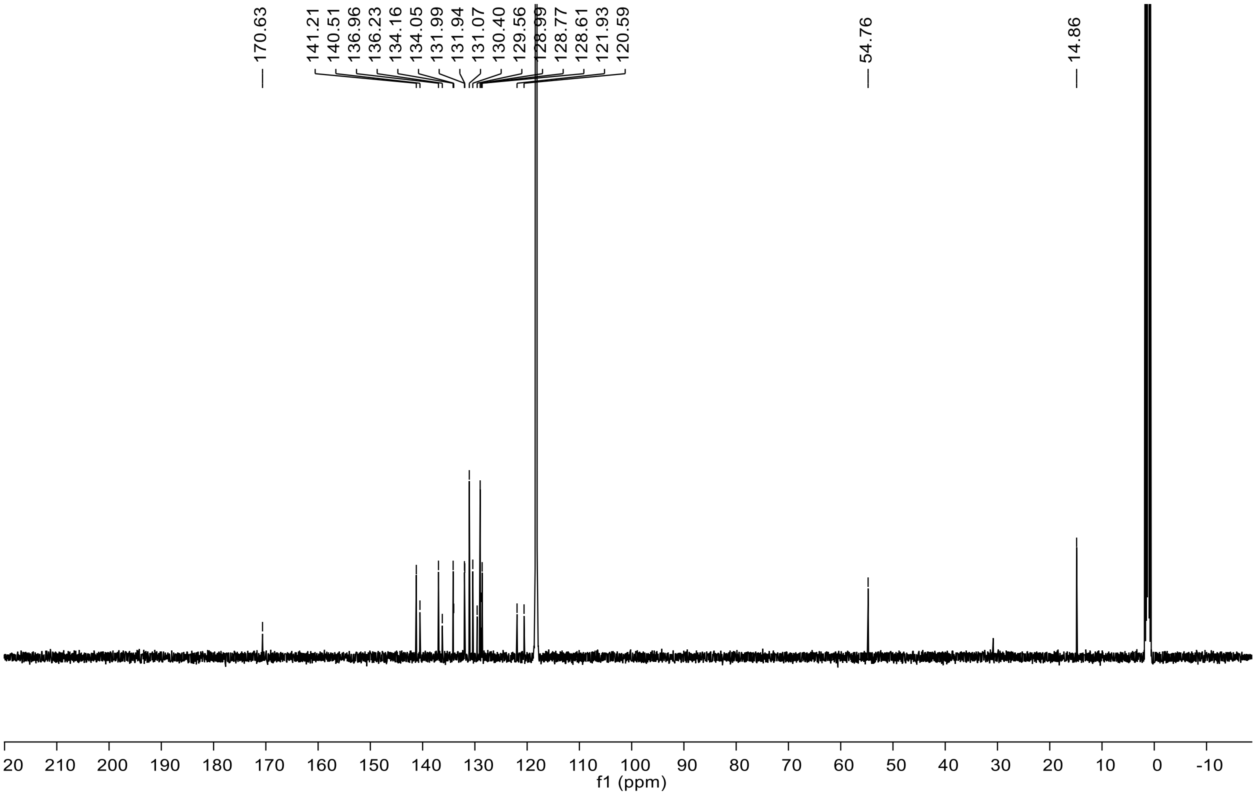

Figure S56b. ^{13}C NMR spectrum (126 MHz, CD_3CN) of **19**

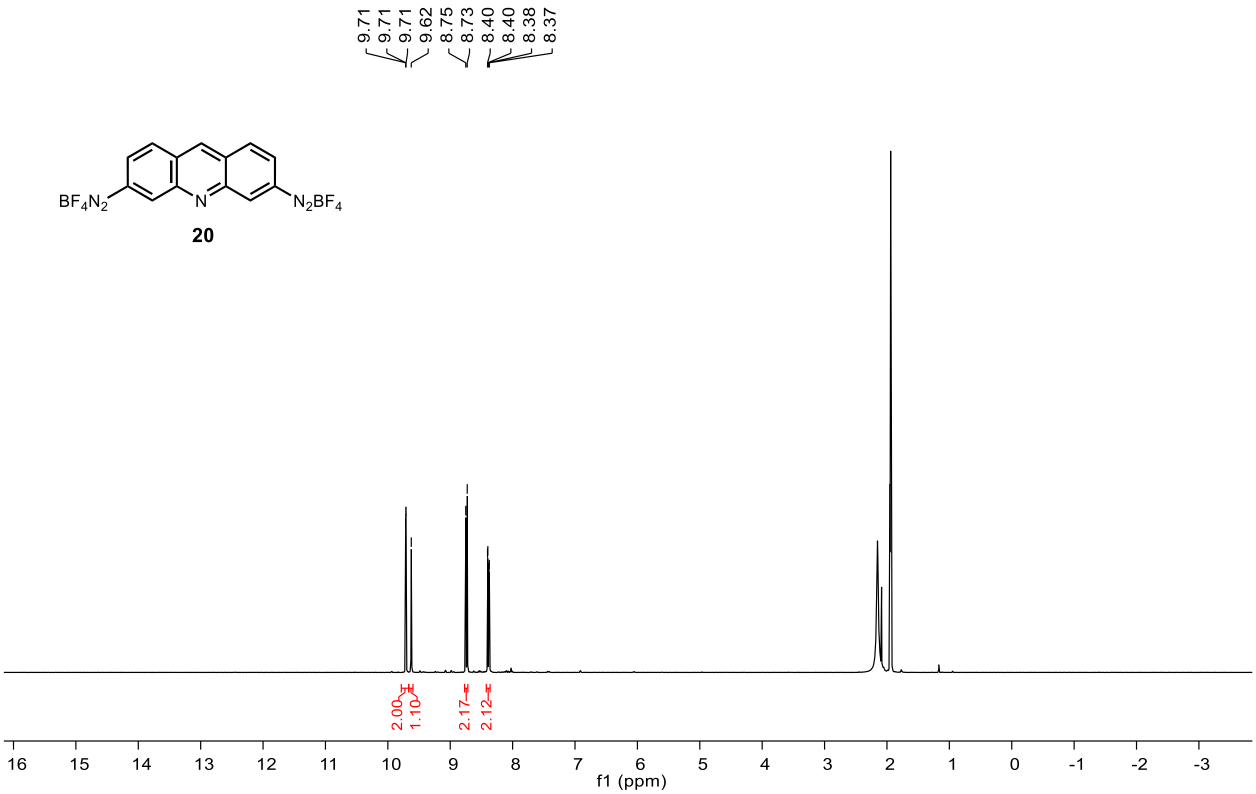

Figure S57a. ^{1}H NMR spectrum (400 MHz, CD_3CN) of **20**

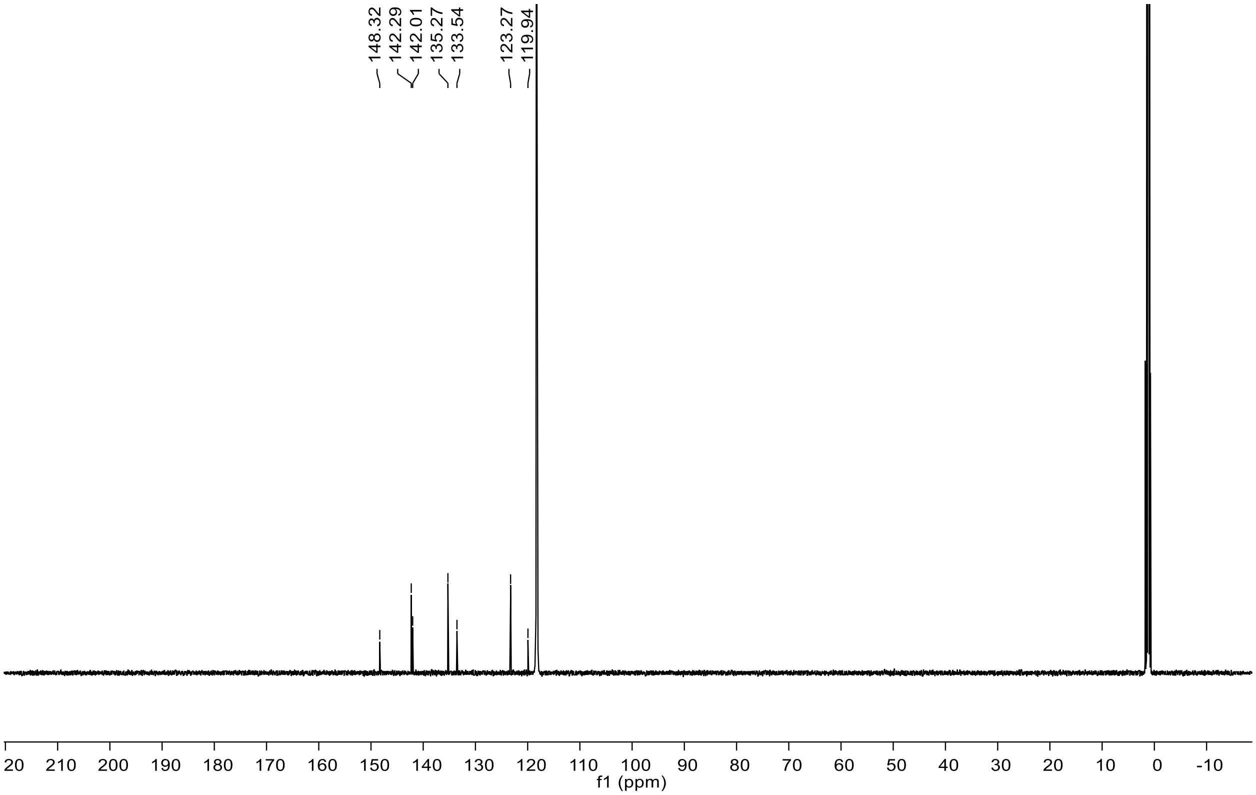

Figure S57b. ^{13}C NMR spectrum (126 MHz, CD_3CN) of **20**

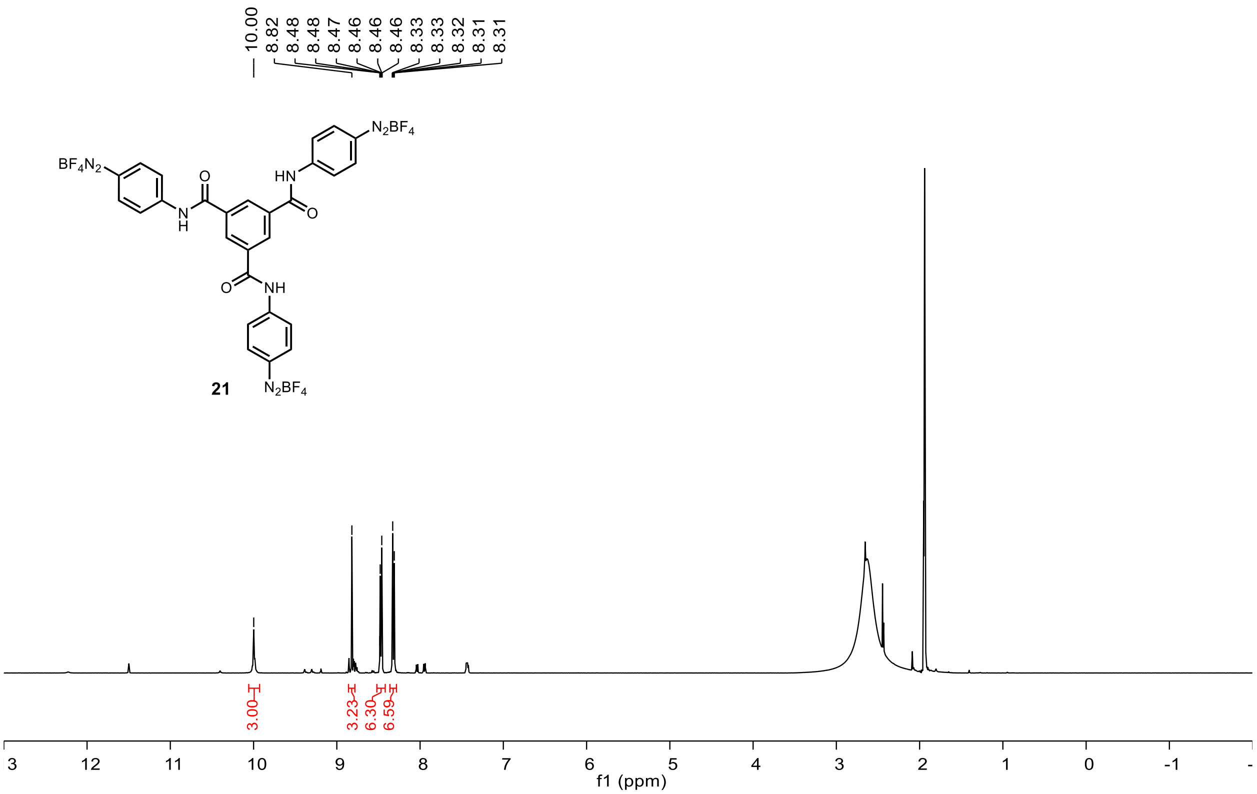

Figure S58a. ^{1}H NMR spectrum (400 MHz, CD_3CN) of **21**

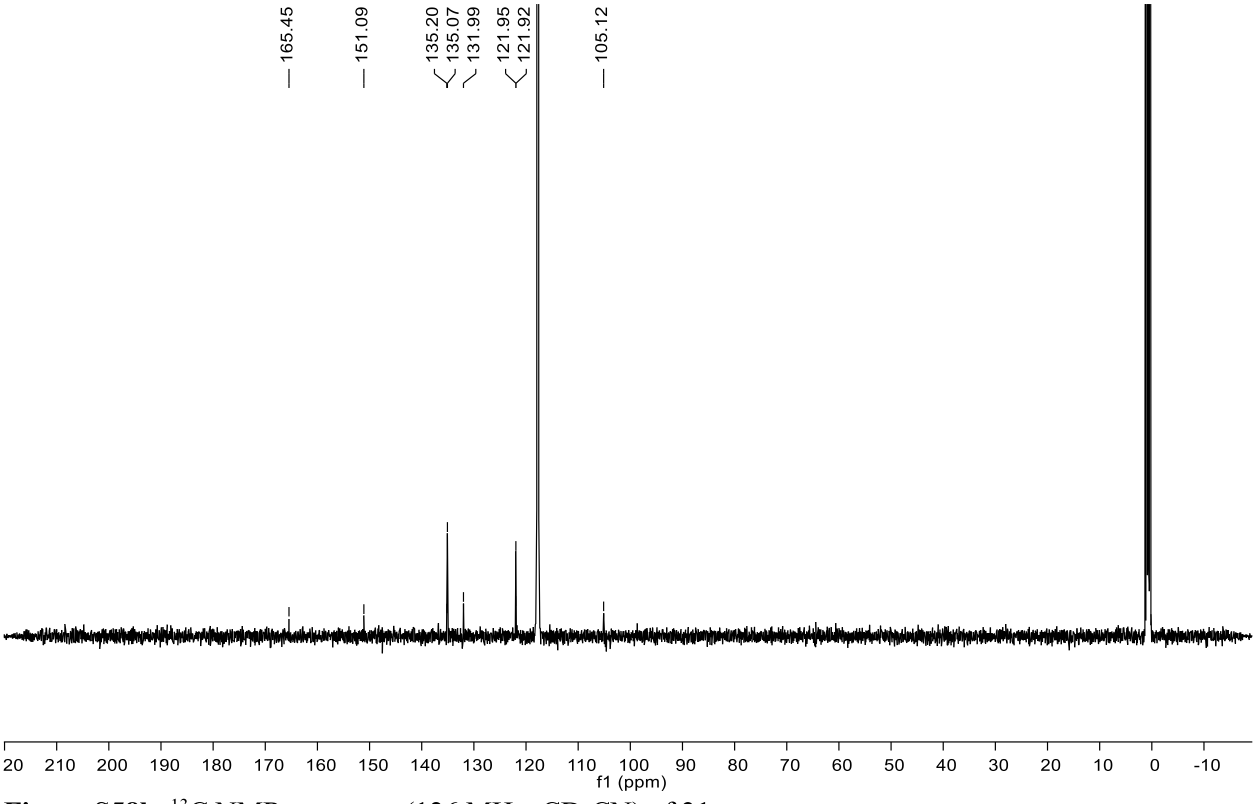

Figure S58b. ^{13}C NMR spectrum (126 MHz, CD_3CN) of **21**

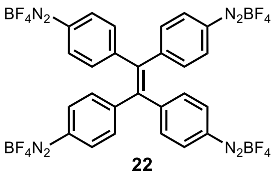

22

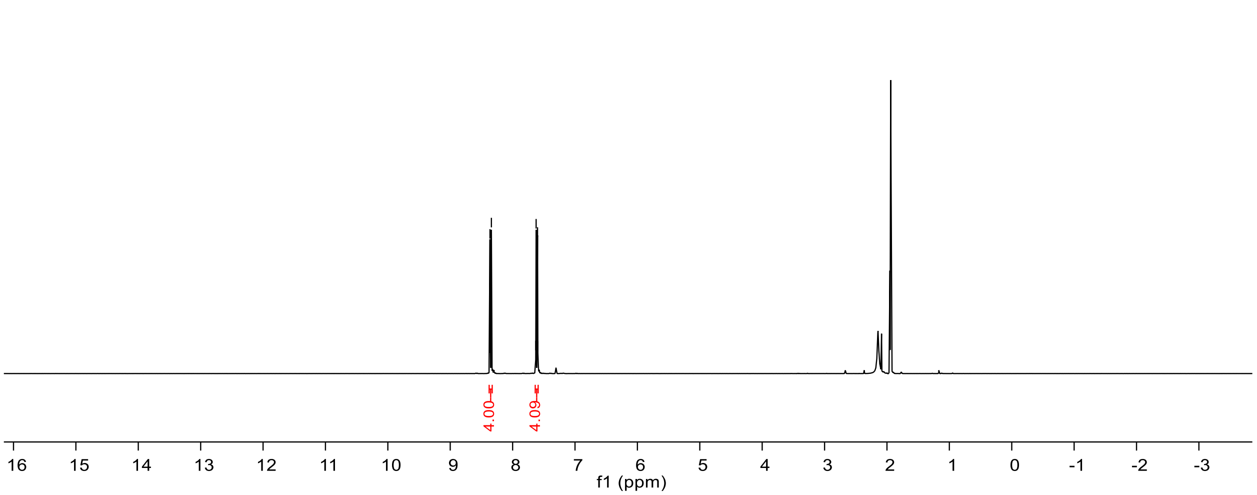

Figure S59a. ^{1}H NMR spectrum (400 MHz, CD_3CN) of **22**

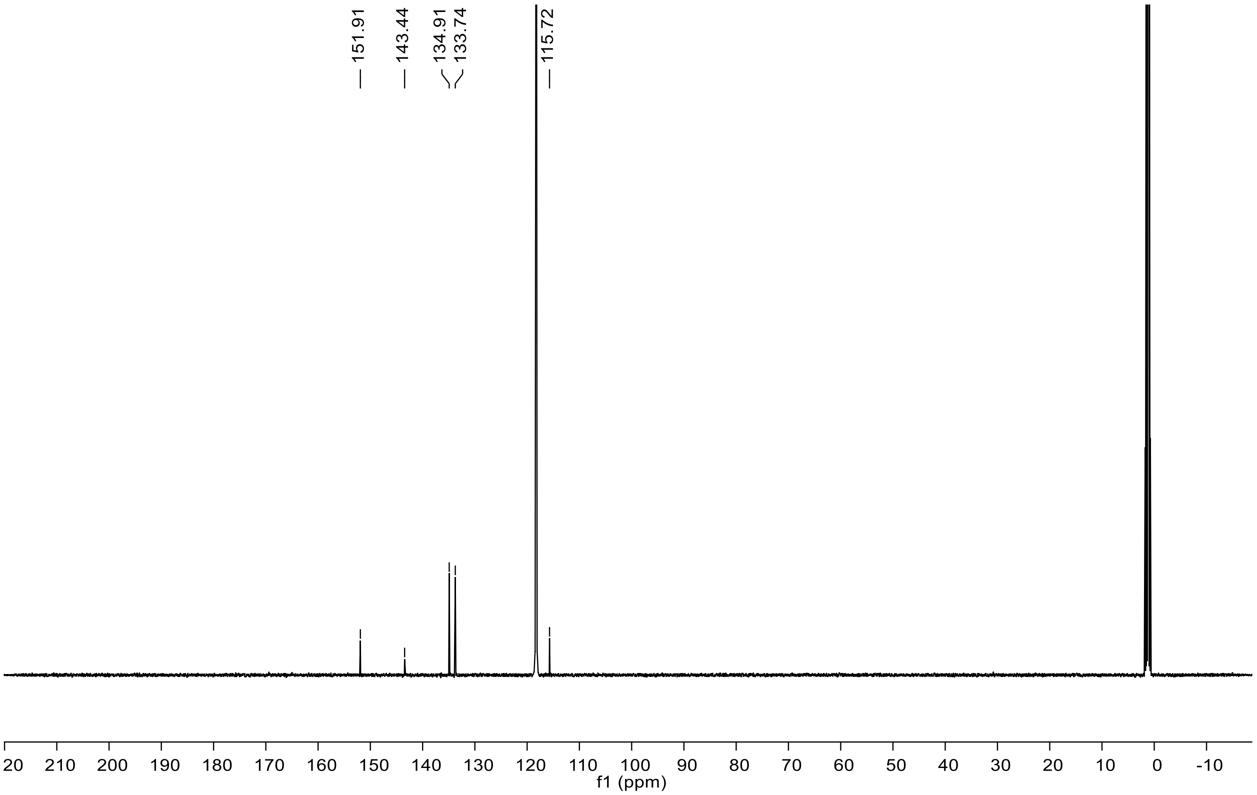

Figure S59b. ^{13}C NMR spectrum (126 MHz, CD_3CN) of **22**

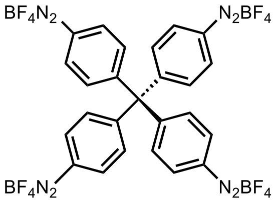

23

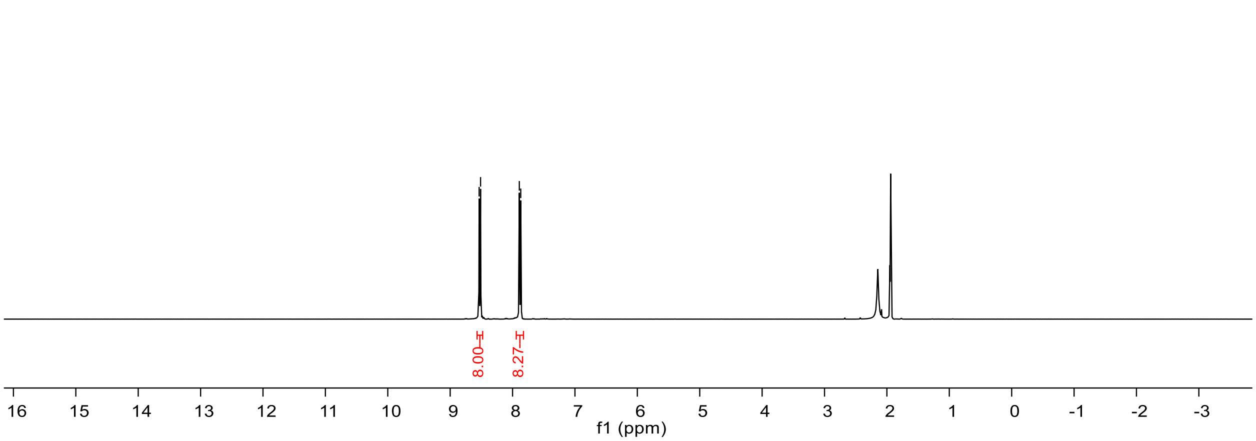

Figure S60a. ^{1}H NMR spectrum (400 MHz, CD_3CN) of **23**

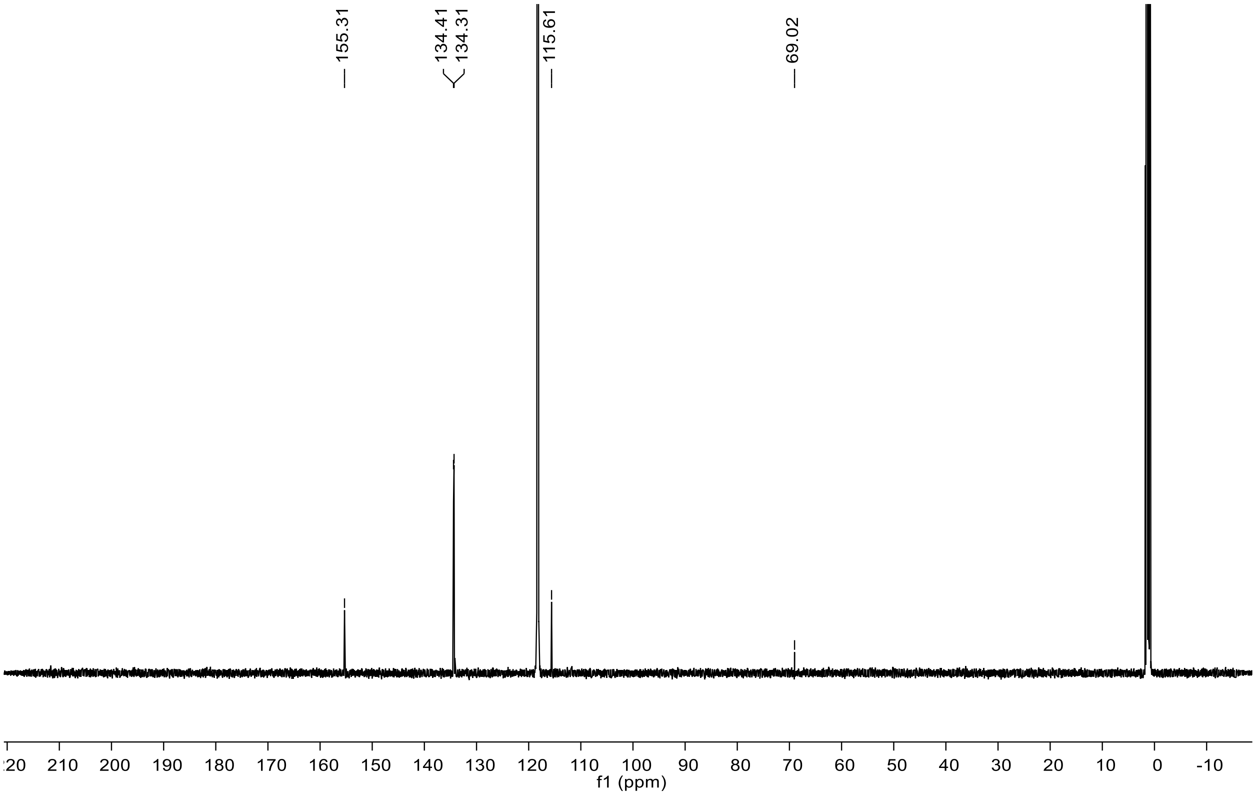

Figure S60b. ^{13}C NMR spectrum (126 MHz, CD_3CN) of **23**

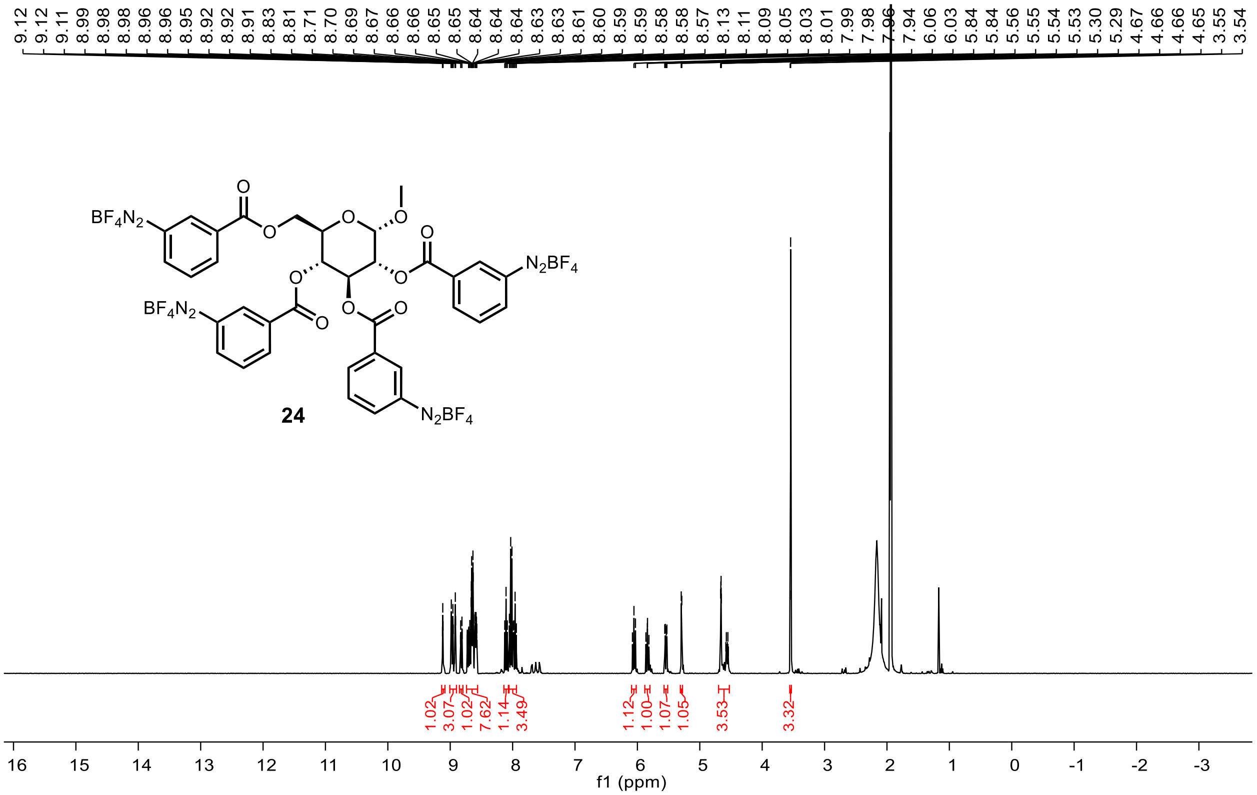

Figure S61a. ^{1}H NMR spectrum (400 MHz, CD_3CN) of **24**

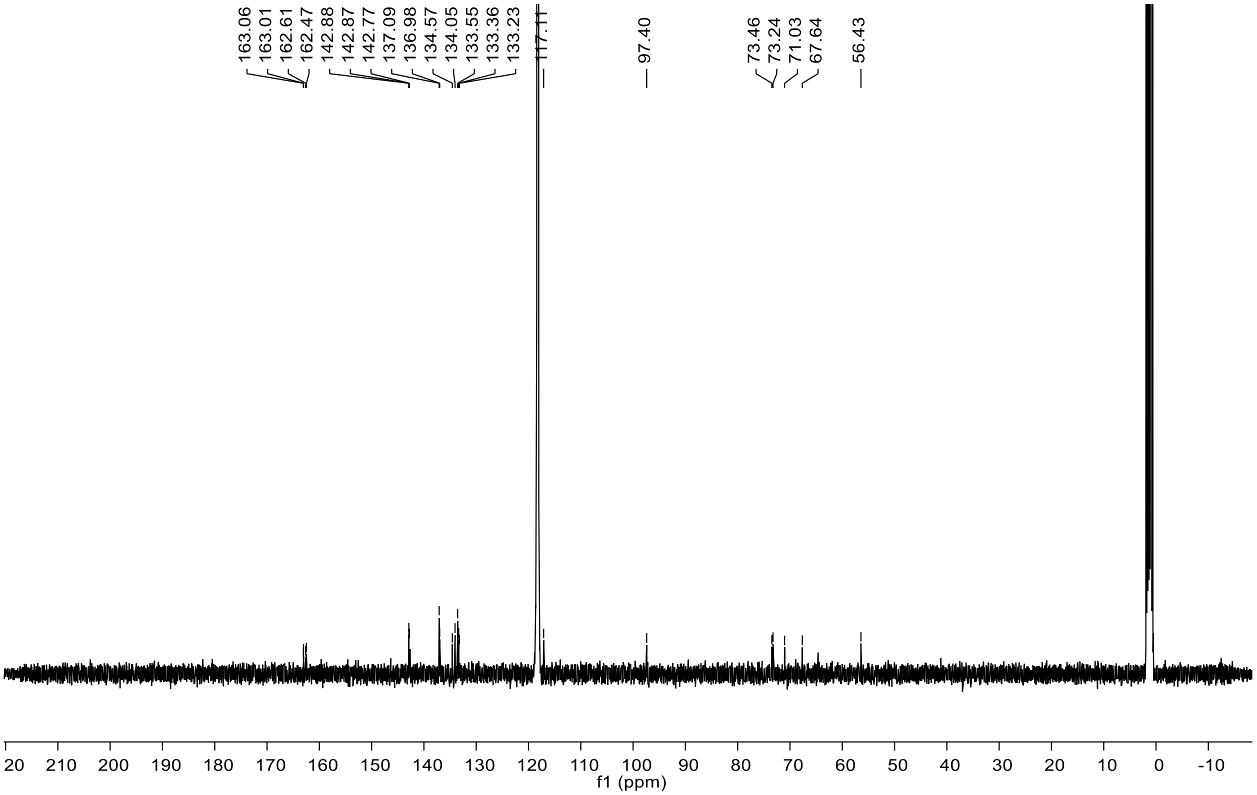

Figure S61b. ^{13}C NMR spectrum (126 MHz, CD_3CN) of **24**

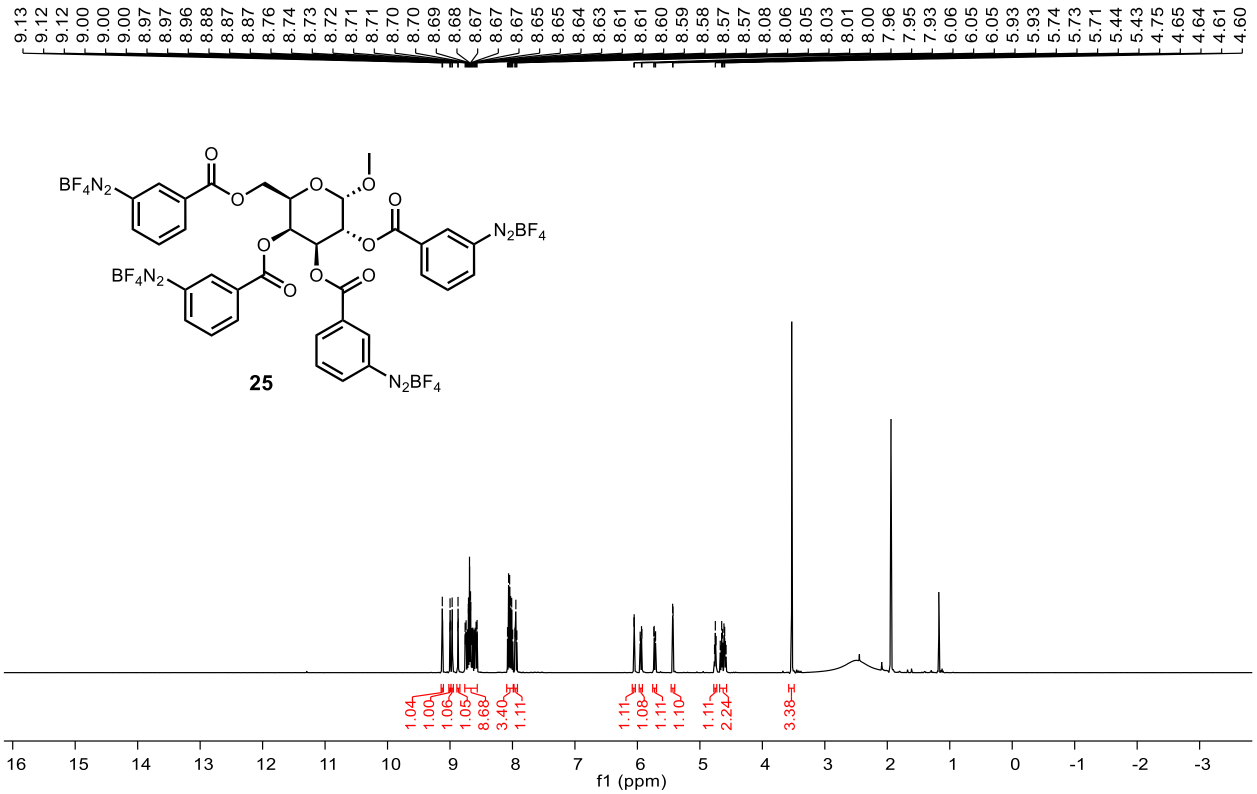

Figure S62a. ^{1}H NMR spectrum (400 MHz, CD_3CN) of **25**

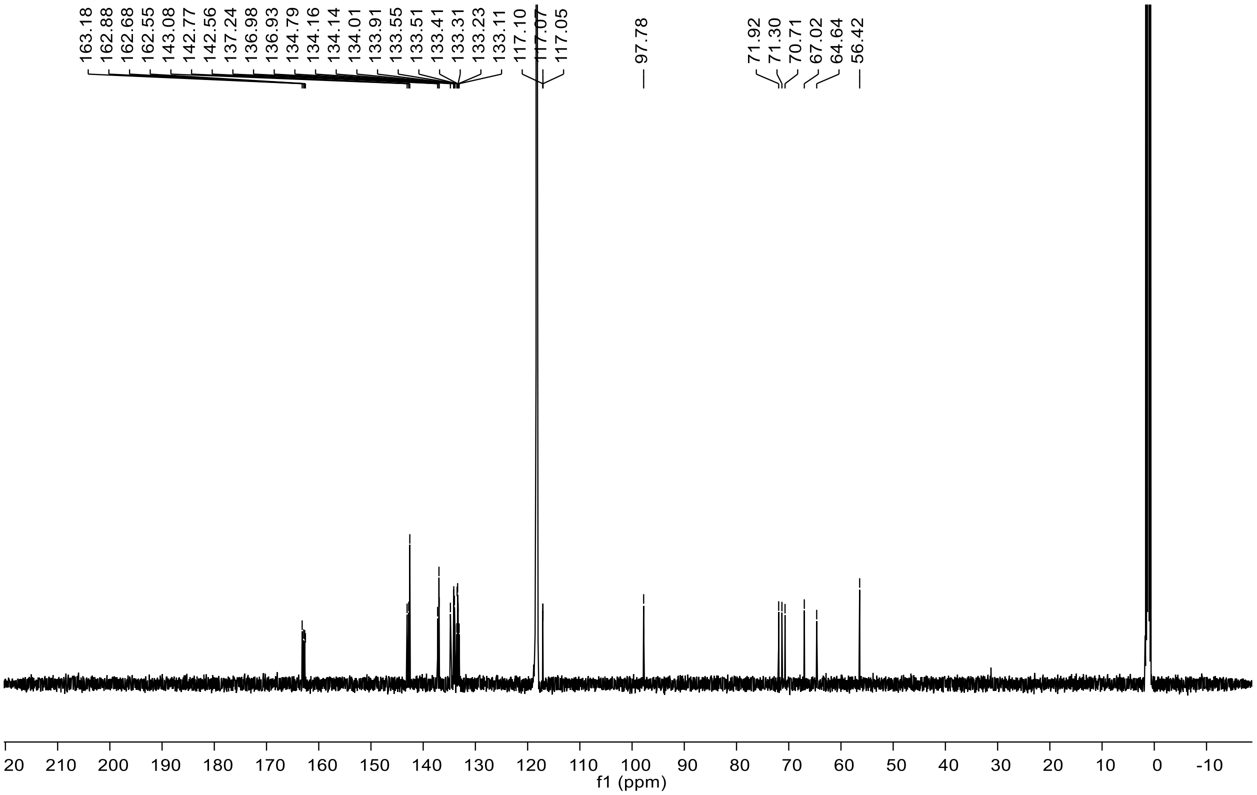

Figure S62b. ^{13}C NMR spectrum (126 MHz, CD_3CN) of **25**

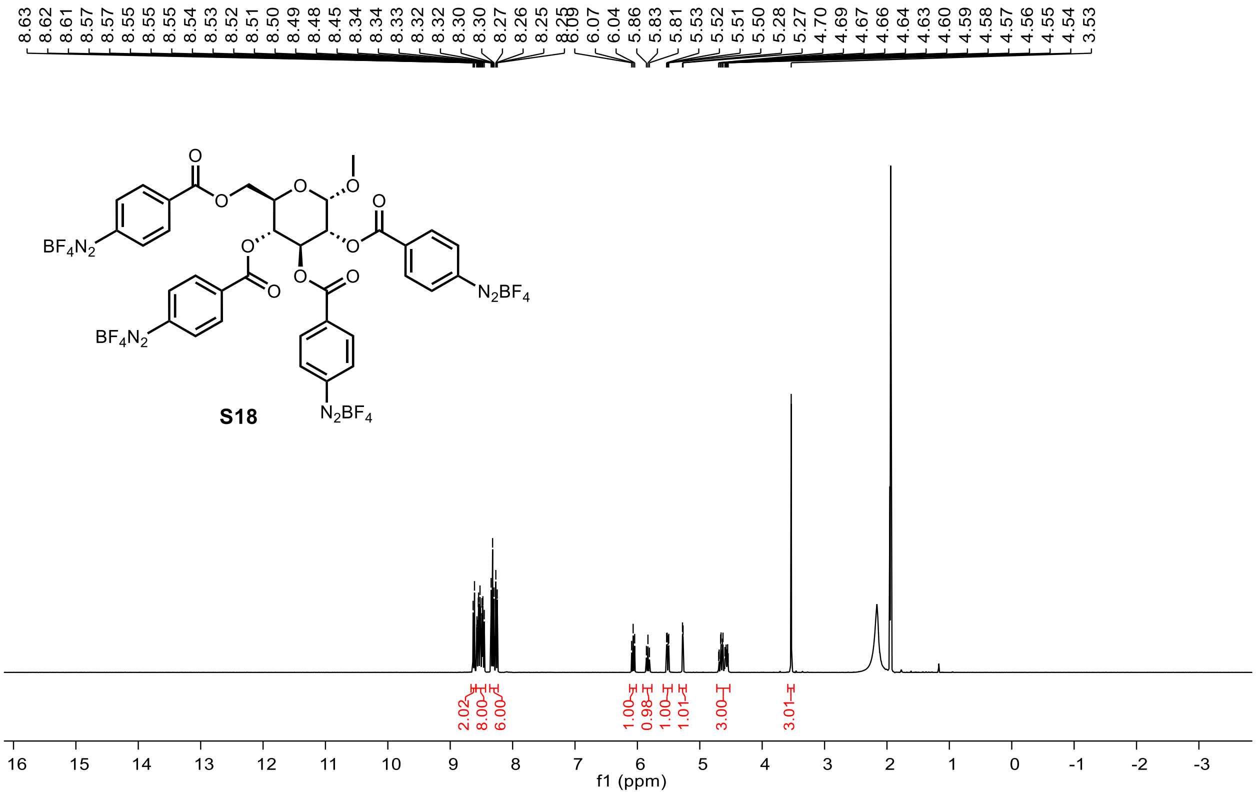

Figure S63a. ^{1}H NMR spectrum (400 MHz, CD_3CN) of **S18**

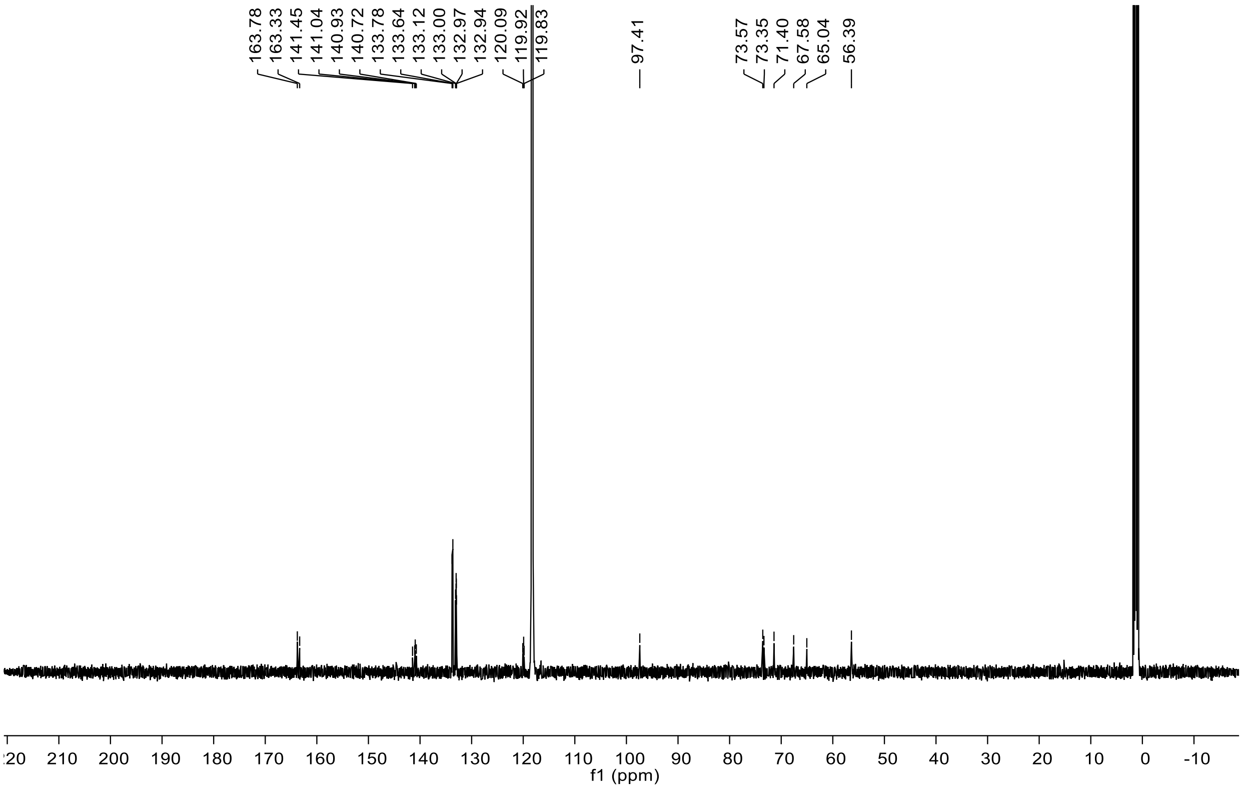

Figure S63b. ^{13}C NMR spectrum (126 MHz, CD_3CN) of **S18**

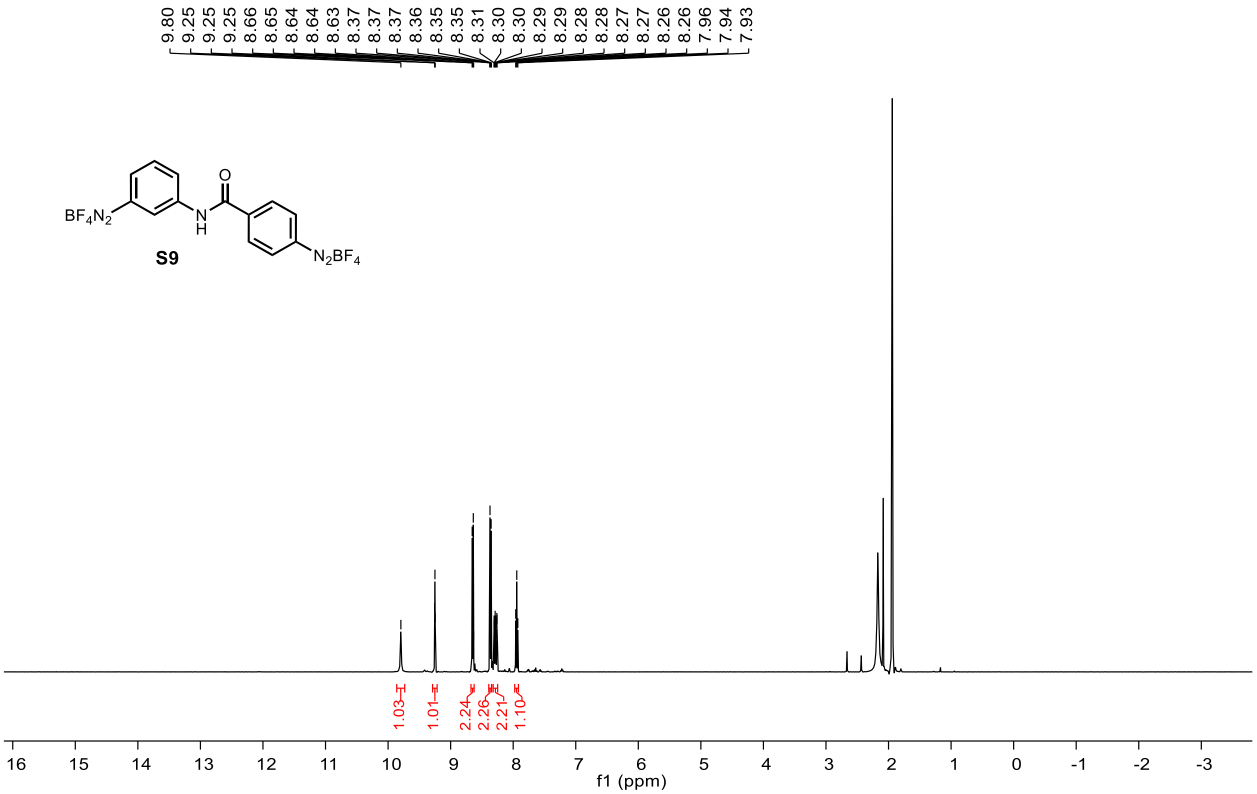

Figure S64a. ^{1}H NMR spectrum (500 MHz, CD_3CN) of **S9**

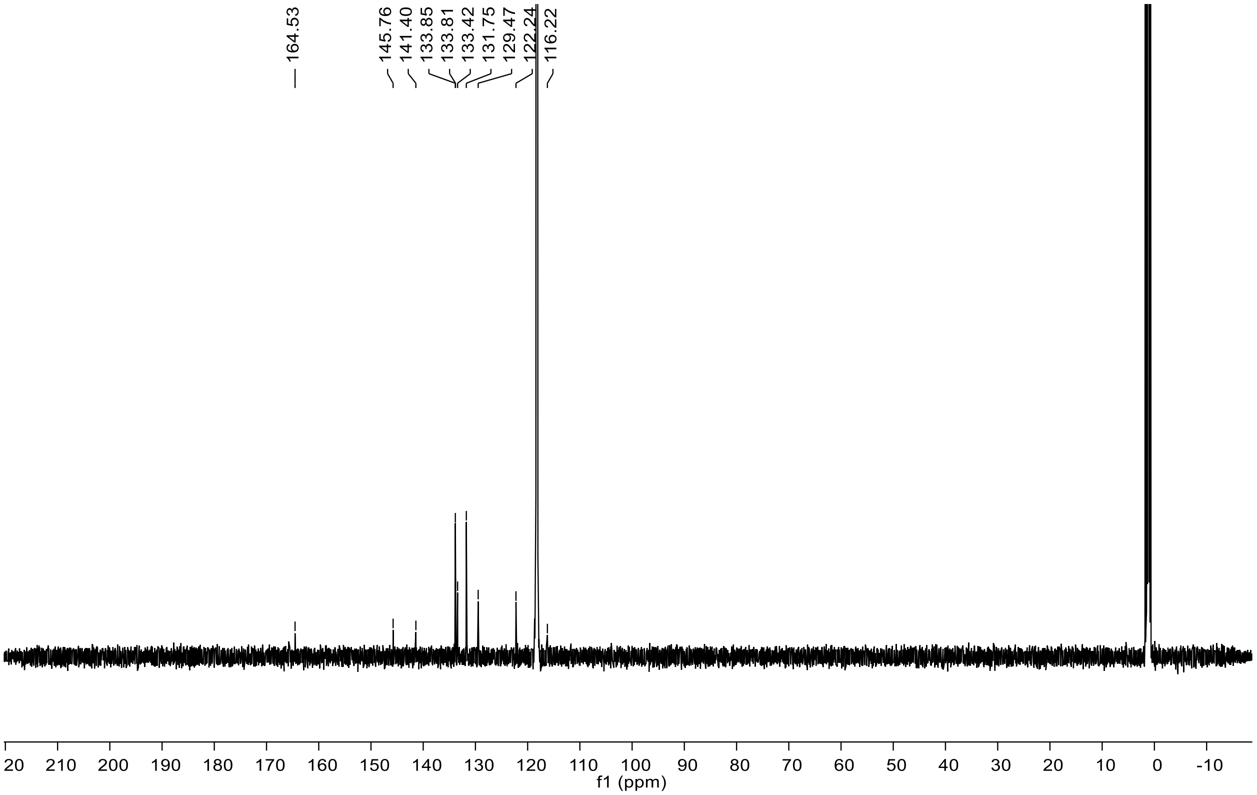

Figure S64b. ^{13}C NMR spectrum (126 MHz, CD_3CN) of **S9**

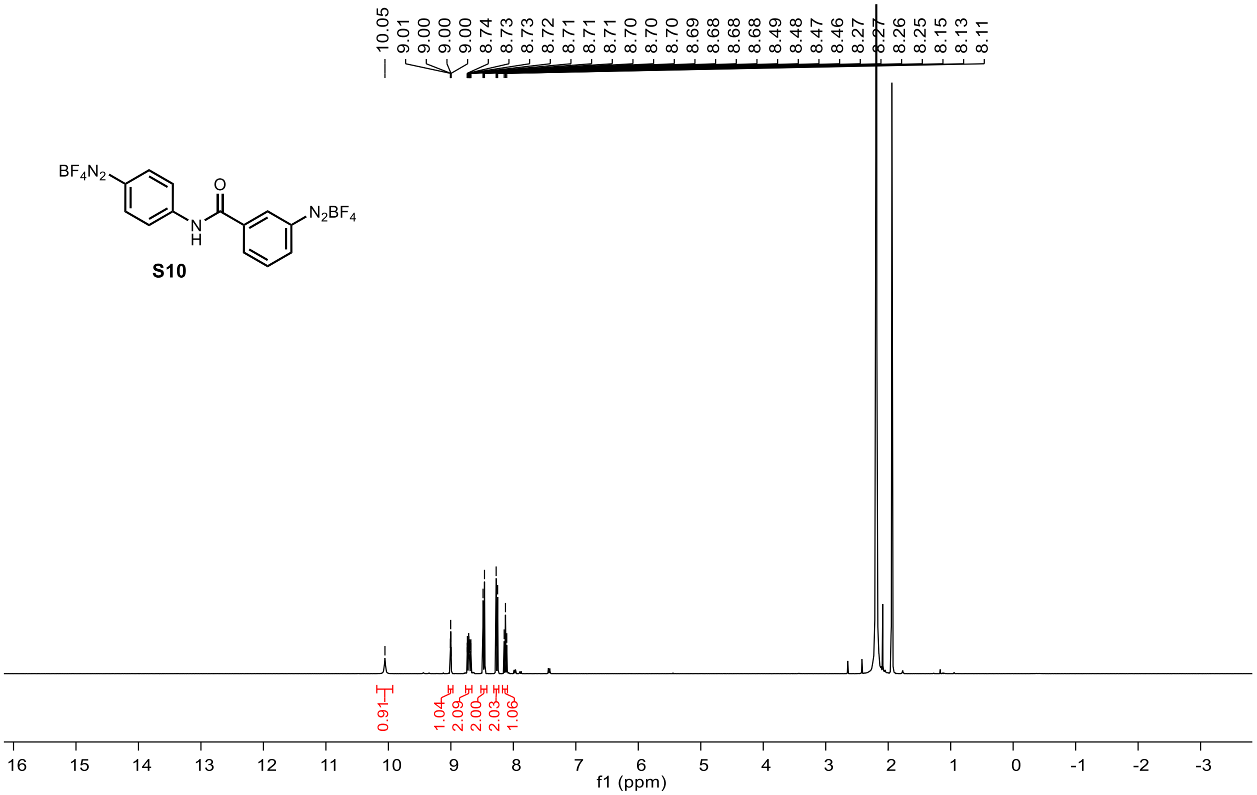

Figure S65a. ^{1}H NMR spectrum (400 MHz, CD_3CN) of **S10**

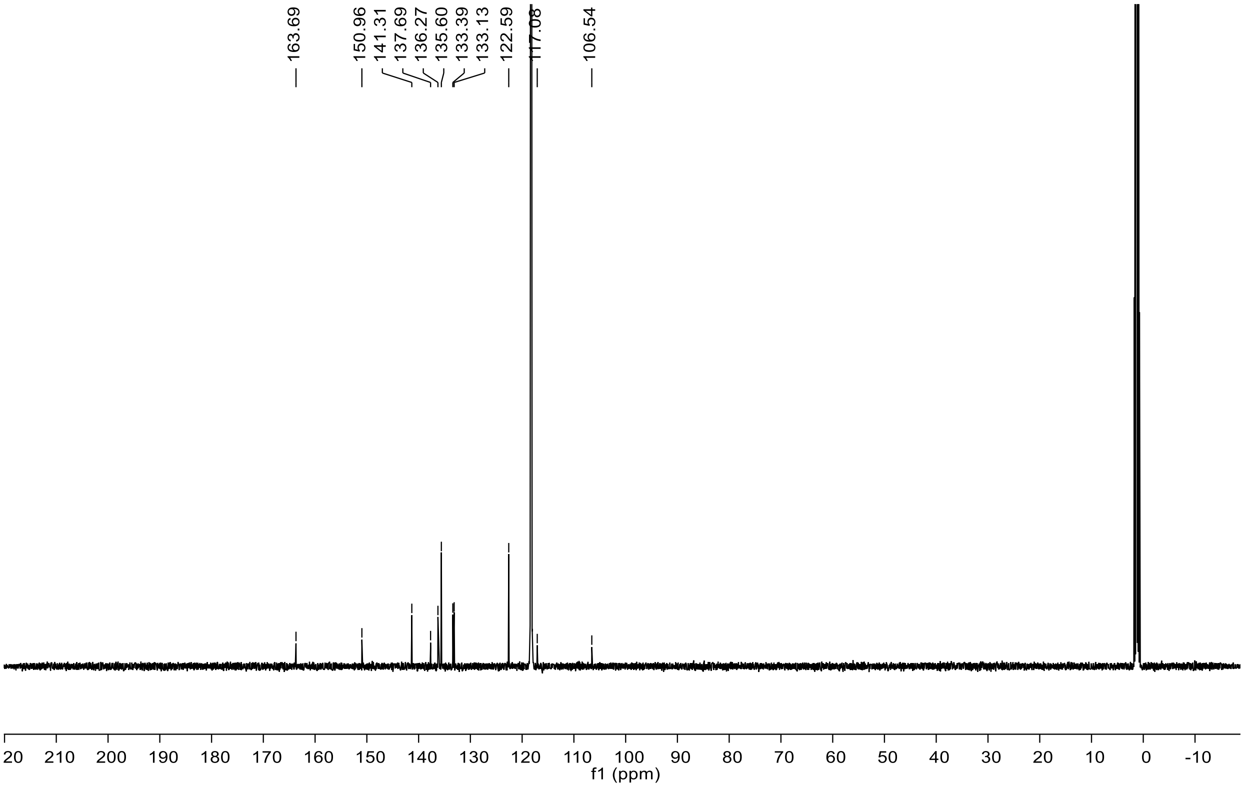

Figure S65b. ^{13}C NMR spectrum (126 MHz, CD_3CN) of **S10**

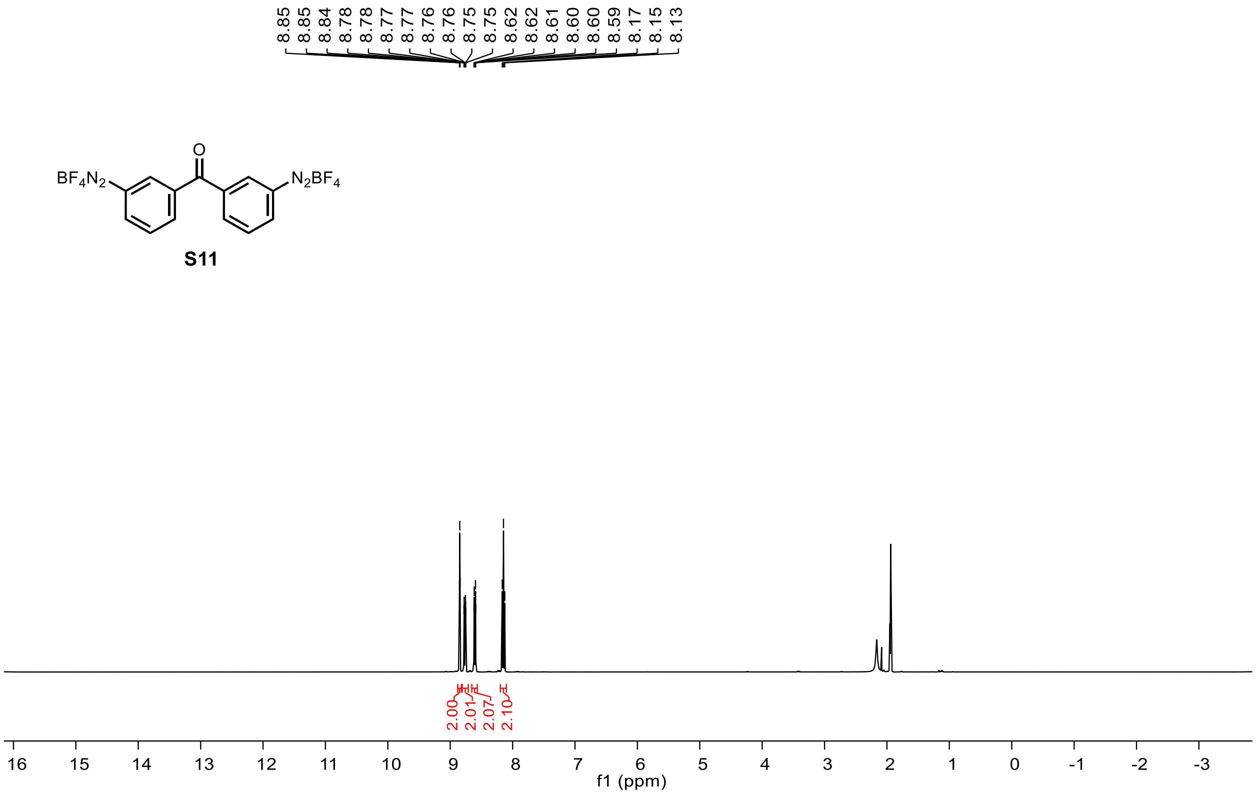

Figure S66a. ^{1}H NMR spectrum (400 MHz, CD_3CN) of **S11**

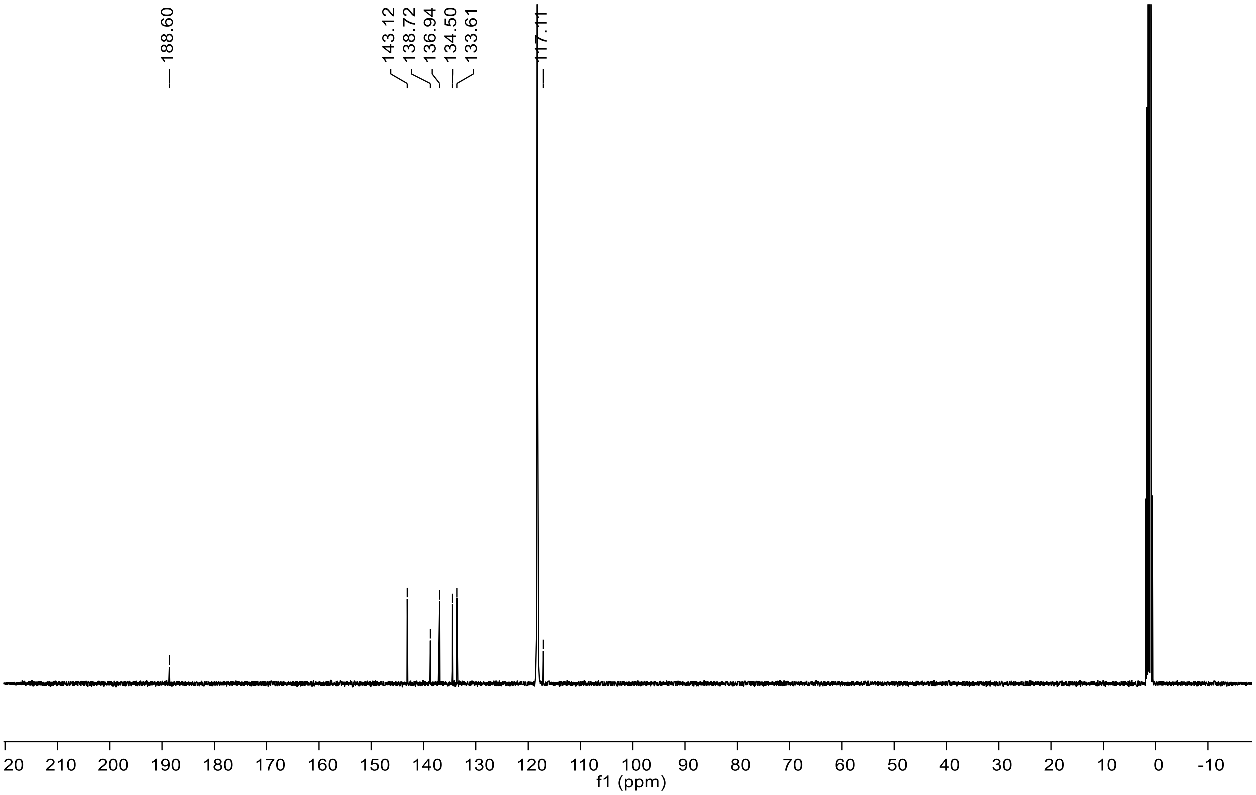

Figure S66b. ^{13}C NMR spectrum (126 MHz, CD_3CN) of **S11**

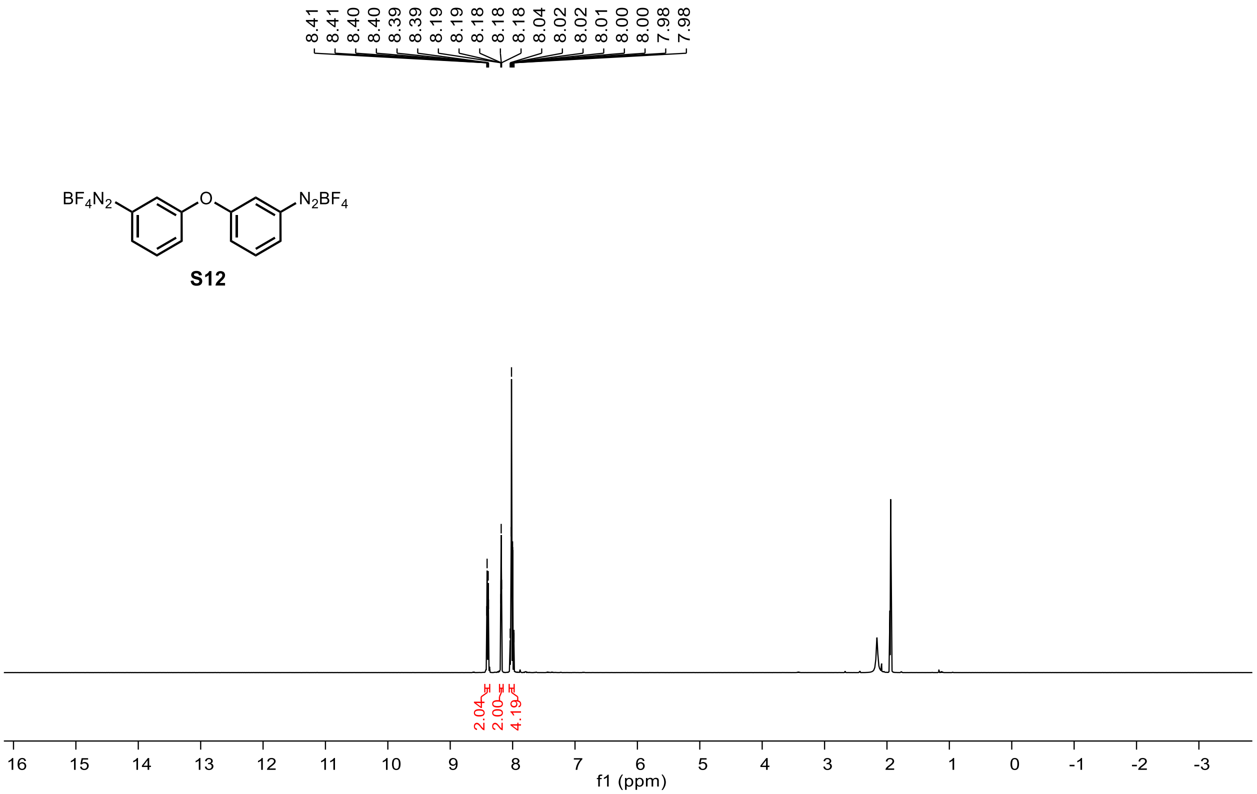

Figure S67a. ^{1}H NMR spectrum (400 MHz, CD_3CN) of **S12**

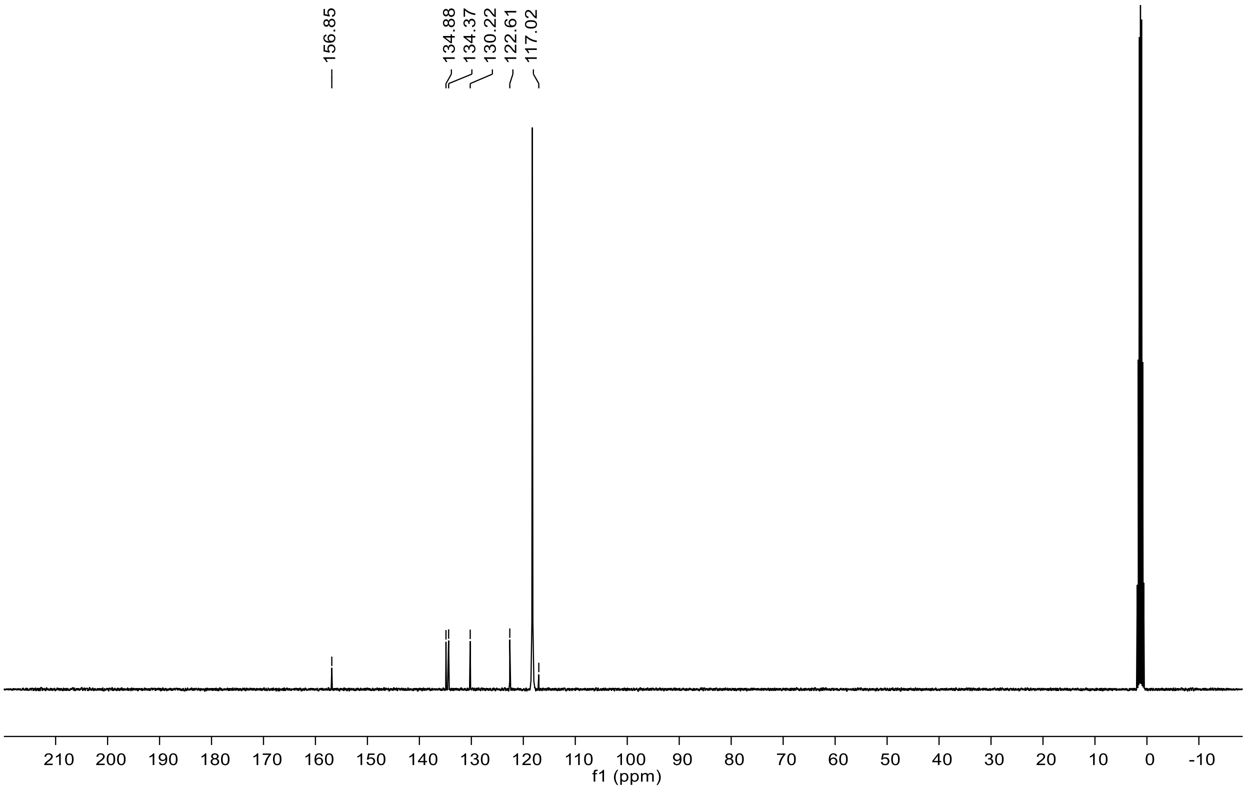

Figure S67b. ^{13}C NMR spectrum (126 MHz, CD_3CN) of **S12**

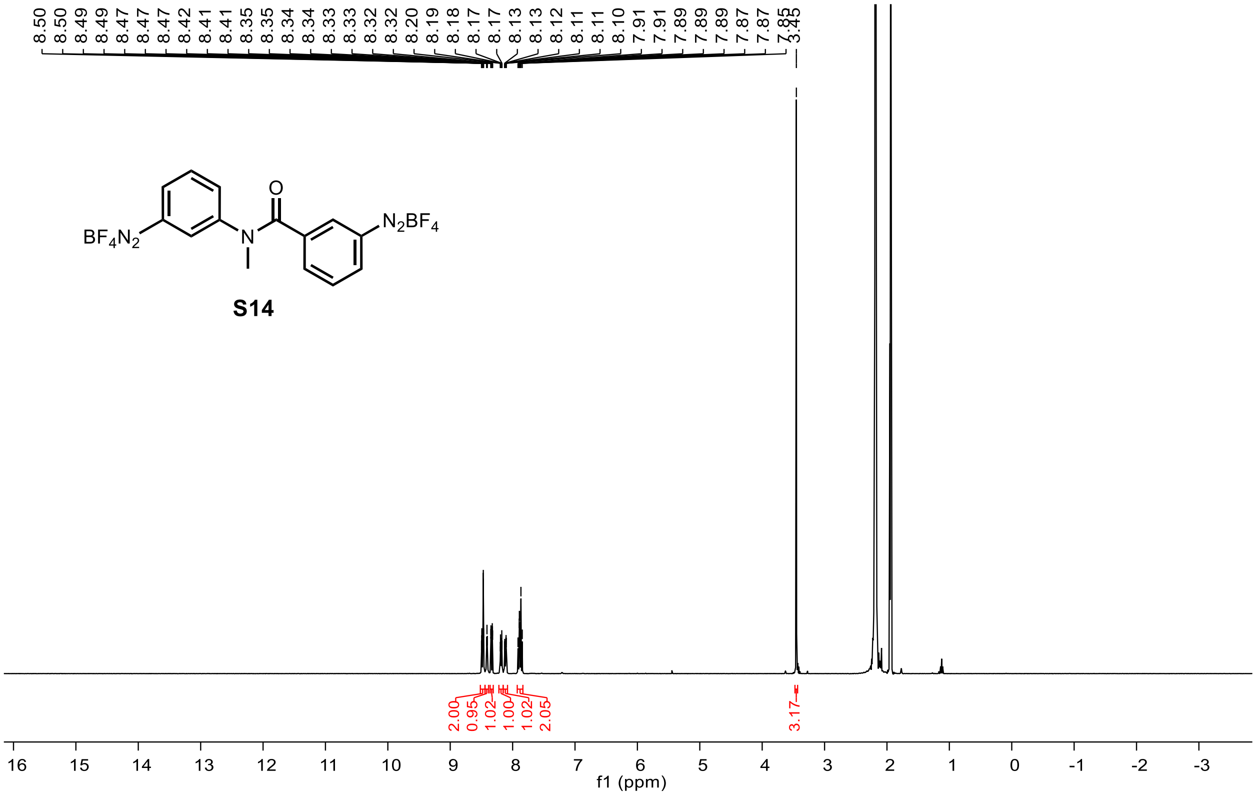

Figure S68a. ^{1}H NMR spectrum (400 MHz, CD_3CN) of **S14**

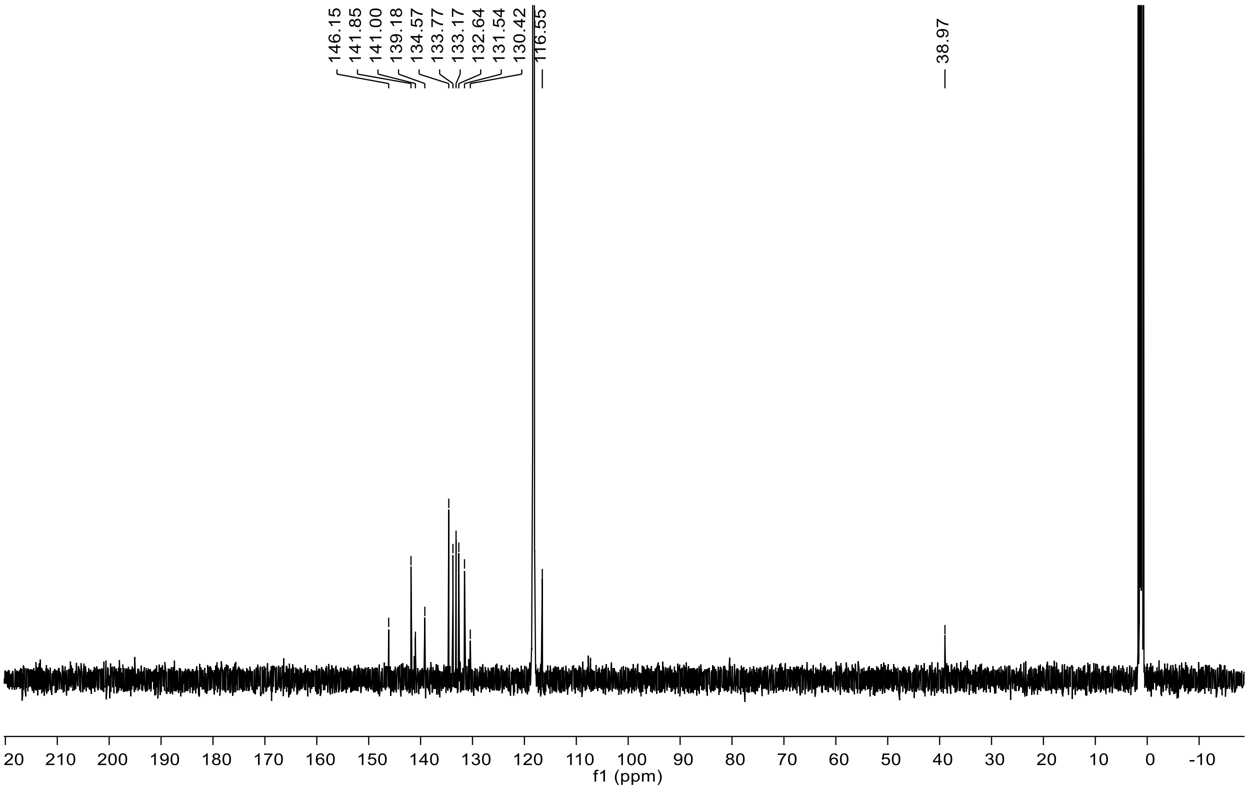

Figure S68b. ^{13}C NMR spectrum (126 MHz, CD_3CN) of **S14**

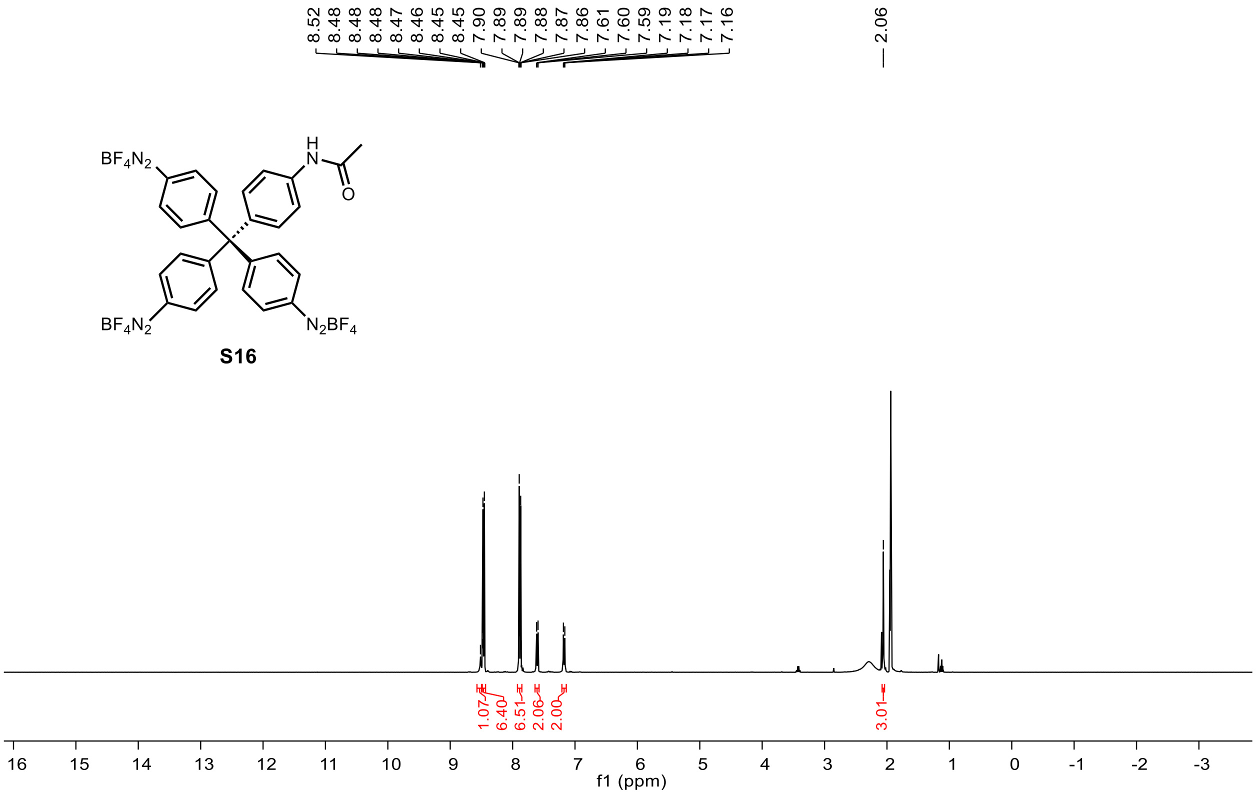

Figure S69a. ^{1}H NMR spectrum (400 MHz, CD_3CN) of **S16**

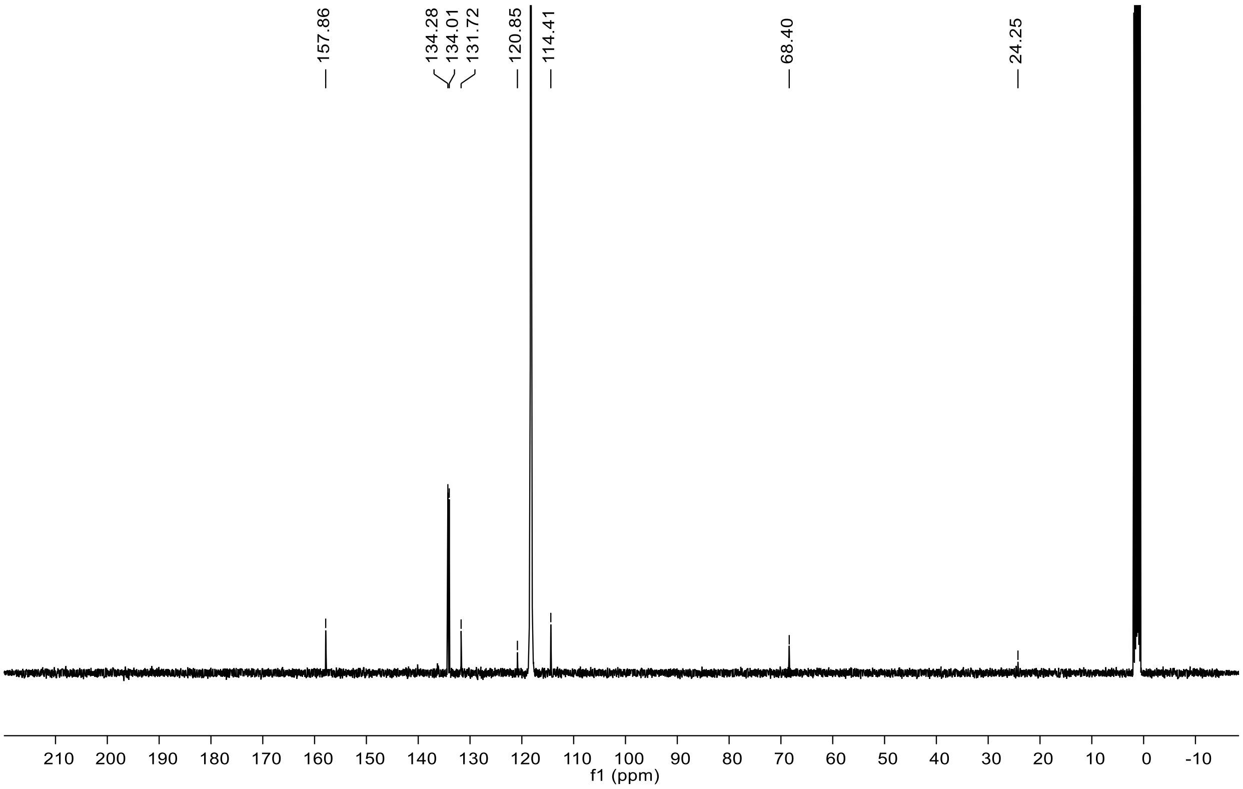

Figure S69b. ^{13}C NMR spectrum (126 MHz, CD_3CN) of **S16**

XV. References

---

[1] Pfaff, P.; Anderl, F.; Fink, M.; Balkenhohl, M.; Carreira, E., M. Azoacetylenes for the Synthesis of Arylazotriazole Photoswitches. *J. Am. Chem. Soc.* **2021**, *143*, 14495-14501.

[2] Heinrich, M. R.; Blank, O.; Ullrich, D.; Kirschstein, M. Allylation and Vinylation of Aryl Radicals Generated from Diazonium Salts. *J. Org. Chem.* **2007**, *72*, 9609–9616.

[3] Bremerich, M.; Conrads, C. M.; Langletz, T.; Bolm, C. Additions to N-Sulfinylamines as an Approach for the Metal-free Synthesis of Sulfonimidamides: O-Benzotriazolyl Sulfonimidates as Activated Intermediates. *Angew. Chem., Int. Ed.* **2019**, *58*, 19014–19020.

[4] Tabey, A.; Berlande, M.; Hermange, P.; Fouquet, E. Mechanistic and asymmetric investigations of the Au-catalysed cross-coupling between aryldiazonium salts and arylboronic acids using (P,N) gold complexes. *Chem. Commun.* **2018**, *54*, 12867–12870.

[5] Zhang, H.; Li, R.; Ba, S.; Lu, Z.; Pitsinos, E. N.; Li, T.; Nicolaou, K. C. DNA Binding and Cleavage Modes of Shishijimicin A. *J. Am. Chem. Soc.* **2019**, *141* (19), 7842-7852.
